# Salting out, non-ideality and synergism enhance surfactant efficiency in atmospheric aerosols

**DOI:** 10.1038/s41598-023-48040-5

**Published:** 2023-11-24

**Authors:** Manuella El Haber, Corinne Ferronato, Anne Giroir-Fendler, Ludovic Fine, Barbara Nozière

**Affiliations:** 1 Universite Claude Bernard Lyon 1, IRCELYON UMR 5256 CNRS, 69622 Villeurbanne, France; 2 KTH Royal Institute of Technology, 100 44, Stockholm, Sweden

**Keywords:** Atmospheric chemistry, Soft materials, Physical chemistry

## Abstract

In Earth’s atmosphere, the surface tension of sub-micron aerosol particles is suspected to affect their efficiency in becoming cloud droplets. But this quantity cannot be measured directly and is inferred from the chemical compounds present in aerosols. Amphiphilic surfactants have been evidenced in aerosols but experimental information on the surface properties of their mixtures with other aerosol components is lacking. This work explores experimentally the surface properties of aqueous mixtures of amphiphilic surfactants (SDS, Brij35, TritonX100, TritonX114, and CTAC) with inorganic salts (NaCl, (NH_4_)_2_SO_4_) and soluble organic acids (oxalic and glutaric acid) using pendant droplet tensiometry. Contrary to what could be expected, inorganic salts and organic acids systematically enhanced the efficiency of the surfactants rather than reduced it, by further lowering the surface tension and, in some cases, the CMC. Furthermore, all the mixtures studied were strongly non-ideal, some even displaying some synergism, thus demonstrating that the common assumption of ideality for aerosol mixtures is not valid. The molecular interactions between the mixture components were either in the bulk (salting out), in the mixed surface monolayer (synergy on the surface tension) or in the micelles (synergy on the CMC) and need to be included when describing such aerosol mixtures.

## Introduction

Although clouds are essential elements of the Earth atmosphere and climate^1^, predicting their formation remains a challenge today. Liquid cloud droplets are formed by the condensation of water onto aerosol particles, in which the surface tension, σ (mN/m) is expected to play a role^1,2^ especially for sub-micronic particles. Unfortunately, direct measurements of the surface tension of atmospheric particles remain unattainable today. Thus, until now, this quantity has been inferred from the surface tension properties of the chemical compounds present in atmospheric aerosols. According to the International Union for Pure and Applied Chemistry (IUPAC)^3^ compounds reducing the surface tension, for instance in aerosol particles, are defined as “surfactants”. By far the most abundant organic compounds in atmospheric aerosols are water-soluble ones, in particular organic acids (oxalic, succinic). But their adsorption isotherms^4–11^ show that their effects on the surface tension of aqueous mixtures are modest. This is even more true at activation, when the particles have taken up water and reached the critical size to become a cloud droplet, which corresponds to a dilution of the aerosol components by a factor 200–1000 (growth factor on the particle radius by a factor 6–10^1,12^). Over the last two decades amphiphilic surfactants have been evidenced in atmospheric aerosols^13–23^. Their adsorption isotherms (surface tension vs concentration curves) display two regions: a constant surface tension equal to that of pure water (σ_w_) at low concentration, and a constant surface tension with much lower value (σ_o_) at large concentration, with a sharp transition between the two regions, corresponding to the Critical Micelle Concentration (CMC). The concentration of amphiphilic surfactants in atmospheric PM1 aerosols was found to be typically of the order of 0.1 M and their CMC between 10^−5^ and 10^−4^ M^18,19^. Thus, even at activation, i.e. after a dilution by a factor 200 to 1000, their concentration remains mostly above the CMC, thus maintaining the surface tension at the minimal value σ_o_.

A number of models have been developed to predict the effects of amphiphilic surfactants on cloud droplet formation^24–27^ or to predict the surface tension resulting from these compounds and their mixtures^28^. However, because of the complex chemical composition of atmospheric particles, their surface tension would not only be affected by the amphiphilic surfactants but also by the presence of other, major components such as inorganic salts and organic acids. But experimental information on the properties of mixtures of amphiphilic surfactants with these aerosol components is largely unavailable. While some studies have investigated mixtures of amphiphilic surfactants with inorganic salts, few have reported direct surface tension measurements. Another important piece of information when predicting the surface tension of atmospheric particles is whether aerosol mixtures behave ideally^24,25^ or involve molecular interactions^26,27^, which also requires experimental information.

The objective of the present work is to study experimentally the effects on the surface tension of mixing amphiphilic surfactants with major aerosol components, inorganic salts and organic acids. For this, the adsorption isotherms for mixtures of amphiphilic surfactants (Sodium Dodecyl Sulfate or “SDS”, Brij35, TritonX100, TritonX114, and CetylTrimethyl Ammonium Chloride or “CTAC”) with inorganic salts (NaCl, (NH_4_)_2_SO_4_) and organic acids (ethanedioic or “oxalic”, pentanedioic or “glutaric”) were determined using drop-shape analysis. Examining the evolution of the surface tension and CMC in these isotherms with the mixture composition (molar fraction) allowed to investigate the ideality of the mixtures. In this discussion, a number of definitions recommended by the IUPAC^3^ will be used:

- *Ideal mixture*: mixture for which a property (in this work, the surface tension or CMC) varies linearly with the molar fraction of the mixture, between the values for the pure components. In ideal mixtures, each component contributes separately to the property of interest, without interacting molecularly, and the mixture property is the sum of the contributions from each component.
- *Non-ideal mixture*: mixture for which a property deviates from the linear variation with the molar fraction. Non-ideality indicates the existence of molecular interactions between the components of the mixture.
- *Synergism*: extreme case of non-ideality where a property of interest deviates so much from linearity that it exceeds (or, in the case of the surface tension or CMC, is lower than) the values for the pure components.
- *Antagonism*: opposite case of synergy, a property is strongly non-ideal and reaches values that are lower than (or, in the case of the surface tension or CMC, is larger than) those for the pure components.

Although the hanging drop method used in this work for the surface tension measurements might not capture the partitioning effects predicted for small particles^29^ it is more amenable for the determination of over 70 isotherms in this work, than the techniques measuring individual particles, such as optical tweezers^30,31^. The isotherms reported in this study provide robust surface tension data to validate CCN models, to which partitioning can be numerically added a posteriori. Furthermore, working on larger samples might reveal the existence of molecular interactions that might be too weak to detect on single particles.

## Results and discussion

The experimental procedure is described in “Experimental” Section. The adsorption isotherms were established for various ternary mixtures “compound A/compound B/water”, in which x is the molar fraction relative to the A–B mixture, and α the molar fraction of A or B in water. A complete list of the experiments is given in Table S1 of the Supplementary Information (SI) and the complete results (numerical data for the isotherms obtained) are presented both as tables and graphs in Sections S3 to S7.

### The impact of inorganic salts on amphiphilic and water-soluble surfactant properties

The adsorption isotherms for mixtures of NaCl and (NH_4_)_2_SO_4_ with the amphiphilic surfactants TritonX100 and Brij35 were determined as well as, for comparison, those of glutaric acid with the same salts. The isotherms obtained (Figs. S1–S4, S6 and S7) show that, in all cases, adding inorganic salts enhances the surfactant efficiency. This result is not intuitive, as inorganic salts alone increase the surface tension of aqueous solutions. The entire isotherms for the glutaric acid mixtures (Figs. S6 and S7) are shifted towards lower surface tension values as the salt concentration increases. For a given concentration of glutaric acid the decrease of the surface tension with salt concentration appears to be linear (Fig. S8). For instance, for [glutaric acid] = 0.5 M the slope of this diminution in the presence of NaCl is Δσ = − (1.9 ± 1.0) mNm^−1^ M^−1^ and nearly twice as large with (NH_4_)_2_SO_4_, Δσ = − (3.2 ± 1.5) mNm^−1^ M^−1^ (Fig. S8). Similar decrease of the surface tension with salt concentration are reported in the literature for mixtures of NaCl, (NH_4_)_2_SO_4_ and other salts with succinic acid^32^, citric acid^33^, cis-pinonic acid^34,35^, Nordic Aquatic Fulvic Acid Reference (NAFA)^36^, humic and fulvic substances^37^, and limonene-derived organosulfates^38^. Some works did not observe any surface tension reduction for mixtures of (NH_4_)_2_SO_4_ with cis-pinonic acid, Nordic reference fulvic, acid oxalic acid dihydrate, succinic, and adipic acid^33^. But, based on the curves in Fig. S8, these effects were likely to have been below the measurement limit for the very dilute (NH_4_)_2_SO_4_ solutions used in that study (< 0.15 molkg^−1^).

**Figure 1:**
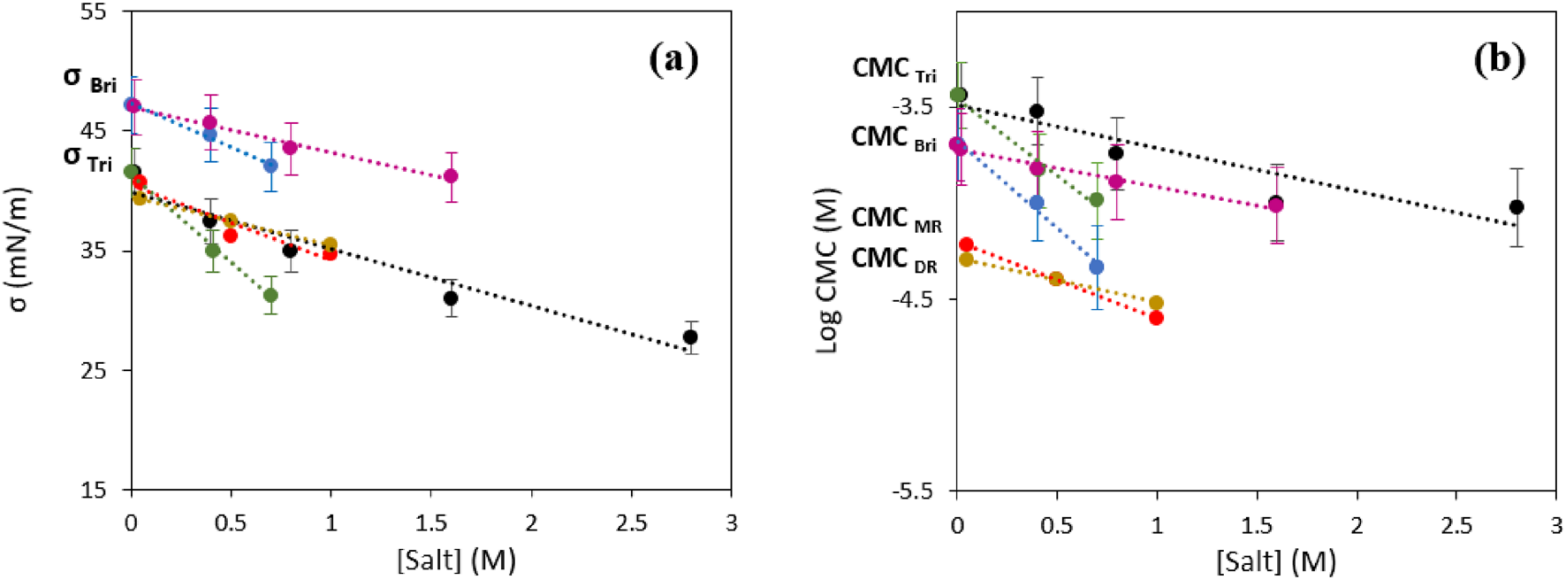
Effects of inorganic salts on the surface properties of amphiphilic surfactants. (**a**) Variation of the surface tension for mixtures: (black filled circle) TritonX100 at 10^−4^ M + NaCl, (green filled circle) TritonX100 at 10^−4^ M + (NH_4_)_2_SO_4_, (purple filled circle) Brij35 at 10^−4^ M + NaCl, (blue filled circle) Brij35 at 10^−4^ M + (NH_4_)_2_SO_4_, and comparison with (yellow filled circle) Di-rhamnolipid at 10^−5^ M + NaCl and (red filled circle) Mono-rhamnolipid at 10^–5^ M + NaCl from Ref^39^ (**b**) Variation of the CMC with salt concentration for amphiphilic surfactants: (black filled circle) TritonX100 + NaCl, (green filled circle) TritonX100 + (NH_4_)_2_SO_4_, (Purple filled circle) Brij35 + NaCl, (blue filled circle) Brij35 + (NH_4_)_2_SO_4_ and comparison with (yellow filled circle) Di-rhamnolipid + NaCl and (red filled circle) Mono-rhamnolipid + NaCl from Ref^39^.

The mixtures of inorganic salts with TritonX100 and Brij35 displayed similar trends, the entire isotherms being shifted towards lower surface tension values as the salt concentration increases (Figs. S1–S4). Because of the shape of these isotherms the maximum surface tension reduction takes place just below the CMC ([TritonX100] = 10^−4^ M and [Brij35] = 5 × 10^−5^ M). For the surfactants studied in this work, the diminution of σ appears to be linear with salt concentration (Fig. 1a). For [TritonX100] = 10^−4^ M the slope was Δσ (mNm^−1^ M^−1^) = − (6.0 ± 1.0) with NaCl, and − (15.0 ± 2.5) with (NH_4_)_2_SO_4_. For [Brij35] = 5 × 10^−5^ M it was Δσ (mNm^−1^ M^−1^) = − (7.8 ± 2.0) with (NH_4_)_2_SO_4_ and − (4.0 ± 1.0) with NaCl. These results thus confirm the trend observed with glutaric acid, that (NH_4_)_2_SO_4_ is about twice as efficient as NaCl in reducing the surface tension. Beyond the CMC, i.e. for σ_o_, the diminution of the surface tension was less pronounced, with Δσ being 1/2–1/3 of the values reported above (Figs. S1–S4).

The CMC of the mixtures with amphiphilic surfactants was also lowered when increasing salt concentration, following log-linear trends (Fig. 1b): for TritonX100 with NaCl ΔlnCMC = − (0.5 ± 0.2) and with (NH_4_)_2_SO_4_ ΔlnCMC = − (1.8 ± 1.0); for Brij35 with (NH_4_)_2_SO_4_ ΔlnCMC = − (2.1 ± 1.0) and with NaCl ΔlnCMC = − (0.5 ± 0.2). These results show that, in all cases, (NH_4_)_2_SO_4_ is also twice as efficient in lowering the CMC than NaCl.

Previous studies of mixtures of NaCl with SDS^40,41^, CTAB^42^, AOS^43,44^, Sodium decanoate^45^, and mono- and di-rhamnolipids (the later being represented in Fig. 1)^39^ also reported a decrease both of the surface tension and the CMC upon addition of salt, and over a wide range of salt concentration relevant to aerosols^42^. These previous works also reported that, while with non-ionic surfactants (as those studied in this work) the diminution of σ is linear, it is not linear with cationic or anionic surfactants^42^.

These results suggest that, in atmospheric aerosols, the efficiency of amphiphilic surfactants should be significantly enhanced by the presence of NaCl or (NH_4_)_2_SO_4_. For instance, for a typical concentration of [(NH_4_)_2_SO_4_] = 3 M and assuming slope of Δσ ~ − 5 mNm^−1^ M^−1^ for σ_o_ in TritonX100 + (NH_4_)_2_SO_4_ mixtures would imply a diminution of σ_o_ by ~ 15 mN m^−1^, which is significant and should contribute to increase the cloud-forming efficiency of the aerosol particles.

In a second part of the analyses, the variations of σ_o_ for the mixtures with amphiphilic surfactants were traced as function of the molar fraction for the mixture (Fig. S5). Note that, in these plots, it was not possible to obtain experimental data at large salt concentration because the droplets produced were unstable and their surface tension could not be measured. But even with a limited number of points it is clear in these plots that the mixtures are significantly non-ideal (large deviations of σ_o_ from the linear variations), which indicates the existence of molecular interactions between the inorganic ions and the surfactant molecules.

These effects are well documented^46^ to result from the “salting out” of the organic molecules towards the surface. The molecular interactions involved are strong electrostatic ones, in which the inorganic ions form solvation “cages” around the water molecules in the bulk. The surfactant being “pushed” to the surface by the inorganic ions, their larger surface concentration results in a lower surface tension than in the absence of salt. It also implies that surface saturation is reached with lower surfactant concentration, thus that the CMC is shifted to lower concentrations. Different anions and cations have different solvating efficiencies. In particular SO_4_^2−^ and NH_4_^+^ have stronger solvating and salting out effects than Cl^−^ and Na^+^^46^, thus resulting in the stronger efficiency of (NH_4_)_2_SO_4_ than NaCl in reducing the surface tension, consistent with the observations in this work.

The results of this work thus demonstrate that, contrary to the expected effects of inorganic salts on surface tension, their presence in aerosols would further enhance the properties of amphiphilic surfactants by lowering both the surface tension and the CMC. In addition, these results show that, while ideality is the most common assumption for aerosol mixtures, the mixtures of amphiphilic surfactants and inorganic salts are strongly non-ideal. This might account for some discrepancies reported in previous works. Cloud Condensation Nuclei Models (CCN-models) using the assumption of ideal mixtures have reported significant deviations from measurements for TritonX100/NaCl and (NH_4_)_2_SO_4_ mixtures, which can be attributed to salting out^24^. Other models, also using the ideal assumption, did not report any differences in the predictions of the CCN ability of SDS in the presence and in the absence of NaCl, but did not compare with experimental data^25^. The large effects of salting out in atmospheric aerosols suggested by our results indicate that such effects need to be further investigated, in particular in models.

### Amphiphilic surfactants and water-soluble acids

**Figure 2:**
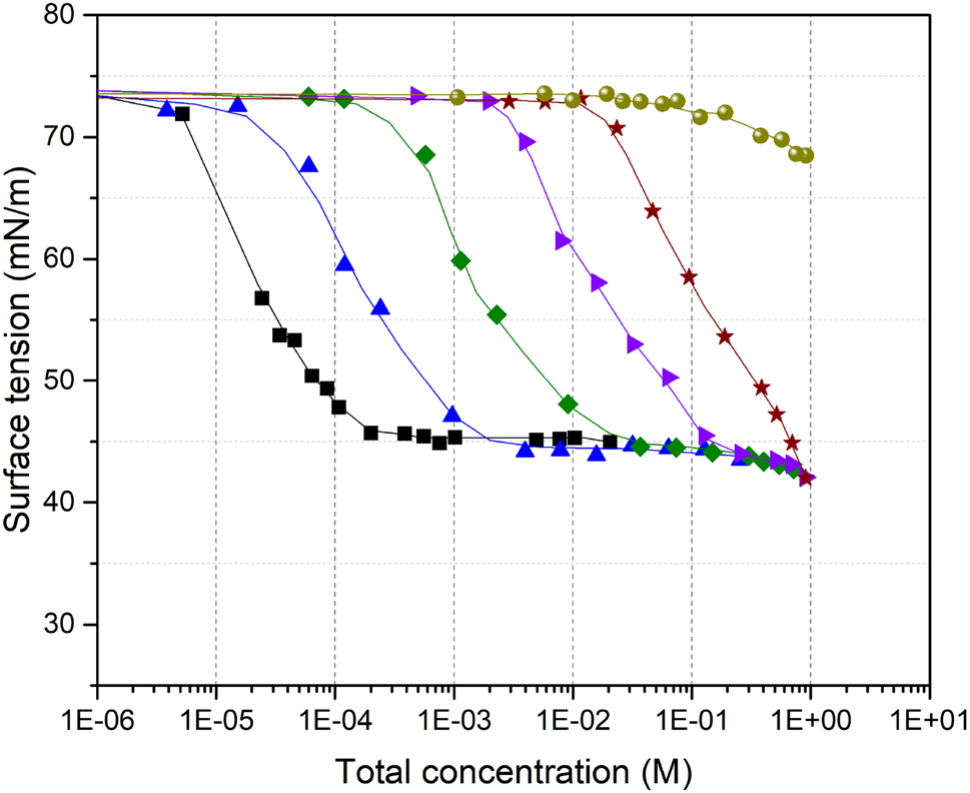
Adsorption isotherms for Brij35 + oxalic acid mixtures: (black filled square) black curve: Brij35; (blue filled triangle) blue curve: x = 0.091; (green filled diamond) green curve: x = 0.0099; (violet filled right angle) violet curve: x = 0.00111; (wine filled star) wine curve: x = 0.00018 and (yellow filled circle) yellow curve: Oxalic acid; Total concentration = [Brij35] + [Oxalic acid].

**Figure 3:**
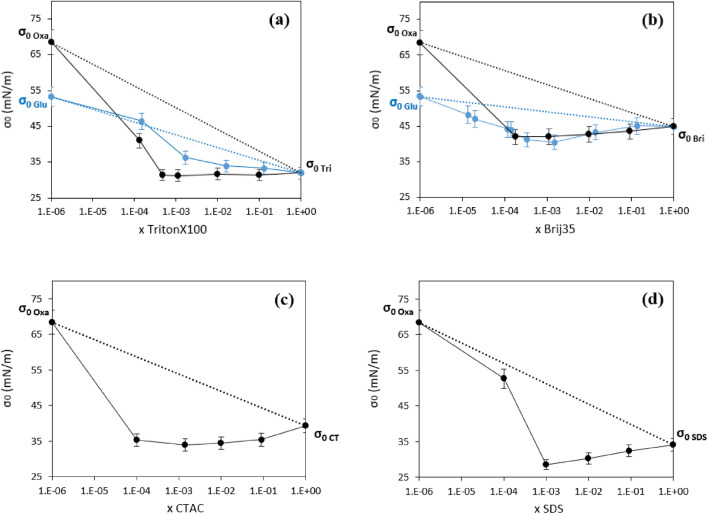
Evolution of the minimal surface tension for mixtures of amphiphilic surfactants and organic acids. (**a**) (blue filled circle) TritonX100 + Glutaric acid, (black filled circle) TritonX100 + Oxalic acid; (**b**) (blue filled circle) Brij35 + Glutaric acid, (black filled circle) Brij35 + Oxalic acid; (**c**) (black filled circle) CTAC + Oxalic acid, (**d**) (black filled circle) SDS + Oxalic acid.

The isotherms of mixtures of TritonX100, TritonX114, Brij35, SDS and CTAC with oxalic and glutaric acid were investigated and the results are presented in Figs. 2 and S9–S17. Some of these isotherms were established by adding a constant acid concentration to the surfactant, while others were established by maintaining a constant organic molar fraction, x, between the surfactant and the organic acid. Plotting the variations of σ_o_ with the molar fraction for all these mixtures (Fig. 3) showed that, while σ_o_ globally increased with the organic acid concentration, its variations displayed large deviations (between 5 and 30 for mN m^−1^) from the linear variations, thus showing the non-ideality of these mixtures. Some of these mixtures even display a minimum value for σ_o_, which was lower (by 1–10 mN m^−1^) than the σ_o_ for the pure compounds, which is defined as synergism. However, the CMC of all these mixtures varied linearly with the molar fraction (Fig. S18), thus indicating an ideal behavior with respect to the CMC.

Little data is available in the literature on the effect of water-soluble organic compounds on the surface tension of mixtures of amphiphilic surfactants. The adsorption isotherm for one mixture of TritonX100 with glutaric acid has been measured from single particles in an optical tweezer^31^, but was not compared with other molar fractions nor with the isotherm for TritonX100 alone. Isotherms for mixtures of SDS and CTAB with ascorbic acid and its derivatives were also studied^47,48^, and discuss the different molecular interactions occurring in these mixtures.

Non-idealities and synergistic effects on σ_o_ such as those observed in this work are well known and have been extensively studied in the surfactant literature^46^. But they are established for the first time for atmospherically-relevant mixtures in this work. These effects are attributed to molecular interactions taking place in the mixed monolayer at the surface of the mixture, rather than electrostatic interactions in the bulk. These molecular interactions can be of various nature, such as ion–dipole, dipole–dipole^47,48^, dipole-induced dipole, or hydrogen bonding^46,49,50^. In this work, the non-ideality observed with non-ionic surfactants suggest that these effects result rather from weakly ionic or non-ionic interactions (dipole–dipole). A potential model to describe the non ideality in such mixtures will be discussed in “Prediction and description of non-ideality, synergistic and antagonistic effects” Section.

In aerosols, the large deviations of σ_o_ from ideality observed in this work in the presence of organic acids (− 20 to − 30 for mN m^−1^) would also contribute to significantly reduce the surface tension of atmospheric particles and enhance cloud droplet formation.

### Mixtures of two amphiphilic surfactants

The amphiphilic surfactants extracted from atmospheric aerosols are complex mixtures of different surfactants. The isotherms established for these extracts thus display net effects resulting from all the interactions between these surfactants. For the sake of simplicity in predicting the surface tension of atmospheric aerosols, it might be better to consider these surfactant mixtures as a whole and estimate their surface properties from their overall isotherms. Understanding the many interactions between different amphiphilic surfactants is beyond the scope of atmospheric chemistry and has been studied extensively in the field of surfactant science^46^. However, for the purpose of illustrating the type of surface properties of such mixtures, some have been studied in this work.

**Figure 4:**
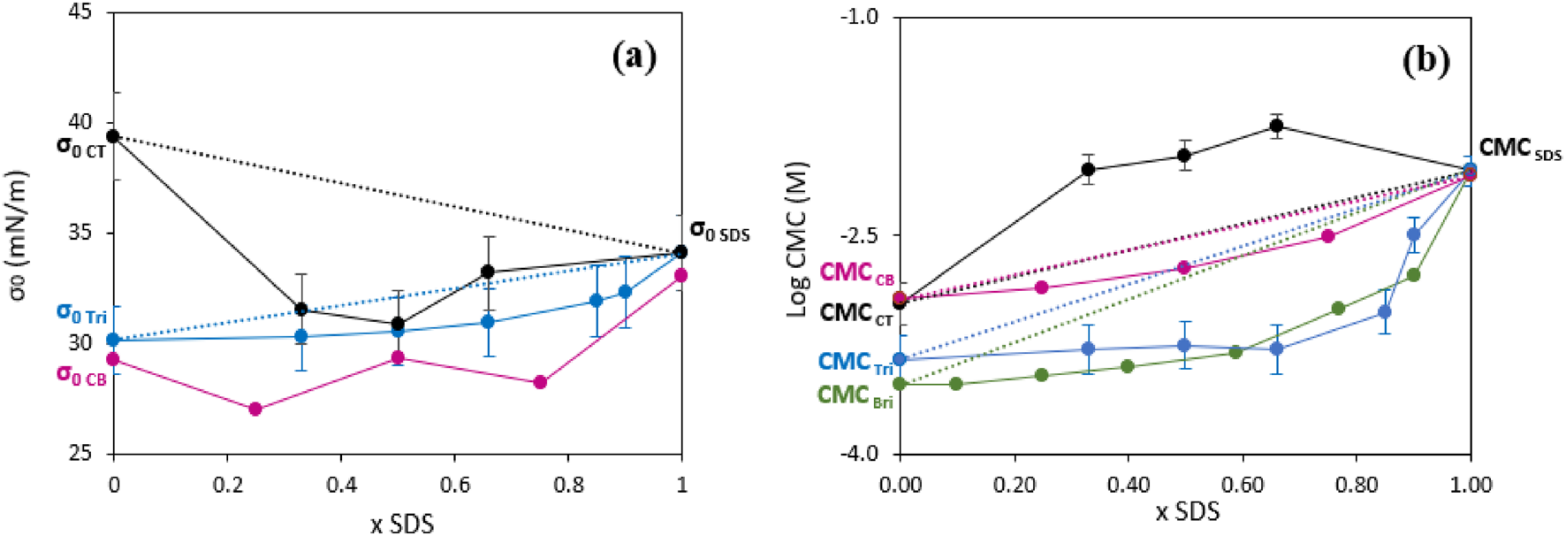
Evolution of the minimal surface tension and CMC in mixtures of different amphiphilic surfactants. (**a**) minimal surface tension for: (blue filled circle) SDS + TritonX114, (black filled circle) SDS + CTAC and comparison with (purple filled circle) SDS + CTAB from ref.^54^; (**b**) CMC for: (blue filled circle) SDS + TritonX114; (black filled circle) SDS + CTAC and comparison with (purple filled circle) SDS + CTAB from ref.^54^ and (green filled circle) SDS + Brij35 from ref.^55^

The adsorption isotherms for mixtures of SDS with TritonX114 and CTAC were determined and are presented in Section S6. As shown in Figs. S19 and S20, both the shape and position of these isotherms were significantly affected by the mixture composition. Plotting the variations of σ_o_ with the molar fraction for mixtures for SDS + TritonX114 (Fig. 4) showed that σ_o_ deviates little from ideality. But in mixture SDS + CTAC σ_o_ displays larger deviations from ideality, with Δσ =− 8 mN m^−1^, and even a synergistic effect (σ_o_ lower than that for SDS) of about 3 mN m^−1^ (Fig. 4a). In these mixtures, the variations of the CMC also displayed strongly non-ideal behaviors (Fig. 4b). For the mixtures SDS + TritonX114, this effect was positive, the CMC being lower than expected for an ideal mixture. But for SDS + CTAC the CMC of the mixture was larger than expected for an ideal mixture, and even larger than the CMC for SDS and CTAC, which is defined as an antagonistic effect.

Synergistic and antagonistic effects on σ_o_ and/or the CMC have been reported for hundreds of amphiphilic surfactant mixtures in the literature^46,51^. For instance, synergism on the surface tension was reported for mixtures SDS + Brij30, CTAB + Brij30^52^, SDS + DDAO (N,N-dimethyldodecylamine N-oxide)^53^, and SDS + CTAB (represented in Fig. 4a).^54^ Synergism on the CMC was reported for SDS + DDAO (N,N-dimethyldodecylamine N-oxide)^53^ and for SDS + Brij35^55^ (represented in Fig. 4b). Antagonism on the CMC was reported for TritonX100 + Brij35^56^.

Synergism on the CMC has been extensively studied in the surfactant literature and is attributed to the formation of mixed micelles, i.e. including the two types of molecules^46,51^. Antagonistic effects on the CMC are attributed to competition or steric hindrance between the two surfactants during the formation of the micelles^46,51^. A model to describe non ideality and synergistic effects in such mixtures will be presented in “Prediction and description of non-ideality, synergistic and antagonistic effects” Section below.

### Mixtures of different water-soluble organics acids

To our knowledge, the surface properties of mixtures of two water-soluble organic acids have not been investigated. Therefore, for completeness, mixtures of glutaric and oxalic acid, two of the most abundant organic compounds in aerosols, were studied in this work. The obtained isotherms are presented in Fig. S21. Plotting the variation of the surface tension of the mixture as function of the molar fraction (Fig. S22) shows that the latter varies linearly, thus that the mixture behaves ideally, with no significant molecular interactions between the two types of acid molecules.

### Prediction and description of non-ideality, synergistic and antagonistic effects

The non-ideal behavior of the surface tension and, in some cases, of the CMC, evidenced in most of the mixtures in this work render the prediction of the surface tension of atmospheric aerosol particles complex. In surfactant science, a widely used model to predict and describe non-ideality and synergistic (or antagonistic) effects in surfactant mixtures is the empirical “molecular interaction parameters” model^46,50^. In this model, the molecular interaction parameters, β^σ^ and β^M^, are introduced to account for the non-ideality on the surface tension and on the CMC, respectively. These two parameters are directly linked to the activity coefficients of the compounds at the surface. Thus, for a binary mixture with compounds 1 and 2:

1 $$ln\left( {f_{1} } \right) = \beta^{\sigma } \times \left( {1 - X_{1} } \right)^{2}$$

2 $$ln\left( {f_{2} } \right) = \beta^{\sigma } \times \left( {X_{1} } \right)^{2}$$

where f_1_ and f_2_ are the activity coefficients of compound 1 and 2, respectively, at the surface and X_1_ the molar fraction of 1 in the binary mixture. The β^M^ parameter is defined in a similar way, based on the activity coefficients in the micelle phase. The β^σ^ and β^M^ parameters represent the excess molecular interaction energy in the mixture compared to the ideal mixture. Thus, negative β values indicate greater attraction between the components, contributing towards synergism, while positive β values indicate greater repulsion, contributing towards antagonism. As suggested by the equations above, β^σ^ should not depend on the molar fraction of the mixture, only on the molar fraction α in the aqueous solution, so that a fixed value of β^σ^ can be attributed to a given mixture. In that case, if values of β^σ^ can be assumed or estimated from the properties of each component of the mixture, this model should be able to predict the non-ideality, synergism or antagonism on the surface tension of given mixtures, and even determine the maximum of the synergistic or antagonistic effect. Reversibly, β^σ^ can also be determined empirically from the adsorption isotherms of mixtures, and its sign can predict or confirm the existence of synergism or antagonism. As an illustration, this was performed in this work for the mixtures Brij35 + glutaric acid, and for two different values of the molar fraction between the two components, X_1_ = 0.13 and X_1_ = 0.0015 (where component 1 here is Brij35). For this, the following formula was applied to the tables S5.8 and S5.10^46^:

3 $$\beta^{\sigma } = \frac{{ln\left( {\alpha C_{12} /X_{1} C_{1}^{o} } \right)}}{{\left( {1 - X_{1} } \right)^{2} }}$$

where α is the molar fraction of Brij35 in the aqueous solution (2nd column in Tables S5.8 and S5.10), C_12_ the concentration of the mixture, and C_1_^o^ the concentration of the solution of Brij35 alone corresponding to the same surface tension than C_12_. The results are presented in Fig. S23, where it can be seen that all the values of β^σ^ obtained are negative, thus confirming the synergistic effects in these mixtures. In addition, Fig. S23 confirms that, beside some uncertainties in the determinations, the values of β^σ^ do not vary with the molar fraction X_1_ between the two components, but only with the molar fraction α in the aqueous solutions. This confirms that this approach can potentially be used to predict non linearity, synergistic and antagonistic effects on the surface tension of such mixtures.

## Conclusion

The results of this work demonstrate that, unlike what might be expected, the presence of inorganic salts and organic acids in aerosols would systematically enhance the efficiency of amphiphilic surfactants rather than reduce it, by lowering the surface tension and, in the case of the salts, also the CMC. In the case of inorganic acids these trends are contrary to the known effects of these compounds, alone, on the surface tension of aqueous solutions. The effects on σ_o_ reported in this work for the different mixtures suggest that the surface tension reduction resulting for these mixtures should be significant in atmospheric aerosol particles and contribute to enhance their transformation into cloud droplets.

Furthermore, the results of this work show that the widely common assumption of ideality for aerosol mixtures is not valid when either inorganic salts or amphiphilic surfactants are present. While non-ideality, synergistic or antagonistic effects in amphiphilic surfactant mixtures are well known and have been extensively studied in surfactant science, this work provides the first experimental evidence for such effects in atmospherically-relevant mixtures. The molecular interactions responsible for these effects depend on the type of mixture. Those evidenced in the mixtures of this work are:

- Electrostatic interactions in the bulk, resulting in the salting out of organic molecules by inorganic ions,
- Weakly ionic or non-ionic interactions (ion–dipole, dipole–dipole, dipole-induced dipole, hydrogen bonding…) between the molecules in the surface mixed monolayer, resulting in the non-linearity and synergistic effects on the surface tension with the organic acids,
- The formation of mixed micelles or competition in micelle formation, resulting in the synergistic or antagonistic effects on the CMC, in the mixtures of different amphiphilic surfactants.

As the main objective of this work was to evidence these effects in atmospherically relevant surfactant mixtures, future investigations would need to examine the surface properties of each type of mixture in more detail. For instance, other inorganic salts than NaCl and (NH_4_)_2_SO_4_ should be studied, and their effects on organic compounds with various number of C-atoms could also be studied. Mixtures of surfactants with other major organic aerosol components than organic acids, such as levoglucosan or other sugars, could also be investigated. Finally, the surface properties of such mixtures could be studied on smaller particles with the new optical tweezer techniques.

## Experimental

Mixtures of amphiphilic surfactants, organic acids and inorganic salts of the compositions indicated in Table S1 were prepared by weighing the compounds on microscales and mixing them with deionized water (18.2 MΩ) in 60 mL glass bottles. These mixtures were then successively diluted in deionized water to obtain the different points of the adsorption isotherms. All the glassware and instruments used were first cleaned following a specific protocol for surfactants^17^. Section S2 provides a complete list of the compounds used with their purities. These compounds were all purchased commercially and used without additional purification.

Surface tension measurements were performed on ~ 500 nL droplets by the pendant drop method using a Dataphysics OCA 15EC tensiometer and Dataphysics SCA software for OCA version 4–4.1. For this, a droplet was generated at the tip of a needle SNP-D 0.91 mm and the surface tension was obtained by comparing its shape to the Young–Laplace Eq. (4)

4 $${\sigma } = { }\frac{{g \Delta \rho \left( {d_{e} } \right)^{2} }}{H}$$

where g is the gravitational acceleration, Δρ is the density difference between the solution and air, d_e_ the main diameter of the droplet, and H a shape factor. The measurements were carried out at 24 (± 2) °C and calibrated with ultrapure water, for which the surface tension obtained was in the range 72.5–73.5 mN m^−1^. Each measurement was repeated three times and the reproducibility between the results was ± (1–3)%. The overall uncertainties on each surface tension measurement were ± (0.3–1.0) mN/m. In the tables provided in the SI, the molar fraction in water (α) has been calculated by taking into consideration the density of the solid compounds.

The values of σ_o_ and of the CMC were obtained graphically from the adsorption isotherms. The value of σ_o_ was read directly as the constant value reached by the isotherm at large concentration. The CMC was obtained by intercepting the horizontal line determined by σ_o_ with a line following the transition zone, as described in previous works^17,18^.

Each surface tension measurement resulted from measurements on 3 individual droplets of the same solution and averaging the results. The main uncertainties in these measurements were thus due to the dispersion between these three droplets. The entire adsorption isotherms for individual compounds (glutaric acid, SDS, CTAC, TritonX100…) were also determined 2 or 3 times. Overall uncertainties of ± 5% on each surface tension measurement were thus applied, as they captured both the dispersion and systematic errors in the measurements. These uncertainties were thus also applied to the values of σ_o_.

### Supplementary Information

Supplementary Information.

## Data Availability

The datasets generated and/or analysed during the current study are available in Supplementary Information.

## References

[1] Lohmann U, Lüönd F, Mahrt F (2016). An Introduction to Clouds: From the Microscale to Climate.

[2] Kohler H (1936). The nucleus in and the growth of hygroscopic droplets. Trans. Faraday Soc..

[3] Chalk, S. J., The IUPAC Gold Book Website. 2019.

[4] Hyvärinen A-P, Lihavainen H, Gaman A, Vairila L, Ojala H, Kulmala M, Viisanen Y (2006). Surface tensions and densities of oxalic, malonic, succinic, maleic, malic, and cis-pinonic acids. J. Chem. Eng. Data.

[5] Svenningsson B, Rissler J, Swietlicki E, Mircea M, Bilde M, Facchini MC, Decesari S, Fuzzi S, Zhou J, Monster J, Rosenorn T (2006). Hygroscopic growth and critical supersaturations for mixed aerosol particles of inorganic and organic compounds of atmospheric relevance. Atmos. Chem. Phys..

[6] Topping DO, McFiggans GB, Kiss G, Varga Z, Facchini MC, Decesari S, Mircea M (2007). Surface tensions of multi-component mixed inorganic/organic aqueous systems of atmospheric significance: Measurements, model predictions and importance for cloud activation predictions. Atmos. Chem. Phys..

[7] Varga Z, Kiss G, Hansson HC (2007). Modelling the cloud condensation nucleus activity of organic acids on the basis of surface tension and osmolality measurements. Atmos. Chem. Phys..

[8] Mahiuddin S, Minofar B, Borah JM, Das MR, Jungwirth P (2008). Propensities of oxalic, citric, succinic, and maleic acids for the aqueous solution/vapour interface: Surface tension measurements and molecular dynamics simulations. Chem. Phys. Lett..

[9] Booth AM, Topping DO, McFiggans G, Percival CJ (2009). Surface tension of mixed inorganic and dicarboxylic acid aqueous solutions at 298.15 K and their importance for cloud activation predictions. Phys. Chem. Chem. Phys..

[10] Aumann E, Hildemann LM, Tabazadeh A (2010). Measuring and modeling the composition and temperature-dependence of surface tension for organic solutions. Atmos. Environ..

[11] Lee JY, Hildemann LM (2014). Surface tensions of solutions containing dicarboxylic acid mixtures. Atmos. Environ..

[12] Duplissy J, Gysel M, Sjogren S, Meyer N, Good N, Kammermann L, Michaud V, Weigel R, Martins dos Santos S, Gruening C, Villani P, Laj P, Sellegri K, Metzger A, McFiggans GB, Wehrle G, Richter R, Dommen J, Ristovski Z, Baltensperger U, Weingartner E (2009). Intercomparison study of six HTDMAs: results and recommendations. Atmos. Meas. Tech..

[13] Latif MT, Brimblecombe P (2004). Surfactants in atmospheric aerosols. Environ. Sci. Technol..

[14] Mustaffa NIH, Latif MT, Ali MM, Khan MF (2014). Source apportionment of surfactants in marine aerosols at different locations along the Malacca Straits. Environ. Sci. Pollut. Res..

[15] Ekström S, Nozière B, Hultberg M, Alsberg T, Magnér J, Nilsson ED, Artaxo P (2010). A possible role of ground-based microorganisms on cloud formation in the atmosphere. Biogeosciences.

[16] Baduel C, Nozière B, Jaffrezo J-L (2012). Summer/winter variability of the surfactants in aerosols from Grenoble, France. Atmos. Environ..

[17] Nozière B, Gérard V, Baduel C, Ferronato C (2017). Extraction and characterization of surfactants from atmospheric aerosols. JoVE (J. Vis. Exp.).

[18] Gérard V, Nozière B, Baduel C, Fine L, Frossard AA, Cohen RC (2016). Anionic, cationic, and nonionic surfactants in atmospheric aerosols from the Baltic coast at Askö, Sweden: Implications for cloud droplet activation. Environ. Sci. Technol..

[19] Gérard V, Noziere B, Fine L, Ferronato C, Singh DK, Frossard AA, Cohen RC, Asmi E, Lihavainen H, Kivekäs N (2019). Concentrations and adsorption isotherms for amphiphilic surfactants in PM1 aerosols from different regions of Europe. Environ. Sci. Technol..

[20] Leko PO, Kozarac Z, Ćosović B (2004). Surface active substances (SAS) and dissolved organic matter (DOC) in atmospheric precipitation of urban area of Croatia (Zagreb). Water Air Soil Pollut..

[21] Orlović-Leko P, Kozarac Z, Ćosović B, Strmečki S, Plavšić M (2010). Characterization of atmospheric surfactants in the bulk precipitation by electrochemical tools. J. Atmos. Chem..

[22] Frka S, Dautovic J, Kozarac Z, Cosovic B, Hoffer A, Kiss G (2012). Surface-active substances in atmospheric aerosol: An electrochemical approach. Tellus Ser. B-Chem. Phys. Meteorol..

[23] Kroflič A, Frka S, Simmel M, Wex H, Grgić I (2018). Size-resolved surface-active substances of atmospheric aerosol: Reconsideration of the impact on cloud droplet formation. Environ. Sci. Technol..

[24] Petters SS, Petters MD (2016). Surfactant effect on cloud condensation nuclei for two-component internally mixed aerosols. J. Geophys. Res.: Atmos..

[25] Lin JJ, Malila J, Prisle NL (2018). Cloud droplet activation of organic–salt mixtures predicted from two model treatments of the droplet surface. Environ. Sci. Process. Impact..

[26] Calderón SM, Malila J, Prisle NL (2020). Model for estimating activity coefficients in binary and ternary ionic surfactant solutions: The CMC based ionic surfactant activity (CISA) model for atmospheric applications. J. Atmos. Chem..

[27] Vepsäläinen, S., Calderón, S. M., & Prisle, N. L. Comparison of six approaches to predicting droplet activation of surface active aerosol–Part 2: strong surfactants. *EGUsphere* 1–23 (2023).

[28] Kleinheins JV, Shardt N, El Haber M, Ferronato C, Nozière B, Peter T, Marcolli C (2023). Surface tension models for binary aqueous solutions: A review and intercomparison. Phys. Chem. Chem. Phys..

[29] Sorjamaa R, Svenningsson B, Raatikainen T, Henning S, Bilde M, Laaksonen A (2004). The role of surfactants in Köhler theory reconsidered. Atmos. Chem. Phys..

[30] Bzdek BR, Power RM, Simpson SH, Reid JP, Royall CP (2016). Precise, contactless measurements of the surface tension of picolitre aerosol droplets. Chem. Sci..

[31] Bzdek BR, Reid JP, Malila J, Prisle NL (2020). The surface tension of surfactant-containing, finite volume droplets. Proc. Natl. Acad. Sci..

[32] Vanhanen J, Hyvärinen AP, Anttila T, Raatikainen T, Viisanen Y, Lihavainen H (2008). Ternary solution of sodium chloride, succinic acid and water; surface tension and its influence on cloud droplet activation. Atmos. Chem. Phys..

[33] Frosch M, Prisle NL, Bilde M, Varga Z, Kiss G (2011). Joint effect of organic acids and inorganic salts on cloud droplet activation. Atmos. Chem. Phys..

[34] Shulman ML, Jacobson MC, Carlson RJ, Synovec RE, Young TE (1996). Dissolution behavior and surface tension effects of organic compounds in nucleating cloud droplets. Geophys. Res. Lett..

[35] Tuckermann R (2007). Surface tension of aqueous solutions of water-soluble organic and inorganic compounds. Atmos. Environ..

[36] Kristensen TB, Prisle NL, Bilde M (2014). Cloud droplet activation of mixed model HULIS and NaCl particles: Experimental results and κ-Köhler theory. Atmos. Res..

[37] Kiss G, Tombacz E, Hansson HC (2005). Surface tension effects of humic-like substances in the aqueous extract of tropospheric fine aerosol. J. Atmos. Chem..

[38] Hansen AMK, Hong J, Raatikainen T, Kristensen K, Ylisirniö A, Virtanen A, Petäjä T, Glasius M, Prisle N (2015). Hygroscopic properties and cloud condensation nuclei activation of limonene-derived organosulfates and their mixtures with ammonium sulfate. Atmos. Chem. Phys..

[39] Helvacı Ş, Peker S, Özdemir G (2004). Effect of electrolytes on the surface behavior of rhamnolipids R1 and R2. Colloids Surfaces B: Biointerfaces.

[40] Yekeen N, Manan MA, Idris AK, Samin AM (2017). Influence of surfactant and electrolyte concentrations on surfactant Adsorption and foaming characteristics. J. Petrol. Sci. Eng..

[41] Xu Q, Nakajima M, Ichikawa S, Nakamura N, Roy P, Okadome H, Shiina T (2009). Effects of surfactant and electrolyte concentrations on bubble formation and stabilization. J. Colloid Interface Sci..

[42] Qazi MJ, Schlegel SJ, Backus EHG, Bonn M, Bonn D, Shahidzadeh N (2020). Dynamic Surface tension of surfactants in the presence of high salt concentrations. Langmuir.

[43] Farajzadeh R, Krastev R, Zitha PLJ (2008). Foam films stabilized with alpha olefin sulfonate (AOS). Colloids Surfaces A-Physicochem. Eng. Aspects.

[44] Majeed T, Solling TI, Kamal MS (2020). Foamstability: The interplay between salt-, surfactant- and critical micelle concentration. J. Petrol. Sci. Eng..

[45] Sharma P, MacNeil JA, Bowles J, Leaist DG (2011). The unusual importance of activity coefficients for micelle solutions illustrated by an osmometry study of aqueous sodium decanoate and aqueous sodium decanoate+ sodium chloride solutions. Phys. Chem. Chem. Phys..

[46] Rosen MJ, Kunjappu JT (2012). Surfactants and Interfacial Phenomena.

[47] Kwaśniewska D, Kiewlicz J (2022). Study of interaction between cationic surfactant (CTAB) and ascorbic acid/ascorbic acids derivatives by tensiometric and spectroscopic methods. J. Mol. Liq..

[48] Kwaśniewska D, Kiewlicz J (2022). Spectroscopic and tensiometric considerations on anionic surfactants (SDS) and ascorbic acid/ascorbates interactions. J. Saudi Chem. Soc..

[49] Tran DN, Prime EL, Plazzer M, Leung AH, Yiapanis G, Christofferson AJ, Yarovsky I, Qiao GG, Solomon DH (2013). Molecular interactions behind the synergistic effect in mixed monolayers of 1-octadecanol and ethylene glycol monooctadecyl ether. J. Phys. Chem. B.

[50] Bagheri A, Khalili P (2017). Synergism between non-ionic and cationic surfactants in a concentration range of mixed monolayers at an air–water interface. RSC Adv..

[51] Shah SK, Chakraborty G, Bhattarai A, De R (2022). Synergistic and antagonistic effects in micellization of mixed surfactants. J. Mol. Liq..

[52] Bera A, Ojha K, Mandal A (2013). Synergistic effect of mixed surfactant systems on foam behavior and surface tension. J. Surfactants Deterg..

[53] Tyagi G, Seddon D, Khodaparast S, Sharratt WN, Robles ESJ, Cabral JT (2021). Tensiometry and FTIR study of the synergy in mixed SDS:DDAO surfactant solutions at varying pH. Colloids Surfaces A-Physicochem. Eng. Aspects.

[54] Sayem Alam M, Ragupathy R, Mandal AB (2016). The self-association and mixed micellization of an anionic surfactant, sodium dodecyl sulfate, and a cationic surfactant, cetyltrimethylammonium bromide: Conductometric, dye solubilization, and surface tension studies. J. Dispers. Sci. Technol..

[55] Zakharova LY, Valeeva FG, Ibragimova AR, Zakharov VM, Kudryavtseva LA, Elistratova YG, Mustafina AR, Konovalov AI, Shtykov SN, Bogomolova IV (2007). Properties of a sodium dodecyl sulfate-Brij 35 binary micellar system and their effect on the alkaline hydrolysis of O-ethyl-O-p-nitrophenylchloromethylphosphonate. Colloid J..

[56] Koneva AS, Ritter E, Anufrikov YA, Lezov AA, Klestova AO, Smirnova NA, Safonova EA, Smirnova I (2018). Mixed aqueous solutions of nonionic surfactants Brij 35/Triton X-100: Micellar properties, solutes’ partitioning from micellar liquid chromatography and modelling with COSMOmic. Colloids Surfaces A: Physicochem. Eng. Aspects.

