# Supplementary material for: Salting out, non-ideality and synergism enhance surfactant efficiency in atmospheric aerosols
Source: Sci Rep. 2023 Nov 24;13:20672. doi: 10.1038/s41598-023-48040-5 (PMC10673862; doi:10.1038/s41598-023-48040-5)
Supplement: Supplementary file 1 — Supplementary Information. [file 41598_2023_48040_MOESM1_ESM.docx]

**Supplementary Information**

**Salting out, non-ideality and Synergism enhance Surfactant Efficiency in Atmospheric Aerosols**

Manuella El haber**^1^**, Corinne Ferronato^1^, Anne Giroir-Fendler,^1^

Ludovic Fine^1^, and Barbara Noziére^2^

*^1^Université Claude Bernard Lyon 1, CNRS, IRCELYON, F-69626, Villeurbanne, France*

*^2^KTH Royal Institute of Technology SE-100 44, Stockholm, Sweden*

Contents

[S1. List of experiments performed 3](#_Toc150421248)

[S2. Experimental work 4](#_Toc150421249)

[S3. Adsorption isotherms for surfactant and inorganic salt mixtures 5](#_Toc150421250)

[**3.1** **TritonX100 + NaCl** 5](#_Toc150421251)

[**3.2** **TritonX100 + (NH_4_)_2_SO_4_** 7](#_Toc150421252)

[**3.3** **Brij35 + (NH_4_)_2_SO_4_** 8](#_Toc150421253)

[**3.4** **Brij35 + NaCl** 10](#_Toc150421254)

[**3.5** **Evolution of the minimal surface tension with the molar fraction of the mixture** 12](#_Toc150421255)

[S4. Adsorption isotherms for organic acid and inorganic salt mixtures 13](#_Toc150421256)

[**4.1** **Glutaric acid + (NH_4_)_2_SO_4_** 13](#_Toc150421257)

[**4.2** **Glutaric acid + NaCl** 14](#_Toc150421258)

[**4.3** **Effects of inorganic salts on the surface tension of glutaric acid** 16](#_Toc150421259)

[S5. Adsorption isotherms for amphiphilic surfactant and organic acid mixtures 17](#_Toc150421260)

[**5.1** **TritonX100 + Glutaric acid** 17](#_Toc150421261)

[**5.2** **Brij35 + Glutaric acid** 21](#_Toc150421262)

[**5.3** **TritonX114 + Glutaric acid** 24](#_Toc150421263)

[**5.4** **TritonX100 + Oxalic acid** 26](#_Toc150421264)

[**5.5** **Brij35 + Oxalic acid** 28](#_Toc150421265)

[**5.6** **CTAC + Oxalic acid** 30](#_Toc150421266)

[**5.7** **SDS + Oxalic acid** 33](#_Toc150421267)

[**5.8** **Effects of organic acids on the CMC of surfactants** 36](#_Toc150421268)

[S6. Adsorption isotherms for mixtures of two amphiphilic surfactants 37](#_Toc150421269)

[**6.1** **SDS + CTAC** 37](#_Toc150421270)

[**6.2** **TritonX114 + SDS** 38](#_Toc150421271)

[S7. Adsorption isotherms for two organic acids mixtures 42](#_Toc150421272)

[**7.1** **Glutaric acid + Oxalic acid** 42](#_Toc150421273)

[**7.2** **Evolution of the surface tension for Glutaric and Oxalic acid mixtures** 43](#_Toc150421274)

[S8. Determination of the β^σ^ parameter for the mixtures Brij 35 + glutaric acid 44](#_Toc150421275)

# **S1. List of experiments performed**

**Table S1: List of experiments performed**

| **Mixture. n.** | **Compound 1** | **Compound 2** | **Initial concentrations** |
| --- | --- | --- | --- |
| **Amphiphilic surfactant + inorganic salt** | | | |
| **1** | TritonX100 | NaCl | TritonX100 0.1 M + 0, 0.018, 0.4, 0.8, 1.6, 2.8 M NaCl |
| **2** | TritonX100 | (NH_4_)_2_SO_4_ | TritonX100 0.1 M + 0, 0.0054, 0.41, 0.7 M (NH_4_)_2_SO_4_ |
| **3** | Brij35 | (NH_4_)_2_SO_4_ | Brij35 0.02 M + 0, 0.0034, 0.41, 0.7 M (NH_4_)_2_SO_4_ |
| **4** | Brij35 | NaCl | Brij35 0.02 M + 0, 0.02, 0.4, 0.8, 1.6 M NaCl |
| **Organic acid + inorganic salt** | | | |
| **5** | Glutaric acid | (NH_4_)_2_SO_4_ | Glutaric acid 3 M + 0, 0.7, 1.5 M (NH_4_)_2_SO_4_ |
| **6** | Glutaric acid | NaCl | Glutaric acid 3 M + 0, 0.4, 1.7, 2.5 M NaCl |
| **Amphiphilic surfactant + organic acid** | | | |
| **7** | TritonX100 | Glutaric acid | TritonX100 0.1 M + x = 0, 0.87, 0.984, 0.99833, 0.99985, 1 GA* |
| **8** | TritonX100 | Glutaric acid | TritonX100 0.1 M + 0, 0.0065, 0.65, 2.7 M GA* |
| **9** | Brij35 | Glutaric acid | Brij35 0.02 M + x = 0, 0.87, 0.986, 0.9985, 0.99986, 1 GA* |
| **10** | Brij35 | Glutaric acid | Brij35 0.02 M + 0, 0.65, 02 M GA* |
| **11** | TritonX114 | Glutaric acid | TritonX114 0.01 M + 0, 0.6, 2 M GA* |
| **12** | TritonX100 | Oxalic acid | TritonX100 0.1 M + x = 0, 0.9, 0.99, 0.9989, 0.99953, 0.99987, 1 OA** |
| **13** | Brij35 | Oxalic acid | Brij35 0.02 M + x = 0, 0.909, 0.9901, 0.998899, 0.99982, 1 OA** |
| **14** | CTAC | Oxalic acid | CTAC 0.1 M + x = 0, 0.91, 0.99, 0.9986, 0.9999, 1 OA** |
| **15** | SDS | Oxalic acid | SDS 0.1 M + x = 0, 0.91, 0.99, 0.999, 0.9999, 1 OA** |
| **Two different amphiphilic surfactants** | | | |
| **16** | SDS | CTAC | SDS 0.1 M + x = 0, 0.33, 0.5, 0.66, 1 CTAC |
| **17** | TritonX114 | SDS | TritonX114 0.01 M + x = 0. 0.1, 0.33, 0.5, 0.66, 1 SDS |
| **Two different organic acids** | | | |
| **18** | Oxalic acid | Glutaric acid | Oxalic acid 1 M + x = 0, 0.25, 0.4, 0.57, 1 GA* |

*GA= glutaric acid; **OA= oxalic acid

# **S2. Experimental work**

Principle binary solutions of surfactants, acids and salts listed in Table S2 of this Supplementary Information, were prepared in 60 mL glass bottles with deionized water (18.2 MΩ). In order to ensure its homogeneity, all solutions were placed on a magnetic stirrer for an hour. Afterwards, the ternary mixtures listed in Table S1 were prepared in deionized water for tensiometry and their surface tension was measured by the pendant drop method using a Dataphysics OCA 15EC tensiometer and Dataphysics SCA software for OCA version 4 - 4.1. The tensiometer was calibrated with ultrapure water and the measurements were carried out at 24 (± 2) °C; the SFT of pure water was in the range of 72.5–73.5 mN/m. Each measurement was repeated three times, and the reproducibility between the results was ± (1 - 3) %. The overall uncertainties on each surface tension measurement were ± (0.3 - 1.0) mN/m.

**Table S2: List of the compounds used in this study**

| Compound | Formula | MW (g/mol) | CAS no. | Purity (%) |
| --- | --- | --- | --- | --- |
| TritonX100 | C_14_H_22_O(C_2_H_4_O)_n_ | 647 | 9036-19-5 | 100 |
| TritonX114 | C_14_H_22_O(C_2_H_4_O)_n_ | 537 | 9036-19-5 | 100 |
| Brij35 | C_12_H_26_O(C_2_H_4_O)_n_ | 1199.54 | 9002-92-0 | 100 |
| CetylTrimethyl Ammonium Chloride, “CTAC” | C_19_H_42_NCl | 320 | 112-02-7 | 25 |
| Sodium Dodecyl Sulfate, “SDS” | C₁₂H₂₅OSO₂ONa | 288.37 | 151-21-3 | ≥ 99 |
| Ethanedioic acid, “oxalic acid” | C_2_H_2_O_4_ | 90.03 | 144-62-7 | 100 |
| Pentanedioic acid, “glutaric acid” | C_5_H_8_O_4_ | 132.12 | 110-94-1 | 100 |
| Sodium chloride | NaCl | 58.44 | 7647-14-5 | ≥ 99.5 |
| Ammonium sulfate | (NH₄)₂SO₄ | 132.14 | 7783-20-2 | ≥ 99 |

# **S3. Adsorption isotherms for** **surfactant and inorganic salt mixtures**

*With α = molar fraction of compounds in water.*

### **TritonX100 + NaCl**

**Table S3.1: TritonX100 solutions**

| [TritonX100] (M) | σ (mN/m) | [TritonX100] (M) | σ (mN/m) |
| --- | --- | --- | --- |
| 9.7E-03 | 32.37 | 1.6E-04 | 37.55 |
| 5.0E-03 | 32.38 | 1.1E-04 | 41.38 |
| 2.1E-03 | 31.75 | 5.5E-05 | 47.40 |
| 1.0E-03 | 31.80 | 4.9E-05 | 48.83 |
| 6.9E-04 | 31.90 | 4.4E-05 | 50.10 |
| 5.1E-04 | 31.52 | 3.3E-05 | 53.33 |
| 3.3E-04 | 32.60 | 8.0E-06 | 71.44 |
| 2.6E-04 | 33.67 | 2.9E-06 | 73.10 |
| 2.2E-04 | 36.40 | 0.0E+00 | 73.53 |

**Table S3.2: TritonX100 + 0.018 M NaCl mixtures**

| ∝ TritonX100 | ∝ NaCl | [TritonX100] (M) | [NaCl] (M) | σ (mN/m) |
| --- | --- | --- | --- | --- |
| 1.59E-05 | 3.26E-04 | 8.8E-04 | 1.8E-02 | 31.65 |
| 1.27E-05 | 3.27E-04 | 7.1E-04 | 1.8E-02 | 31.76 |
| 7.78E-06 | 3.25E-04 | 4.3E-04 | 1.8E-02 | 31.23 |
| 6.57E-06 | 3.22E-04 | 3.7E-04 | 1.8E-02 | 31.65 |
| 4.80E-06 | 3.25E-04 | 2.7E-04 | 1.8E-02 | 33.01 |
| 3.22E-06 | 3.29E-04 | 1.8E-04 | 1.8E-02 | 36.22 |
| 1.68E-06 | 3.27E-04 | 9.4E-05 | 1.8E-02 | 42.31 |
| 1.37E-06 | 3.27E-04 | 7.6E-05 | 1.8E-02 | 44.26 |
| 8.61E-07 | 3.16E-04 | 4.8E-05 | 1.8E-02 | 47.57 |
| 8.02E-07 | 3.31E-04 | 4.5E-05 | 1.8E-02 | 50.28 |
| 2.91E-07 | 3.24E-04 | 1.6E-05 | 1.8E-02 | 61.64 |
| 1.57E-07 | 3.19E-04 | 8.7E-06 | 1.8E-02 | 67.35 |
| 8.48E-08 | 3.15E-04 | 4.7E-06 | 1.7E-02 | 70.91 |
| 0.00E+00 | 3.24E-04 | 0.0E+00 | 1.8E-02 | 73.20 |

**Table S3.3: TritonX100 + 0.4 M NaCl mixtures**

| ∝ TritonX100 | ∝ NaCl | [TritonX100] (M) | [NaCl] (M) | σ (mN/m) |
| --- | --- | --- | --- | --- |
| 1.55E-05 | 8.62E-03 | 8.6E-04 | 4.8E-01 | 31.04 |
| 1.27E-05 | 7.28E-03 | 7.0E-04 | 4.0E-01 | 31.05 |
| 7.89E-06 | 7.30E-03 | 4.4E-04 | 4.0E-01 | 31.12 |
| 6.43E-06 | 6.91E-03 | 3.6E-04 | 3.8E-01 | 30.95 |
| 4.78E-06 | 6.84E-03 | 2.6E-04 | 3.8E-01 | 30.97 |
| 3.17E-06 | 6.89E-03 | 1.8E-04 | 3.8E-01 | 33.31 |
| 1.62E-06 | 6.99E-03 | 9.0E-05 | 3.9E-01 | 39.77 |
| 1.35E-06 | 6.89E-03 | 7.5E-05 | 3.8E-01 | 41.88 |
| 8.47E-07 | 7.24E-03 | 4.7E-05 | 4.0E-01 | 46.58 |
| 6.95E-07 | 6.84E-03 | 3.8E-05 | 3.8E-01 | 48.63 |
| 2.35E-07 | 6.67E-03 | 1.3E-05 | 3.7E-01 | 60.30 |
| 2.13E-07 | 7.32E-03 | 1.2E-05 | 4.1E-01 | 65.68 |
| 1.07E-07 | 6.98E-03 | 5.9E-06 | 3.9E-01 | 71.97 |
| 0.00E+00 | 6.93E-03 | 0.0E+00 | 3.8E-01 | 72.08 |

**Table S3.4: TritonX100 + 0.8 M NaCl mixtures**

| ∝ TritonX100 | ∝ NaCl | [TritonX100] (M) | [NaCl] (M) | σ (mN/m) |
| --- | --- | --- | --- | --- |
| 1.64E-05 | 1.41E-02 | 9.1E-04 | 7.8E-01 | 30.53 |
| 8.27E-06 | 1.39E-02 | 4.6E-04 | 7.7E-01 | 30.50 |
| 5.32E-06 | 1.41E-02 | 2.9E-04 | 7.8E-01 | 30.54 |
| 3.29E-06 | 1.42E-02 | 1.8E-04 | 7.8E-01 | 30.65 |
| 1.70E-06 | 1.39E-02 | 9.4E-05 | 7.7E-01 | 36.18 |
| 1.31E-06 | 1.40E-02 | 7.3E-05 | 7.7E-01 | 38.20 |
| 8.44E-07 | 1.40E-02 | 4.7E-05 | 7.7E-01 | 42.89 |
| 5.12E-07 | 1.41E-02 | 2.8E-05 | 7.8E-01 | 47.16 |
| 1.79E-07 | 1.40E-02 | 9.9E-06 | 7.7E-01 | 60.92 |
| 1.27E-07 | 1.44E-02 | 7.0E-06 | 8.0E-01 | 70.02 |
| 1.00E-07 | 1.37E-02 | 5.5E-06 | 7.5E-01 | 69.96 |
| 7.87E-08 | 1.34E-02 | 4.3E-06 | 7.4E-01 | 70.50 |
| 0.00E+00 | 1.40E-02 | 0.0E+00 | 7.7E-01 | 71.91 |

**Table S3.5**: **TritonX100 + 1.6 M NaCl mixtures**

| ∝ TritonX100 | ∝ NaCl | [TritonX100] (M) | [NaCl] (M) | σ (mN/m) |
| --- | --- | --- | --- | --- |
| 1.68E-05 | 2.94E-02 | 9.2E-04 | 1.6E+00 | 29.46 |
| 8.57E-06 | 2.97E-02 | 4.7E-04 | 1.6E+00 | 29.49 |
| 5.15E-06 | 2.98E-02 | 2.8E-04 | 1.6E+00 | 29.60 |
| 3.28E-06 | 2.91E-02 | 1.8E-04 | 1.6E+00 | 29.38 |
| 1.83E-06 | 2.92E-02 | 1.0E-04 | 1.6E+00 | 31.00 |
| 1.42E-06 | 2.91E-02 | 7.8E-05 | 1.6E+00 | 33.18 |
| 9.14E-07 | 2.91E-02 | 5.0E-05 | 1.6E+00 | 37.44 |
| 3.97E-07 | 2.87E-02 | 2.2E-05 | 1.6E+00 | 46.45 |
| 2.27E-07 | 2.97E-02 | 1.3E-05 | 1.6E+00 | 57.81 |
| 1.86E-07 | 2.95E-02 | 1.0E-05 | 1.6E+00 | 61.05 |
| 1.56E-07 | 3.03E-02 | 8.6E-06 | 1.7E+00 | 69.65 |
| 1.35E-07 | 3.05E-02 | 7.4E-06 | 1.7E+00 | 70.89 |
| 0.00E+00 | 2.93E-02 | 0.0E+00 | 1.6E+00 | 70.97 |

**Table S3.6**: **TritonX100 + 2.8 M NaCl mixtures**

| ∝ TritonX100 | ∝ NaCl | [TritonX100] (M) | [NaCl] (M) | σ (mN/m) |
| --- | --- | --- | --- | --- |
| 1.77E-05 | 5.26E-02 | 9.6E-04 | 2.8E+00 | 27.76 |
| 8.73E-06 | 5.29E-02 | 4.7E-04 | 2.9E+00 | 28.02 |
| 5.38E-06 | 5.27E-02 | 2.9E-04 | 2.9E+00 | 28.04 |
| 3.49E-06 | 5.30E-02 | 1.9E-04 | 2.9E+00 | 27.73 |
| 1.74E-06 | 5.29E-02 | 9.4E-05 | 2.9E+00 | 28.77 |
| 1.45E-06 | 5.28E-02 | 7.9E-05 | 2.9E+00 | 29.46 |
| 9.04E-07 | 5.27E-02 | 4.9E-05 | 2.9E+00 | 39.8 |
| 4.46E-07 | 5.29E-02 | 2.4E-05 | 2.9E+00 | 46.86 |
| 2.94E-07 | 5.24E-02 | 1.6E-05 | 2.8E+00 | 51.24 |
| 2.82E-07 | 4.77E-02 | 1.5E-05 | 2.6E+00 | 54.88 |
| 0.00E+00 | 5.16E-02 | 0.0E+00 | 2.8E+00 | 69.62 |


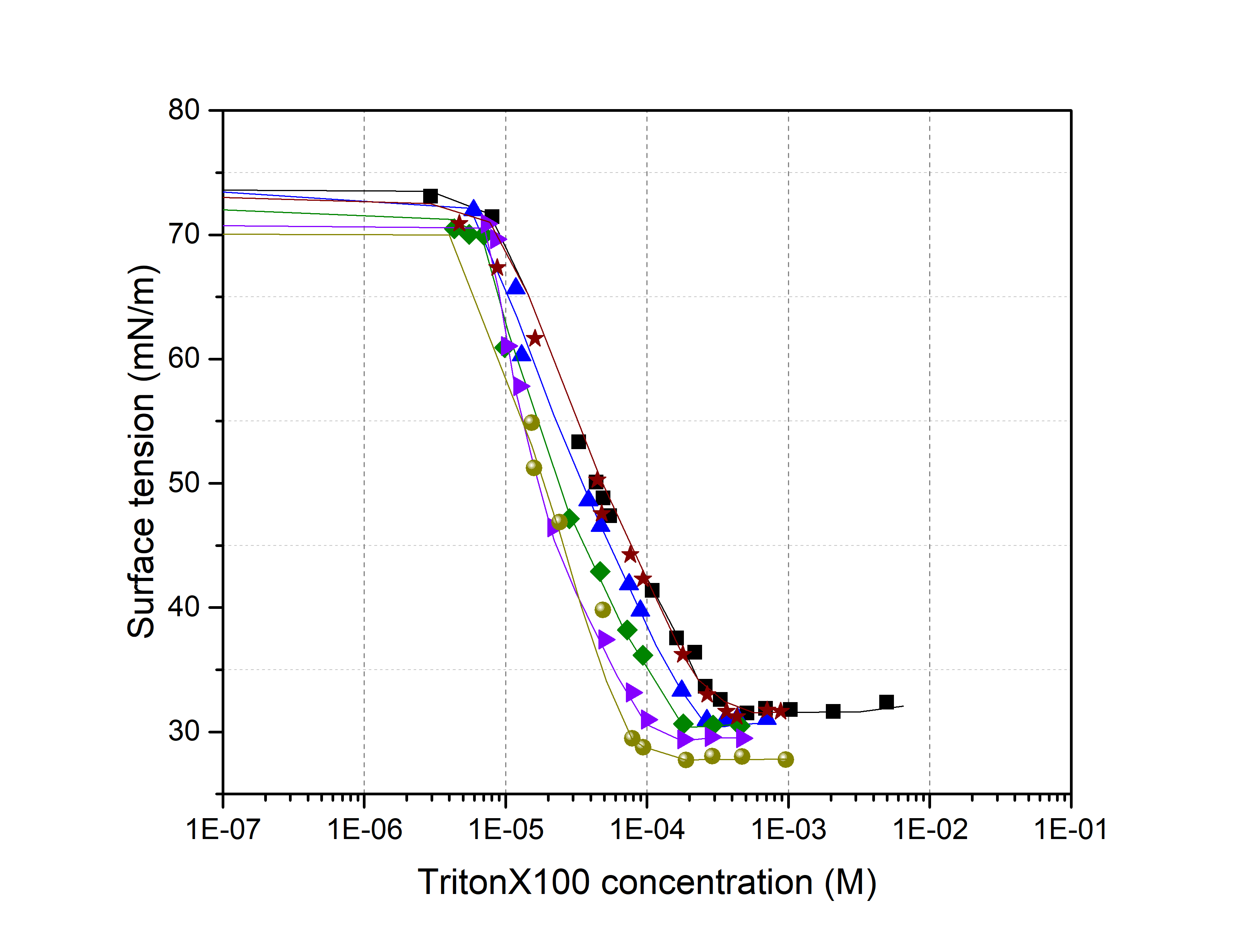


**Figure S1**: **Adsorption isotherms for TritonX100 + NaCl mixtures**: (
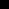
) black curve: TritonX100; (
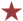
) wine curve: TritonX100 + 0.018 M NaCl; (
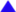
) blue curve: TritonX100 + 0.4 M NaCl; (
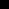
) green curve: TritonX100 + 0.8 M NaCl; (
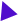
) violet curve: TritonX100 + 1.6 M NaCl and (
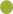
) yellow curve: TritonX100 + 2.8 M NaCl.

### **TritonX100 + (NH_4_)_2_SO_4_**

**Table S3.7: TritonX100 + 0.0054 M (NH_4_)_2_SO_4_ mixtures**

| ∝ TritonX100 | ∝ AS | [TritonX100] (M) | [AS] (M) | σ (mN/m) |
| --- | --- | --- | --- | --- |
| 1.86E-05 | 9.76E-05 | 1.0E-03 | 5.4E-03 | 31.71 |
| 1.13E-05 | 9.81E-05 | 6.3E-04 | 5.4E-03 | 31.89 |
| 6.53E-06 | 9.80E-05 | 3.6E-04 | 5.4E-03 | 31.64 |
| 4.71E-06 | 9.76E-05 | 2.6E-04 | 5.4E-03 | 33.00 |
| 2.28E-06 | 9.71E-05 | 1.3E-04 | 5.4E-03 | 39.86 |
| 1.75E-06 | 9.74E-05 | 9.7E-05 | 5.4E-03 | 41.30 |
| 1.14E-06 | 9.81E-05 | 6.3E-05 | 5.4E-03 | 47.33 |
| 1.05E-06 | 9.78E-05 | 5.8E-05 | 5.4E-03 | 46.98 |
| 5.90E-07 | 9.61E-05 | 3.3E-05 | 5.3E-03 | 51.23 |
| 3.51E-07 | 9.73E-05 | 2.0E-05 | 5.4E-03 | 58.77 |
| 1.24E-07 | 9.60E-05 | 6.9E-06 | 5.3E-03 | 69.67 |
| 6.41E-08 | 9.61E-05 | 3.6E-06 | 5.3E-03 | 72.00 |
| 0.00E+00 | 9.67E-05 | 0.0E+00 | 5.4E-03 | 73.19 |

**Table S3.8: TritonX100 + 0.41 M (NH_4_)_2_SO_4_ mixtures**

| ∝ TritonX100 | ∝ AS | [TritonX100] (M) | [AS] (M) | σ (mN/m) |
| --- | --- | --- | --- | --- |
| 1.80E-05 | 7.72E-03 | 9.8E-01 | 4.2E-01 | 30.75 |
| 1.09E-05 | 7.62E-03 | 5.9E-01 | 4.1E-01 | 30.83 |
| 5.39E-06 | 7.63E-03 | 2.9E-01 | 4.1E-01 | 31.03 |
| 2.70E-06 | 7.56E-03 | 1.5E-01 | 4.1E-01 | 32.04 |
| 1.13E-06 | 7.60E-03 | 6.1E-02 | 4.1E-01 | 41.00 |
| 7.38E-07 | 7.62E-03 | 4.0E-02 | 4.1E-01 | 45.03 |
| 3.54E-07 | 7.64E-03 | 1.9E-02 | 4.1E-01 | 59.92 |
| 1.72E-07 | 7.69E-03 | 9.3E-03 | 4.2E-01 | 68.60 |
| 1.01E-07 | 7.67E-03 | 5.5E-03 | 4.2E-01 | 71.83 |
| 4.28E-08 | 7.55E-03 | 2.3E-03 | 4.1E-01 | 72.50 |
| 0.00E+00 | 7.56E-03 | 0.0E+00 | 4.1E-01 | 72.13 |

**Table S3.9: TritonX100 + 0.7 M (NH_4_)_2_SO_4_ mixtures**

| ∝ TritonX100 | ∝ AS | [TritonX100] (M) | [AS] (M) | σ (mN/m) |
| --- | --- | --- | --- | --- |
| 1.90E-05 | 1.24E-02 | 1.0E-03 | 6.6E-01 | 29.88 |
| 9.92E-06 | 1.25E-02 | 5.3E-04 | 6.7E-01 | 30.05 |
| 5.75E-06 | 1.27E-02 | 3.1E-04 | 6.8E-01 | 30.08 |
| 4.06E-06 | 1.23E-02 | 2.2E-04 | 6.6E-01 | 30.31 |
| 1.93E-06 | 1.24E-02 | 1.0E-04 | 6.6E-01 | 31.24 |
| 1.46E-06 | 1.28E-02 | 7.8E-05 | 6.8E-01 | 33.96 |
| 1.01E-06 | 1.24E-02 | 5.4E-05 | 6.6E-01 | 38.29 |
| 5.86E-07 | 1.25E-02 | 3.1E-05 | 6.7E-01 | 46.25 |
| 3.94E-07 | 1.25E-02 | 2.1E-05 | 6.7E-01 | 49.55 |
| 1.19E-07 | 1.26E-02 | 6.4E-06 | 6.7E-01 | 69.85 |
| 5.42E-08 | 1.26E-02 | 2.9E-06 | 6.8E-01 | 71.38 |
| 0.00E+00 | 1.22E-02 | 0.0E+00 | 6.5E-01 | 71.75 |


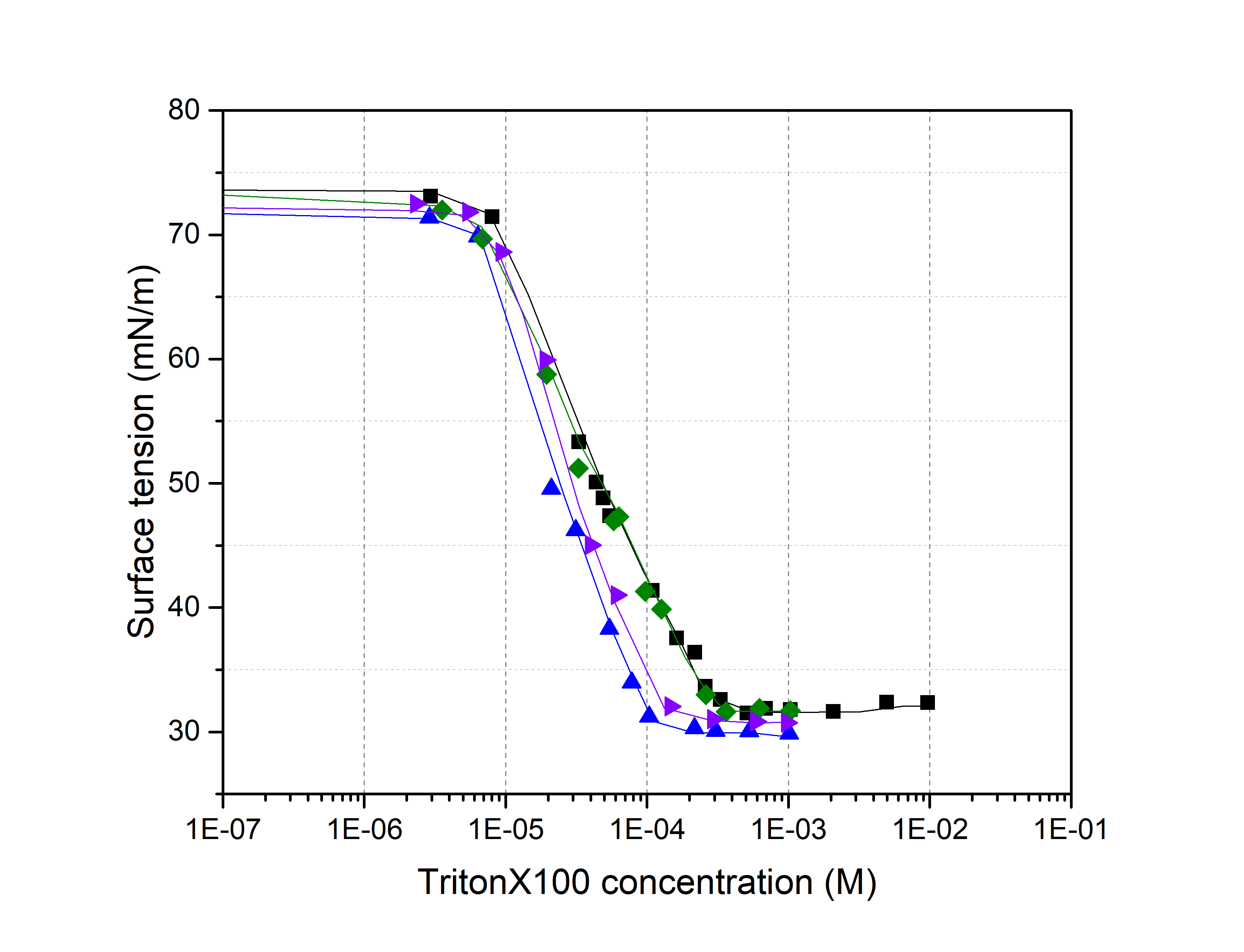


**Figure S2**: **Adsorption isotherms for TritonX100 + (NH_4_)_2_SO_4_ mixtures**: (
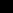
) black curve: TritonX100; (
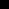
) green curve: TritonX100 + 0.0054 M (NH_4_)_2_SO_4_; (
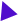
) violet curve: TritonX100 + 0.41 M (NH_4_)_2_SO_4_ and (
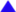
) blue curve: TritonX100 + 0.7 M (NH_4_)_2_SO_4_.

### **Brij35 +** **(NH_4_)_2_SO_4_**

**Table S3.10: Brij35 solutions**

| [Brij35] (M) | σ (mN/m) | [Brij35] (M) | σ (mN/m) |
| --- | --- | --- | --- |
| 2.06E-02 | 44.91 | 1.08E-04 | 47.80 |
| 1.04E-02 | 45.30 | 8.62E-05 | 49.35 |
| 7.76E-03 | 45.22 | 6.41E-05 | 50.40 |
| 4.95E-03 | 45.13 | 4.59E-05 | 53.32 |
| 1.03E-03 | 45.34 | 3.43E-05 | 53.73 |
| 7.58E-04 | 44.87 | 2.44E-05 | 56.79 |
| 5.59E-04 | 45.42 | 5.23E-06 | 71.88 |
| 3.85E-04 | 45.66 | 0.00E+00 | 73.30 |
| 2.00E-04 | 45.66 |  |  |

**Table S3.11: Brij35 + 0.0034 M (NH_4_)_2_SO_4_ mixtures**

| ∝ Brij35 | ∝ AS | [Brij35] (M) | [AS] (M) | σ (mN/m) |
| --- | --- | --- | --- | --- |
| 1.68E-04 | 5.90E-05 | 9.32E-03 | 3.3E-03 | 45.35 |
| 9.47E-05 | 6.04E-05 | 5.3E-03 | 3.4E-03 | 45.24 |
| 1.84E-05 | 6.00E-05 | 1.0E-03 | 3.3E-03 | 45.76 |
| 9.75E-06 | 6.04E-05 | 5.4E-04 | 3.4E-03 | 44.85 |
| 7.28E-06 | 6.00E-05 | 4.0E-04 | 3.3E-03 | 45.18 |
| 5.25E-06 | 5.93E-05 | 2.9E-04 | 3.3E-03 | 45.42 |
| 2.02E-06 | 6.01E-05 | 1.1E-04 | 3.3E-03 | 47.18 |
| 8.55E-07 | 6.06E-05 | 4.7E-05 | 3.4E-03 | 52.04 |
| 5.14E-07 | 6.03E-05 | 2.9E-05 | 3.3E-03 | 54.76 |
| 2.57E-07 | 6.05E-05 | 1.4E-05 | 3.4E-03 | 58.13 |
| 9.06E-08 | 6.00E-05 | 5.0E-06 | 3.3E-03 | 68.00 |
| 4.57E-08 | 6.01E-05 | 2.5E-06 | 3.3E-03 | 71.35 |
| 0.00E+00 | 5.98E-05 | 0.0E+00 | 3.3E-03 | 72.96 |

**Table S3.12: Brij35 + 0.41 M (NH_4_)_2_SO_4_ mixtures**

| ∝ Brij35 | ∝ AS | [Brij35] (M) | [AS] (M) | σ (mN/m) |
| --- | --- | --- | --- | --- |
| 1.76E-04 | 7.70E-03 | 9.6E-03 | 4.2E-01 | 43.62 |
| 9.20E-05 | 7.67E-03 | 5.0E-03 | 4.2E-01 | 42.59 |
| 1.85E-05 | 7.61E-03 | 1.0E-03 | 4.1E-01 | 43.40 |
| 1.06E-05 | 7.59E-03 | 5.7E-04 | 4.1E-01 | 42.80 |
| 7.57E-06 | 7.64E-03 | 4.1E-04 | 4.1E-01 | 43.17 |
| 4.77E-06 | 7.60E-03 | 2.6E-04 | 4.1E-01 | 43.15 |
| 1.85E-06 | 7.67E-03 | 1.0E-04 | 4.2E-01 | 44.65 |
| 1.16E-06 | 7.66E-03 | 6.3E-05 | 4.2E-01 | 46.01 |
| 6.88E-07 | 7.68E-03 | 3.7E-05 | 4.2E-01 | 49.17 |
| 2.52E-07 | 7.63E-03 | 1.4E-05 | 4.1E-01 | 57.29 |
| 1.48E-07 | 7.65E-03 | 8.0E-06 | 4.1E-01 | 62.68 |
| 7.42E-08 | 7.66E-03 | 4.0E-06 | 4.2E-01 | 69.15 |
| 0.00E+00 | 7.67E-03 | 0.0E+00 | 4.2E-01 | 72.45 |

**Table S3.13: Brij35 + 0.7 M (NH_4_)_2_SO_4_ mixtures**

| ∝ Brij35 | ∝ AS | [Brij35] (M) | [AS] (M) | σ (mN/m) |
| --- | --- | --- | --- | --- |
| 1.84E-04 | 1.19E-02 | 9.9E-03 | 6.4E-01 | 40.89 |
| 9.44E-05 | 1.29E-02 | 5.0E-03 | 6.9E-01 | 40.81 |
| 1.88E-05 | 1.22E-02 | 1.0E-03 | 6.5E-01 | 40.94 |
| 1.53E-05 | 1.21E-02 | 8.2E-04 | 6.5E-01 | 40.99 |
| 1.15E-05 | 1.20E-02 | 6.2E-04 | 6.4E-01 | 41.45 |
| 7.83E-06 | 1.21E-02 | 4.2E-04 | 6.5E-01 | 41.42 |
| 3.86E-06 | 1.23E-02 | 2.1E-04 | 6.6E-01 | 41.85 |
| 1.98E-06 | 1.23E-02 | 1.1E-04 | 6.6E-01 | 42.01 |
| 1.52E-06 | 1.23E-02 | 8.1E-05 | 6.6E-01 | 42.68 |
| 1.08E-06 | 1.23E-02 | 5.8E-05 | 6.6E-01 | 43.47 |
| 8.58E-07 | 1.23E-02 | 4.6E-05 | 6.6E-01 | 44.00 |
| 6.26E-07 | 1.25E-02 | 3.4E-05 | 6.7E-01 | 45.49 |
| 4.78E-07 | 1.22E-02 | 2.6E-05 | 6.5E-01 | 49.29 |
| 2.78E-07 | 1.21E-02 | 1.5E-05 | 6.5E-01 | 54.13 |
| 1.92E-07 | 1.23E-02 | 1.0E-05 | 6.6E-01 | 59.85 |
| 1.01E-07 | 1.24E-02 | 5.4E-06 | 6.6E-01 | 65.48 |
| 0.00E+00 | 1.21E-02 | 0.0E+00 | 6.5E-01 | 71.51 |


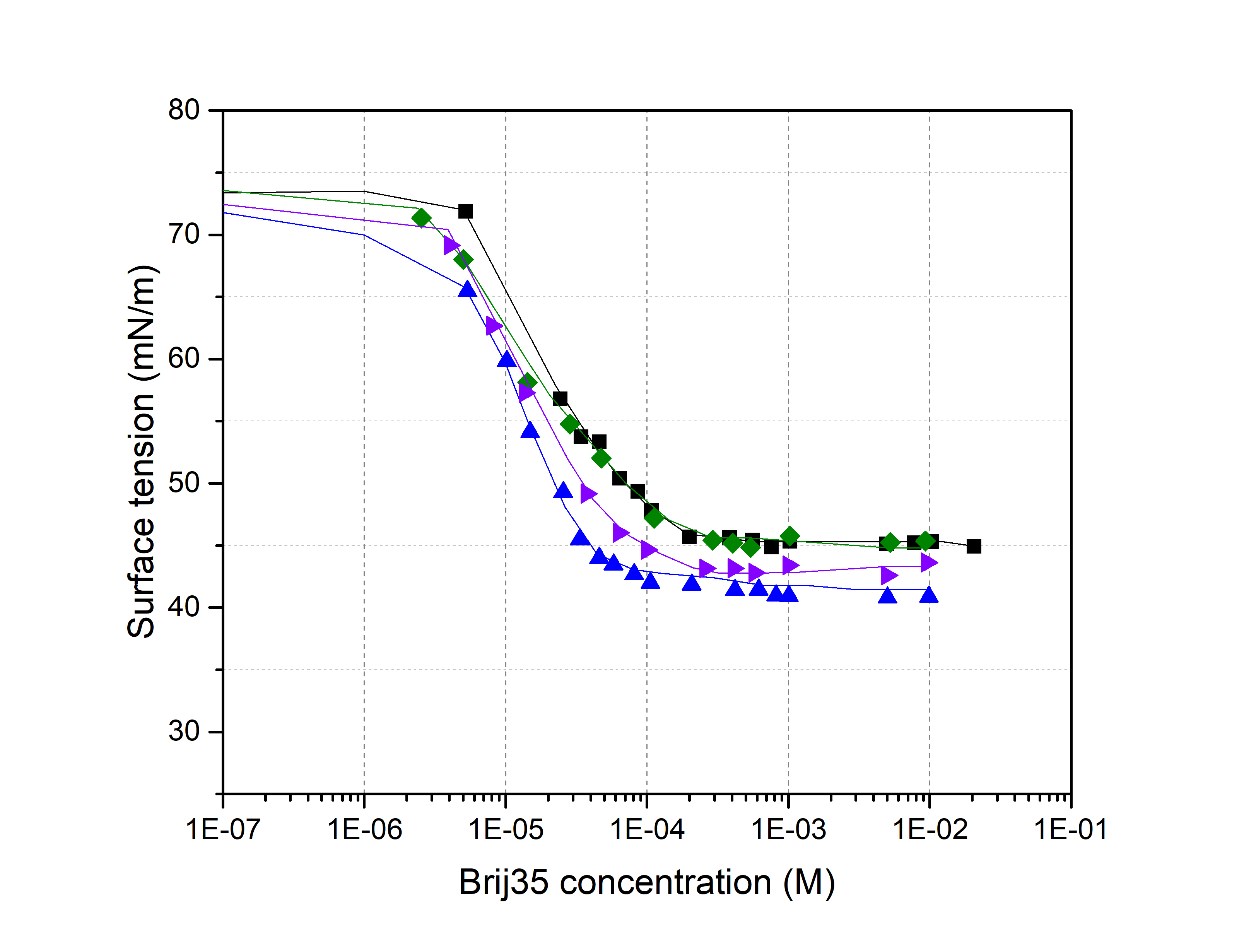


**Figure S3: Adsorption isotherms for Brij35 + (NH_4_)_2_SO_4_ mixtures:** (
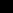
) black curve: Brij35; (
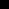
) green curve: Brij35 + 0.0034 M (NH_4_)_2_SO_4_; (
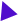
) violet curve: Brij35 + 0.41 M (NH_4_)_2_SO_4_ and (
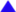
) blue curve: Brij35 + 0.7 M (NH_4_)_2_SO_4_.

### **Brij35 + NaCl**

**Table S3.14: Brij35 + 0.021 M NaCl mixtures**

| ∝ Brij35 | ∝ NaCl | [Brij35] (M) | [NaCl] (M) | σ (mN/m) |
| --- | --- | --- | --- | --- |
| 3.58E-05 | 3.78E-04 | 2.0E-03 | 2.1E-02 | 44.82 |
| 2.64E-05 | 3.79E-04 | 1.5E-03 | 2.1E-02 | 44.76 |
| 1.63E-05 | 3.76E-04 | 9.0E-04 | 2.1E-02 | 44.63 |
| 1.41E-05 | 3.78E-04 | 7.8E-04 | 2.1E-02 | 44.95 |
| 9.93E-06 | 3.84E-04 | 5.5E-04 | 2.1E-02 | 44.63 |
| 5.58E-06 | 3.86E-04 | 3.1E-04 | 2.1E-02 | 44.50 |
| 1.99E-06 | 3.69E-04 | 1.1E-04 | 2.1E-02 | 46.92 |
| 1.42E-06 | 3.73E-04 | 7.9E-05 | 2.1E-02 | 48.07 |
| 1.05E-06 | 3.71E-04 | 5.9E-05 | 2.1E-02 | 48.97 |
| 4.55E-07 | 3.72E-04 | 2.5E-05 | 2.1E-02 | 53.55 |
| 1.97E-07 | 3.72E-04 | 1.1E-05 | 2.1E-02 | 58.20 |
| 1.00E-07 | 3.72E-04 | 5.6E-06 | 2.1E-02 | 68.90 |
| 0.00E+00 | 3.72E-04 | 0.0E+00 | 2.1E-02 | 72.72 |

**Table S3.15: Brij35 + 0.4 M NaCl mixtures**

| ∝ Brij35 | ∝ NaCl | [Brij35] (M) | [NaCl] (M) | σ (mN/m) |
| --- | --- | --- | --- | --- |
| 3.48E-05 | 7.18E-03 | 1.9E-03 | 4.0E-01 | 43.77 |
| 2.73E-05 | 7.21E-03 | 1.5E-03 | 4.0E-01 | 43.52 |
| 1.62E-05 | 7.22E-03 | 9.0E-04 | 4.0E-01 | 43.87 |
| 8.96E-06 | 7.30E-03 | 5.0E-04 | 4.0E-01 | 43.61 |
| 3.43E-06 | 7.16E-03 | 1.9E-04 | 4.0E-01 | 43.60 |
| 2.74E-06 | 7.16E-03 | 1.5E-04 | 4.0E-01 | 44.03 |
| 1.81E-06 | 7.15E-03 | 1.0E-04 | 4.0E-01 | 45.71 |
| 9.45E-07 | 7.18E-03 | 5.2E-05 | 4.0E-01 | 48.00 |
| 7.55E-07 | 7.17E-03 | 4.2E-05 | 4.0E-01 | 49.91 |
| 2.46E-07 | 7.17E-03 | 1.4E-05 | 4.0E-01 | 59.00 |
| 5.64E-08 | 7.14E-03 | 3.1E-06 | 4.0E-01 | 67.02 |
| 0.00E+00 | 7.19E-03 | 0.0E+00 | 4.0E-01 | 72.75 |

**Table S3.16: Brij35 + 0.8 M NaCl mixtures**

| ∝ Brij35 | ∝ NaCl | [Brij35] (M) | [NaCl] (M) | σ (mN/m) |
| --- | --- | --- | --- | --- |
| 3.41E-05 | 1.46E-02 | 1.9E-03 | 8.1E-01 | 42.55 |
| 2.61E-05 | 1.47E-02 | 1.4E-03 | 8.1E-01 | 42.39 |
| 1.61E-05 | 1.46E-02 | 8.9E-04 | 8.0E-01 | 42.62 |
| 1.03E-05 | 1.46E-02 | 5.7E-04 | 8.0E-01 | 42.60 |
| 2.30E-06 | 1.45E-02 | 1.3E-04 | 8.0E-01 | 43.25 |
| 1.65E-06 | 1.45E-02 | 9.1E-05 | 8.0E-01 | 43.71 |
| 8.25E-07 | 1.45E-02 | 4.6E-05 | 8.0E-01 | 45.93 |
| 3.86E-07 | 1.45E-02 | 2.1E-05 | 8.0E-01 | 51.60 |
| 1.93E-07 | 1.45E-02 | 1.1E-05 | 8.0E-01 | 57.61 |
| 3.38E-08 | 1.46E-02 | 1.9E-06 | 8.0E-01 | 68.97 |
| 0.00E+00 | 1.46E-02 | 0.0E+00 | 8.0E-01 | 72.00 |

**Table S3.17: Brij35 + 1.6 M NaCl mixtures**

| ∝ Brij35 | ∝ NaCl | [Brij35] (M) | [NaCl] (M) | σ (mN/m) |
| --- | --- | --- | --- | --- |
| 3.33E-05 | 2.83E-02 | 1.8E-03 | 1.6E+00 | 40.43 |
| 2.34E-05 | 2.76E-02 | 1.3E-03 | 1.5E+00 | 40.16 |
| 1.81E-05 | 2.70E-02 | 9.9E-04 | 1.5E+00 | 40.66 |
| 9.43E-06 | 2.78E-02 | 5.2E-04 | 1.5E+00 | 40.18 |
| 5.35E-06 | 2.81E-02 | 2.9E-04 | 1.5E+00 | 40.90 |
| 2.69E-06 | 2.83E-02 | 1.5E-04 | 1.6E+00 | 41.15 |
| 1.76E-06 | 2.84E-02 | 9.6E-05 | 1.6E+00 | 41.83 |
| 1.42E-06 | 2.87E-02 | 7.8E-05 | 1.6E+00 | 43.25 |
| 2.41E-07 | 2.85E-02 | 1.3E-05 | 1.6E+00 | 51.85 |
| 8.13E-08 | 2.88E-02 | 4.4E-06 | 1.6E+00 | 71.08 |
| 0.00E+00 | 2.81E-02 | 0.0E+00 | 1.5E+00 | 71.64 |


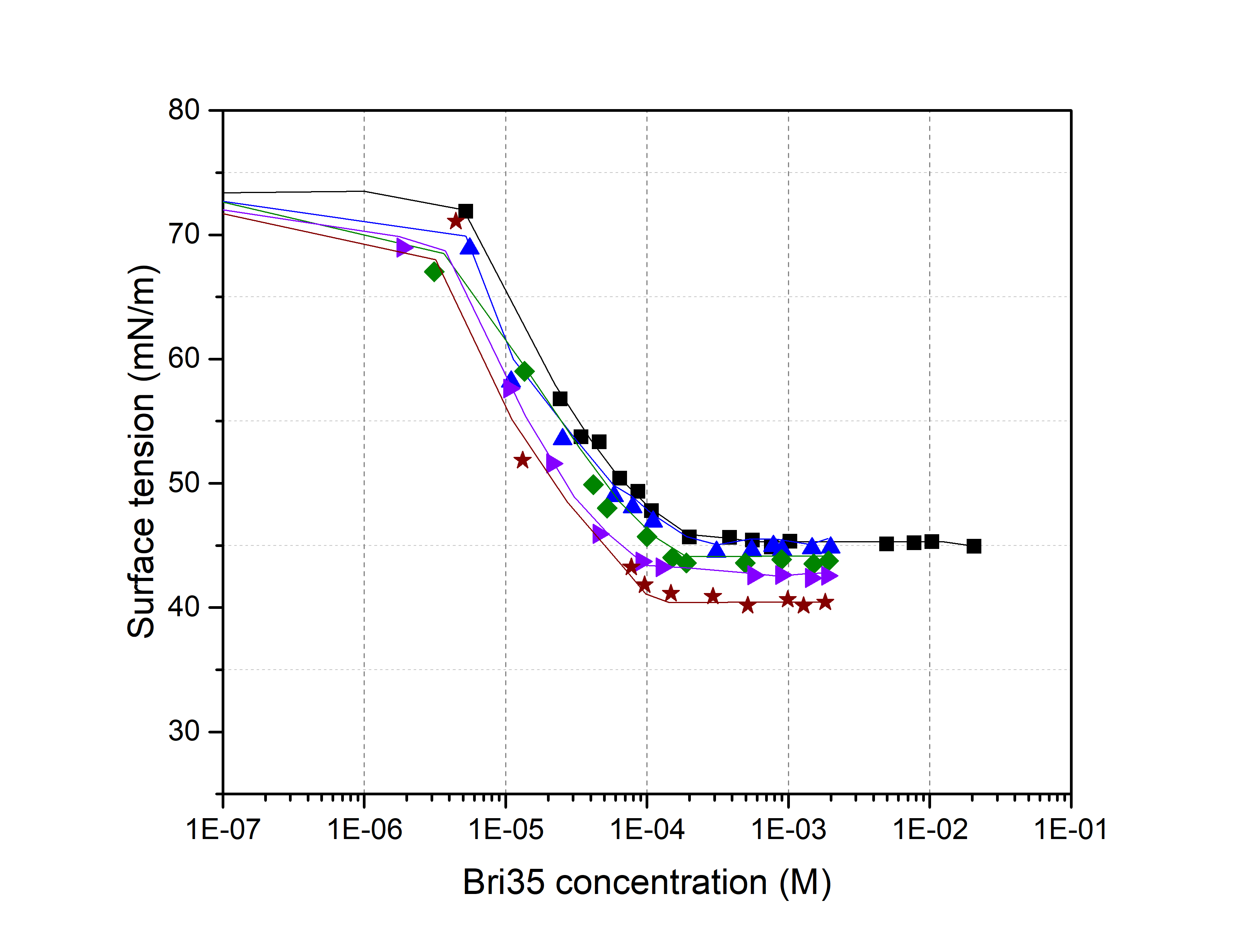


**Figure S4: Adsorption isotherms for Brij35 + NaCl mixtures:** (
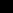
) black curve: Brij35; (
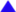
) blue curve: Brij35 + 0.021 M NaCl; (
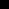
) green curve: Brij35 + 0.4 M NaCl; (
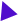
) violet curve: Brij35 + 0.8 M NaCl and (
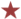
) wine curve: Brij35 + 1.6 M NaCl.

### **Evolution of the minimal surface tension with the molar fraction of the mixture**

**
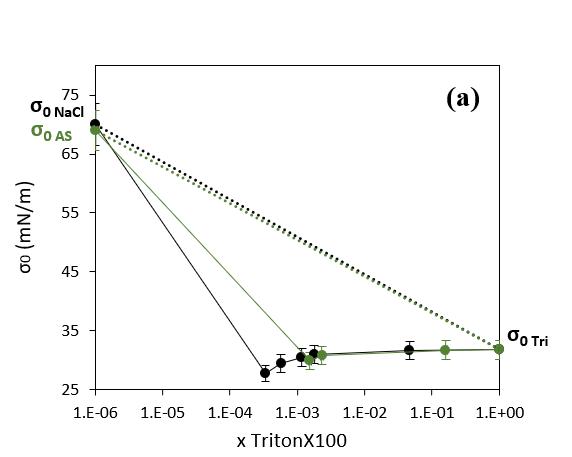
**  **
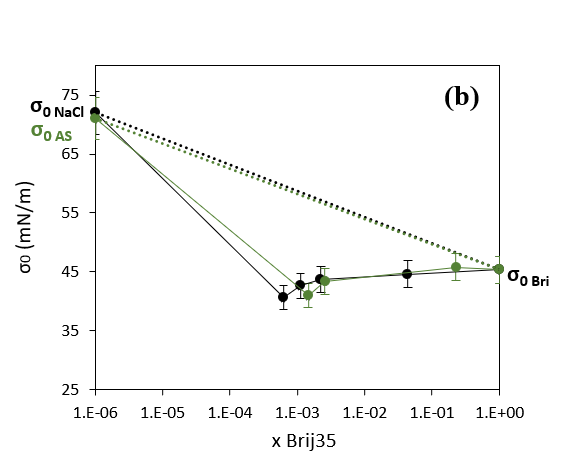
**

**Figure S5 Evolution of the minimal surface tension for surfactant + inorganic salt mixtures.** (a) (
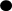
) TritonX100 + NaCl and (
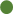
) TritonX100 + (NH_4_)_2_SO_4_ (with x_Triton_= molar fraction of TritonX100). (b) (
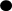
) Brij35 + NaCl and (
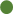
) Brij35 + (NH_4_)_2_SO_4_ (x_Brij_ = molar fraction of Brij35).

# **S4. Adsorption isotherms for organic acid and inorganic salt mixtures**

*With α = molar fraction of compounds in water.*

### **Glutaric acid + (NH_4_)_2_SO_4_**

**Table S4.1: Glutaric acid solutions**

| [Glutaric acid] (M) | σ (mN/m) | [Glutaric acid] (M) | σ (mN/m) |
| --- | --- | --- | --- |
| 3.0E+00 | 53.28 | 4.7E-02 | 72.27 |
| 2.2E+00 | 54.82 | 2.6E-02 | 72.18 |
| 1.4E+00 | 58.05 | 1.9E-02 | 72.89 |
| 7.1E-01 | 62.08 | 1.8E-02 | 72.31 |
| 5.5E-01 | 64.18 | 7.7E-03 | 73.23 |
| 2.8E-01 | 66.00 | 3.2E-03 | 73.52 |
| 7.4E-02 | 70.92 | 0.0E+00 | 73.78 |
| 5.9E-02 | 71.28 |  |  |

**Table S4.2: Glutaric acid + 0.7 M (NH_4_)_2_SO_4_** **mixtures**

| ∝ Glutaric acid | ∝ AS | [Glutaric acid] (M) | [AS] (M) | σ (mN/m) |
| --- | --- | --- | --- | --- |
| 2.98E-02 | 1.45E-02 | 1.5E+00 | 7.3E-01 | 55.95 |
| 1.37E-02 | 1.36E-02 | 7.3E-01 | 7.2E-01 | 59.55 |
| 1.09E-02 | 1.32E-02 | 5.8E-01 | 7.1E-01 | 60.94 |
| 6.89E-03 | 1.30E-02 | 3.8E-01 | 7.1E-01 | 62.57 |
| 3.86E-03 | 1.29E-02 | 2.1E-01 | 7.1E-01 | 64.87 |
| 1.33E-03 | 1.26E-02 | 7.4E-02 | 7.1E-01 | 68.69 |
| 1.14E-03 | 1.27E-02 | 6.4E-02 | 7.1E-01 | 68.76 |
| 6.74E-04 | 1.25E-02 | 3.8E-02 | 7.0E-01 | 68.96 |
| 3.44E-04 | 1.25E-02 | 1.9E-02 | 7.0E-01 | 69.68 |
| 1.16E-04 | 1.25E-02 | 6.5E-03 | 7.1E-01 | 70.16 |
| 5.39E-05 | 1.25E-02 | 3.0E-03 | 7.0E-01 | 70.86 |
| 0.00E+00 | 1.25E-02 | 0.0E+00 | 7.0E-01 | 71.34 |

**Table S4.3:** **Glutaric acid + 1.5 M (NH_4_)_2_SO_4_ mixtures**

| ∝ Glutaric acid | ∝ AS | [Glutaric acid] (M) | [AS] (M) | σ (mN/m) |
| --- | --- | --- | --- | --- |
| 3.32E-02 | 3.28E-02 | 1.7E+00 | 1.6E+00 | 53.16 |
| 1.62E-02 | 2.87E-02 | 8.7E-01 | 1.5E+00 | 55.32 |
| 1.19E-02 | 2.92E-02 | 6.5E-01 | 1.6E+00 | 57.87 |
| 7.28E-03 | 2.84E-02 | 4.0E-01 | 1.6E+00 | 60.16 |
| 4.25E-03 | 2.82E-02 | 2.4E-01 | 1.6E+00 | 61.75 |
| 1.39E-03 | 2.68E-02 | 7.9E-02 | 1.5E+00 | 66.19 |
| 1.24E-03 | 2.73E-02 | 7.0E-02 | 1.6E+00 | 66.20 |
| 4.94E-04 | 2.71E-02 | 2.8E-02 | 1.5E+00 | 68.15 |
| 3.25E-04 | 2.74E-02 | 1.9E-02 | 1.6E+00 | 68.79 |
| 1.56E-04 | 2.64E-02 | 8.9E-03 | 1.5E+00 | 69.94 |
| 8.50E-05 | 2.69E-02 | 4.9E-03 | 1.5E+00 | 69.52 |
| 0.00E+00 | 2.67E-02 | 0.0E+00 | 1.5E+00 | 69.85 |


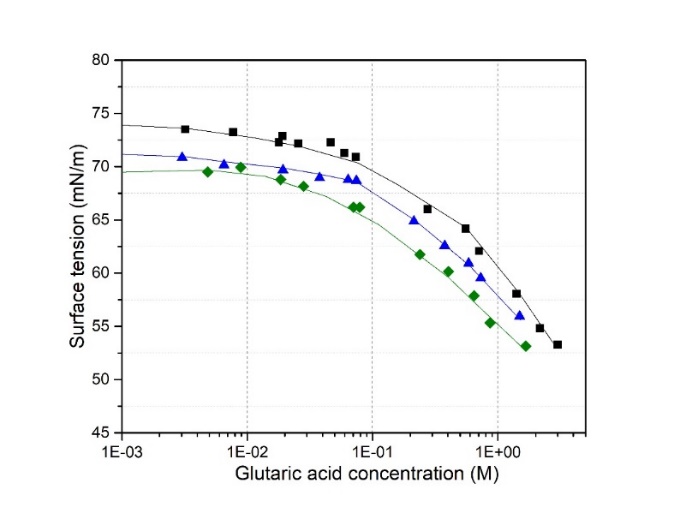


**Figure S6**: **Adsorption isotherms for Glutaric acid + (NH_4_)_2_SO_4_ mixtures**: (
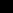
) black curve: Glutaric acid; (
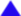
) blue curve: Glutaric acid + 0.7 M (NH_4_)_2_SO_4_; (
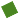
) green curve: Glutaric acid + 1.5 M (NH_4_)_2_SO_4_.

### **Glutaric acid + NaCl**

**Table S4.4: Glutaric acid + 0.4 M NaCl mixtures**

| ∝ Glutaric acid | ∝ NaCl | [Glutaric acid] (M) | [NaCl] (M) | σ (mN/m) |
| --- | --- | --- | --- | --- |
| 6.27E-02 | 9.88E-03 | 2.8E+00 | 4.4E-01 | 52.94 |
| 4.40E-02 | 8.83E-03 | 2.1E+00 | 4.2E-01 | 54.58 |
| 2.66E-02 | 8.38E-03 | 1.3E+00 | 4.2E-01 | 57.88 |
| 1.25E-02 | 7.64E-03 | 6.6E-01 | 4.1E-01 | 61.82 |
| 9.86E-03 | 7.28E-03 | 5.3E-01 | 3.9E-01 | 62.98 |
| 5.93E-03 | 7.27E-03 | 3.2E-01 | 4.0E-01 | 65.17 |
| 3.46E-03 | 7.35E-03 | 1.9E-01 | 4.1E-01 | 67.10 |
| 2.38E-03 | 6.77E-03 | 1.3E-01 | 3.7E-01 | 68.33 |
| 9.20E-04 | 6.95E-03 | 5.1E-02 | 3.9E-01 | 70.60 |
| 5.66E-04 | 6.93E-03 | 3.2E-02 | 3.9E-01 | 71.36 |
| 1.29E-04 | 6.91E-03 | 7.2E-03 | 3.9E-01 | 71.70 |
| 9.19E-05 | 6.71E-03 | 5.1E-03 | 3.8E-01 | 71.95 |
| 5.65E-05 | 6.78E-03 | 3.2E-03 | 3.8E-01 | 71.97 |
| 0.00E+00 | 6.78E-03 | 0.0E+00 | 3.8E-01 | 71.80 |

**Table S4.5: Glutaric acid + 1.7 M NaCl mixtures**

| ∝ Glutaric acid | ∝ NaCl | [Glutaric acid] (M) | [NaCl] (M) | σ (mN/m) |
| --- | --- | --- | --- | --- |
| 4.39E-02 | 3.68E-02 | 2.1E+00 | 1.8E+00 | 51.90 |
| 2.82E-02 | 3.44E-02 | 1.4E+00 | 1.8E+00 | 55.09 |
| 1.45E-02 | 3.04E-02 | 7.8E-01 | 1.6E+00 | 58.31 |
| 9.75E-03 | 3.06E-02 | 5.4E-01 | 1.7E+00 | 60.91 |
| 6.35E-03 | 3.14E-02 | 3.5E-01 | 1.8E+00 | 62.21 |
| 3.91E-03 | 2.98E-02 | 2.2E-01 | 1.7E+00 | 64.37 |
| 1.35E-03 | 2.96E-02 | 7.7E-02 | 1.7E+00 | 67.87 |
| 1.01E-03 | 2.93E-02 | 5.8E-02 | 1.7E+00 | 68.03 |
| 6.40E-04 | 2.93E-02 | 3.7E-02 | 1.7E+00 | 68.99 |
| 2.99E-04 | 3.00E-02 | 1.7E-02 | 1.7E+00 | 70.01 |
| 1.13E-04 | 2.94E-02 | 6.4E-03 | 1.7E+00 | 70.55 |
| 7.29E-05 | 2.95E-02 | 4.2E-03 | 1.7E+00 | 70.24 |
| 0.00E+00 | 2.83E-02 | 0.0E+00 | 1.6E+00 | 71.15 |

**Table S4.6: Glutaric acid + 2.5 M NaCl mixtures**

| ∝ Glutaric acid | ∝ NaCl | [Glutaric acid] (M) | [NaCl] (M) | σ (mN/m) |
| --- | --- | --- | --- | --- |
| 3.00E-02 | 5.26E-02 | 1.6E+00 | 2.7E+00 | 53.36 |
| 1.35E-02 | 4.80E-02 | 7.5E-01 | 2.6E+00 | 57.75 |
| 1.06E-02 | 4.70E-02 | 5.9E-01 | 2.6E+00 | 58.71 |
| 7.20E-03 | 4.49E-02 | 4.1E-01 | 2.5E+00 | 60.68 |
| 4.19E-03 | 4.52E-02 | 2.4E-01 | 2.6E+00 | 63.07 |
| 1.34E-03 | 4.44E-02 | 7.7E-02 | 2.6E+00 | 66.63 |
| 1.05E-03 | 4.49E-02 | 6.1E-02 | 2.6E+00 | 66.81 |
| 6.21E-04 | 4.44E-02 | 3.6E-02 | 2.6E+00 | 68.55 |
| 1.50E-04 | 4.41E-02 | 8.7E-03 | 2.6E+00 | 69.41 |
| 1.03E-04 | 4.22E-02 | 5.9E-03 | 2.4E+00 | 69.80 |
| 6.72E-05 | 4.45E-02 | 3.9E-03 | 2.6E+00 | 69.58 |
| 0.00E+00 | 4.26E-02 | 0.0E+00 | 2.5E+00 | 70.50 |


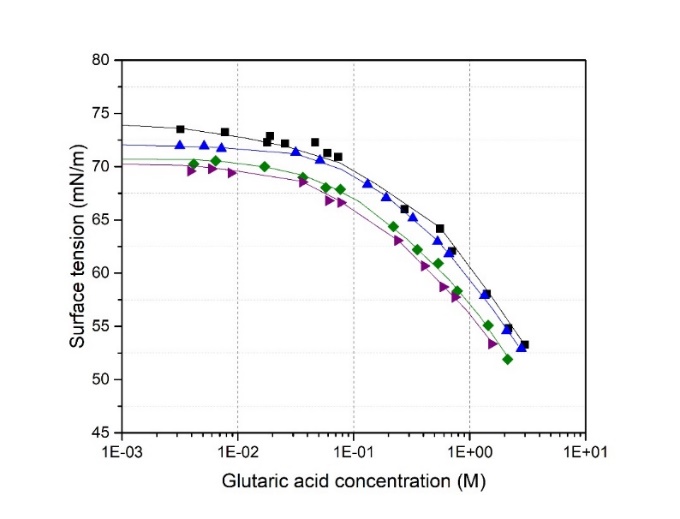


**Figure S7:** **Adsorption isotherms for Glutaric acid + NaCl mixtures**: (
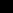
) black curve: Glutaric acid; (
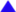
) blue curve: Glutaric acid + 0.4 M NaCl; (
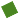
) green curve: Glutaric acid + 1.7 M NaCl; (
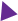
) purple curve: Glutaric acid + 2.5 M NaCl

### **Effects of inorganic salts on the surface tension of glutaric acid**


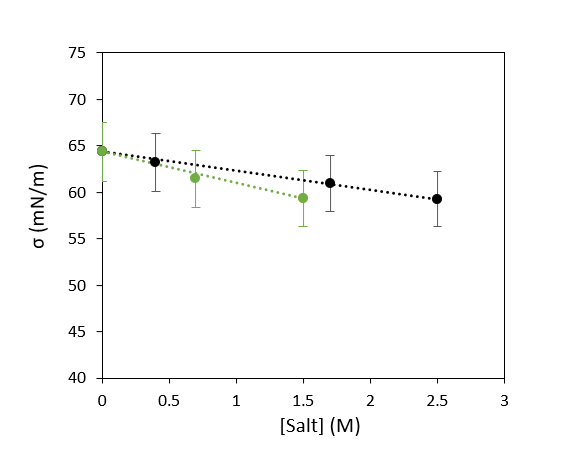


**Figure S8**: **Effects of inorganic salts on the surface tension of glutaric acid.** Variation of the surface tension for glutaric acid + salt mixtures, at [Glutaric acid] = 0.5 M: (
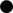
) with NaCl; (
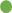
) with (NH_4_)_2_SO_4_.

# **S5.** **Adsorption isotherms for amphiphilic surfactant and organic acid mixtures**

*With α = molar fraction of compounds in water and x the organic molar fraction of surfactant.*

### **TritonX100 + Glutaric acid**

**Table S5.1: TritonX100 + Glutaric acid mixtures (x = 0.13)**

| $\boldsymbol{\propto}$Glutaric acid | ∝ TritonX100 | [Glutaric acid] (M) | [TritonX100] (M) | σ (mN/m) |
| --- | --- | --- | --- | --- |
| 6.41E-03 | 9.53E-04 | 3.4E-01 | 5.0E-02 | 33.24 |
| 1.21E-03 | 1.80E-04 | 6.8E-02 | 1.0E-02 | 32.85 |
| 7.24E-04 | 1.08E-04 | 4.0E-02 | 6.0E-03 | 32.67 |
| 3.59E-04 | 5.33E-05 | 2.0E-02 | 3.0E-03 | 32.56 |
| 1.26E-04 | 1.87E-05 | 7.0E-03 | 1.0E-03 | 31.83 |
| 1.01E-04 | 1.50E-05 | 5.6E-03 | 8.3E-04 | 32.20 |
| 6.58E-05 | 9.79E-06 | 3.7E-03 | 5.4E-04 | 31.94 |
| 4.41E-05 | 6.56E-06 | 2.4E-03 | 3.6E-04 | 31.98 |
| 1.92E-05 | 2.86E-06 | 1.1E-03 | 1.6E-04 | 38.20 |
| 1.20E-05 | 1.78E-06 | 6.6E-04 | 9.9E-05 | 43.25 |
| 7.16E-06 | 1.07E-06 | 4.0E-04 | 5.9E-05 | 48.02 |
| 3.54E-06 | 5.26E-07 | 2.0E-04 | 2.9E-05 | 54.43 |
| 1.15E-06 | 1.71E-07 | 6.4E-05 | 9.5E-06 | 67.27 |
| 6.93E-07 | 1.03E-07 | 3.8E-05 | 5.7E-06 | 71.61 |
| 3.46E-07 | 5.14E-08 | 1.9E-05 | 2.9E-06 | 72.69 |
| 0.00E+00 | 0.00E+00 | 0.0E+00 | 0.0E+00 | 73.40 |

**Table S5.2: TritonX100 + Glutaric acid** **mixtures (x = 0.016)**

| ∝ Glutaric acid | ∝ TritonX100 | [Glutaric acid] (M) | [TritonX100] (M) | σ (mN/m) |
| --- | --- | --- | --- | --- |
| 2.72E-02 | 4.50E-04 | 1.3E+00 | 2.2E-02 | 33.89 |
| 1.27E-02 | 2.07E-04 | 7.1E-01 | 1.2E-02 | 33.78 |
| 7.32E-03 | 1.20E-04 | 4.1E-01 | 6.7E-03 | 33.10 |
| 3.60E-03 | 5.92E-05 | 2.0E-01 | 3.3E-03 | 33.11 |
| 1.28E-03 | 2.12E-05 | 7.1E-02 | 1.2E-03 | 32.56 |
| 1.01E-03 | 1.66E-05 | 5.6E-02 | 9.2E-04 | 32.55 |
| 7.50E-04 | 1.24E-05 | 4.2E-02 | 6.9E-04 | 32.35 |
| 5.16E-04 | 8.52E-06 | 2.9E-02 | 4.7E-04 | 32.16 |
| 2.53E-04 | 4.18E-06 | 1.4E-02 | 2.3E-04 | 35.80 |
| 1.26E-04 | 2.08E-06 | 7.0E-03 | 1.2E-04 | 41.83 |
| 7.53E-05 | 1.24E-06 | 4.2E-03 | 6.9E-05 | 48.99 |
| 3.75E-05 | 6.20E-07 | 2.1E-03 | 3.4E-05 | 55.61 |
| 1.23E-05 | 2.03E-07 | 6.8E-04 | 1.1E-05 | 68.40 |
| 7.36E-06 | 1.22E-07 | 4.1E-04 | 6.8E-06 | 72.41 |
| 3.67E-06 | 6.07E-08 | 2.0E-04 | 3.4E-06 | 72.93 |
| 0.00E+00 | 0.00E+00 | 0.0E+00 | 0.0E+00 | 73.90 |

**Table S5.3: TritonX100 + Glutaric acid mixtures (x = 0.00167)**

| ∝ Glutaric acid | ∝ TritonX100 | [Glutaric acid] (M) | [TritonX100] (M) | σ (mN/m) |
| --- | --- | --- | --- | --- |
| 5.44E-02 | 8.99E-05 | 2.5E+00 | 4.1E-03 | 36.30 |
| 4.19E-02 | 6.65E-05 | 2.3E+00 | 3.8E-03 | 35.25 |
| 2.31E-02 | 3.73E-05 | 1.3E+00 | 2.1E-03 | 33.68 |
| 1.34E-02 | 2.19E-05 | 7.5E-01 | 1.2E-03 | 33.99 |
| 9.94E-03 | 1.63E-05 | 5.5E-01 | 9.1E-04 | 34.11 |
| 4.71E-03 | 7.74E-06 | 2.6E-01 | 4.3E-04 | 35.72 |
| 2.93E-03 | 4.83E-06 | 1.6E-01 | 2.7E-04 | 37.80 |
| 1.45E-03 | 2.40E-06 | 8.1E-02 | 1.3E-04 | 41.53 |
| 7.16E-04 | 1.18E-06 | 4.0E-02 | 6.6E-05 | 48.62 |
| 3.52E-04 | 5.82E-07 | 2.0E-02 | 3.2E-05 | 55.99 |
| 1.75E-04 | 2.88E-07 | 9.7E-03 | 1.6E-05 | 67.50 |
| 8.68E-05 | 1.43E-07 | 4.8E-03 | 8.0E-06 | 71.97 |
| 4.32E-05 | 7.13E-08 | 2.4E-03 | 4.0E-06 | 73.82 |
| 0.00E+00 | 0.00E+00 | 0.0E+00 | 0.0E+00 | 73.74 |

**Table S5.4: TritonX100 + Glutaric acid** **mixtures (x = 0.00015)**

| ∝ Glutaric acid | ∝ TritonX100 | [Glutaric acid] (M) | [TritonX100] (M) | σ (mN/m) |
| --- | --- | --- | --- | --- |
| 5.47E-02 | 8.26E-06 | 2.5E+00 | 3.7E-04 | 46.42 |
| 4.26E-02 | 6.17E-06 | 2.4E+00 | 3.6E-04 | 46.61 |
| 3.09E-02 | 4.52E-06 | 1.7E+00 | 2.6E-04 | 46.92 |
| 1.40E-02 | 2.08E-06 | 7.8E-01 | 1.2E-04 | 46.50 |
| 6.57E-03 | 9.85E-07 | 3.6E-01 | 5.5E-05 | 48.65 |
| 3.20E-03 | 4.81E-07 | 1.8E-01 | 2.7E-05 | 55.16 |
| 1.57E-03 | 2.37E-07 | 8.7E-02 | 1.3E-05 | 63.30 |
| 7.67E-04 | 1.16E-07 | 4.3E-02 | 6.4E-06 | 70.83 |
| 3.90E-04 | 5.88E-08 | 2.2E-02 | 3.3E-06 | 72.13 |
| 1.99E-04 | 3.00E-08 | 1.1E-02 | 1.7E-06 | 72.65 |
| 1.01E-04 | 1.53E-08 | 5.6E-03 | 8.5E-07 | 73.30 |
| 5.00E-05 | 7.55E-09 | 2.8E-03 | 4.2E-07 | 73.42 |
| 2.46E-05 | 3.71E-09 | 1.4E-03 | 2.1E-07 | 73.77 |
| 0.00E+00 | 0.00E+00 | 0.0E+00 | 0.0E+00 | 73.83 |


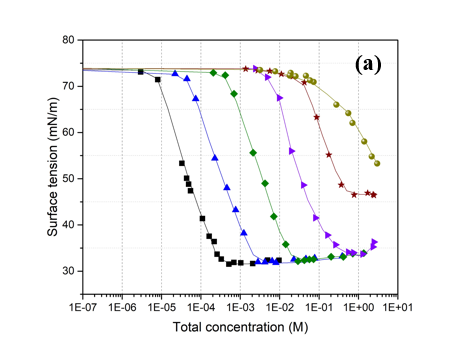

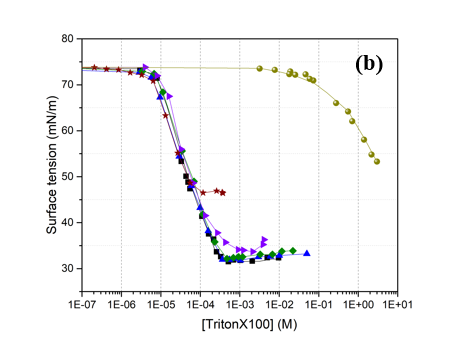


**Figure S9**: **Adsorption isotherm for TritonX100 + Glutaric acid mixtures****:** **(a) function of the total concentration ([TritonX100] + [Glutaric acid]) and (b) function of the concentration of TritonX100.**  (
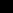
) black curve TritonX100; (
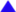
) blue curve: x = 0.13; (
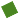
) green curve: x = 0.016; (
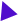
) violet curve: x = 0.00167; (
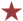
) wine curve: x = 0.00015 and (
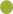
) yellow curve: Glutaric acid.

**Table S5.5: TritonX100 + 0.065 M Glutaric acid mixtures**

| ∝ Glutaric acid | ∝ TritonX100 | [Glutaric acid] (M) | [TritonX100] (M) | σ (mN/m) |
| --- | --- | --- | --- | --- |
| 1.43E-03 | 1.81E-04 | 7.9E-02 | 1.0E-02 | 32.67 |
| 1.20E-03 | 8.74E-05 | 6.7E-02 | 4.8E-03 | 32.60 |
| 1.21E-03 | 1.61E-05 | 6.7E-02 | 8.9E-04 | 32.63 |
| 1.18E-03 | 1.31E-05 | 6.5E-02 | 7.2E-04 | 32.50 |
| 1.19E-03 | 8.64E-06 | 6.6E-02 | 4.8E-04 | 33.75 |
| 1.16E-03 | 6.50E-06 | 6.4E-02 | 3.6E-04 | 35.02 |
| 1.15E-03 | 4.93E-06 | 6.4E-02 | 2.7E-04 | 35.40 |
| 1.16E-03 | 3.64E-06 | 6.4E-02 | 2.0E-04 | 36.92 |
| 1.21E-03 | 1.66E-06 | 6.7E-02 | 9.2E-05 | 43.88 |
| 1.11E-03 | 1.24E-06 | 6.2E-02 | 6.8E-05 | 45.63 |
| 1.16E-03 | 7.76E-07 | 6.4E-02 | 4.3E-05 | 49.65 |
| 1.14E-03 | 4.78E-07 | 6.3E-02 | 2.6E-05 | 54.75 |
| 1.16E-03 | 4.02E-07 | 6.4E-02 | 2.2E-05 | 56.04 |
| 1.15E-03 | 1.13E-07 | 6.4E-02 | 6.2E-06 | 67.50 |
| 1.22E-03 | 0.00E+00 | 6.7E-02 | 0.0E+00 | 70.57 |

**Table S5.6: TritonX100 + 0.65 M Glutaric acid mixtures**

| ∝ Glutaric acid | ∝ TritonX100 | [Glutaric acid] (M) | [TritonX100] (M) | σ (mN/m) |
| --- | --- | --- | --- | --- |
| 1.22E-02 | 1.93E-04 | 6.4E-01 | 1.0E-02 | 33.88 |
| 1.31E-02 | 9.70E-05 | 6.9E-01 | 5.1E-03 | 33.76 |
| 1.22E-02 | 1.73E-05 | 6.5E-01 | 9.1E-04 | 33.43 |
| 1.23E-02 | 1.37E-05 | 6.5E-01 | 7.3E-04 | 33.92 |
| 1.23E-02 | 8.80E-06 | 6.5E-01 | 4.7E-04 | 37.40 |
| 1.20E-02 | 7.23E-06 | 6.4E-01 | 3.8E-04 | 38.47 |
| 1.21E-02 | 5.23E-06 | 6.4E-01 | 2.8E-04 | 39.67 |
| 1.21E-02 | 3.98E-06 | 6.4E-01 | 2.1E-04 | 41.85 |
| 1.21E-02 | 2.45E-06 | 6.4E-01 | 1.3E-04 | 44.76 |
| 1.22E-02 | 1.34E-06 | 6.4E-01 | 7.1E-05 | 48.85 |
| 1.22E-02 | 1.13E-06 | 6.5E-01 | 6.0E-05 | 49.28 |
| 1.21E-02 | 8.40E-07 | 6.4E-01 | 4.4E-05 | 49.57 |
| 1.20E-02 | 4.25E-07 | 6.4E-01 | 2.2E-05 | 53.74 |
| 1.22E-02 | 3.91E-07 | 6.5E-01 | 2.1E-05 | 54.43 |
| 1.19E-02 | 1.25E-07 | 6.3E-01 | 6.6E-06 | 60.66 |
| 1.21E-02 | 0.00E+00 | 6.4E-01 | 0.0E+00 | 62.15 |

**Table S5.7: TritonX100 + 2.7 M Glutaric acid** **mixtures**

| ∝ Glutaric acid | ∝ TritonX100 | [Glutaric acid] (M) | [TritonX100] (M) | σ (mN/m) |
| --- | --- | --- | --- | --- |
| 5.56E-02 | 4.39E-04 | 2.5E+00 | 2.0E-02 | 34.34 |
| 6.26E-02 | 3.07E-04 | 2.8E+00 | 1.4E-02 | 33.85 |
| 5.67E-02 | 9.72E-05 | 2.6E+00 | 4.4E-03 | 36.62 |
| 6.43E-02 | 2.35E-05 | 2.8E+00 | 1.0E-03 | 44.30 |
| 6.20E-02 | 1.70E-05 | 2.7E+00 | 7.5E-04 | 44.38 |
| 6.36E-02 | 1.20E-05 | 2.8E+00 | 5.3E-04 | 46.50 |
| 6.10E-02 | 8.69E-06 | 2.7E+00 | 3.8E-04 | 47.60 |
| 6.11E-02 | 6.18E-06 | 2.7E+00 | 2.7E-04 | 48.80 |
| 6.28E-02 | 4.51E-06 | 2.8E+00 | 2.0E-04 | 49.90 |
| 6.24E-02 | 2.42E-06 | 2.7E+00 | 1.1E-04 | 50.60 |
| 6.38E-02 | 1.91E-06 | 2.8E+00 | 8.4E-05 | 50.60 |
| 6.30E-02 | 1.08E-06 | 2.8E+00 | 4.7E-05 | 51.70 |
| 6.30E-02 | 7.85E-07 | 2.8E+00 | 3.4E-05 | 52.20 |
| 6.34E-02 | 3.47E-07 | 2.8E+00 | 1.5E-05 | 52.40 |
| 6.48E-02 | 3.22E-07 | 2.8E+00 | 1.4E-05 | 52.10 |
| 6.11E-02 | 1.86E-07 | 2.7E+00 | 8.2E-06 | 53.51 |
| 6.24E-02 | 0.00E+00 | 2.7E+00 | 0.0E+00 | 53.73 |


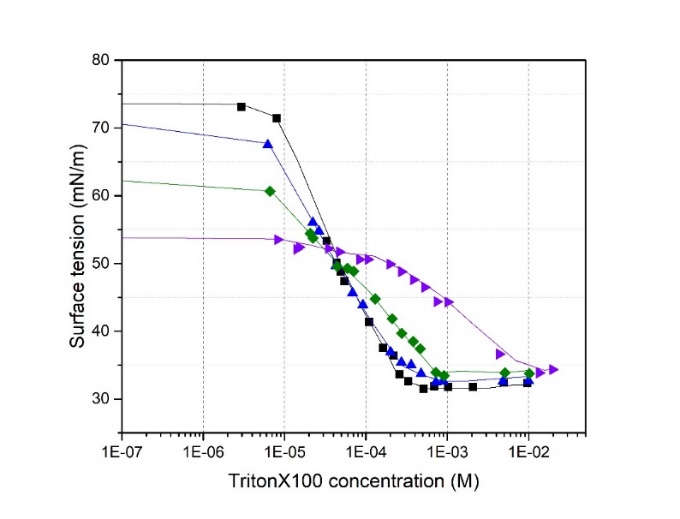


**Figure S10**: **Adsorption isotherm for TritonX100 + Glutaric acid mixtures**: (
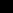
) black curve: TritonX100; (
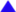
) blue curve: TritonX100 + 0.065 M glutaric acid; (
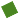
) green curve: TritonX100 + 0.65 M glutaric acid and (
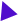
) violet curve: TritonX100 + 2.7 M glutaric acid.

### **Brij35 + Glutaric acid**

**Table S5.8: Brij35 + Glutaric acid** **mixtures (x = 0.13)**

| ∝ Glutaric acid | ∝ Brij35 | [Glutaric acid] (M) | [Brij35] (M) | σ (mN/m) |
| --- | --- | --- | --- | --- |
| 1.15E-03 | 1.75E-04 | 6.3E-02 | 9.6E-03 | 45.08 |
| 9.17E-04 | 1.39E-04 | 5.1E-02 | 7.7E-03 | 45.05 |
| 5.69E-04 | 8.62E-05 | 3.2E-02 | 4.8E-03 | 44.95 |
| 2.83E-04 | 4.29E-05 | 1.6E-02 | 2.4E-03 | 45.31 |
| 1.12E-04 | 1.69E-05 | 6.2E-03 | 9.4E-04 | 45.35 |
| 5.54E-05 | 8.40E-06 | 3.1E-03 | 4.7E-04 | 45.11 |
| 2.73E-05 | 4.14E-06 | 1.5E-03 | 2.3E-04 | 46.02 |
| 1.09E-05 | 1.65E-06 | 6.1E-04 | 9.2E-05 | 48.92 |
| 8.71E-06 | 1.32E-06 | 4.8E-04 | 7.3E-05 | 50.96 |
| 5.45E-06 | 8.27E-07 | 3.0E-04 | 4.6E-05 | 53.69 |
| 2.72E-06 | 4.13E-07 | 1.5E-04 | 2.3E-05 | 57.69 |
| 1.09E-06 | 1.65E-07 | 6.0E-05 | 9.2E-06 | 61.91 |
| 6.35E-07 | 9.64E-08 | 3.5E-05 | 5.4E-06 | 70.74 |
| 0.00E+00 | 0.00E+00 | 0.0E+00 | 0.0E+00 | 73.40 |

**Table S5.9: Brij35 + Glutaric acid mixtures (****x = 0.014)**

| ∝ Glutaric acid | ∝ Brij35 | [Glutaric acid] (M) | [Brij35] (M) | σ (mN/m) |
| --- | --- | --- | --- | --- |
| 1.34E-02 | 1.86E-04 | 6.9E-01 | 9.7E-03 | 43.24 |
| 1.05E-02 | 1.46E-04 | 5.8E-01 | 8.2E-03 | 43.67 |
| 6.37E-03 | 8.91E-05 | 3.5E-01 | 5.0E-03 | 43.81 |
| 3.09E-03 | 4.33E-05 | 1.7E-01 | 2.4E-03 | 44.44 |
| 1.21E-03 | 1.70E-05 | 6.7E-02 | 9.5E-04 | 44.74 |
| 5.98E-04 | 8.41E-06 | 3.3E-02 | 4.7E-04 | 45.30 |
| 2.99E-04 | 4.21E-06 | 1.7E-02 | 2.3E-04 | 45.55 |
| 1.18E-04 | 1.66E-06 | 6.5E-03 | 9.2E-05 | 48.57 |
| 9.41E-05 | 1.32E-06 | 5.2E-03 | 7.4E-05 | 49.24 |
| 5.85E-05 | 8.23E-07 | 3.3E-03 | 4.6E-05 | 53.09 |
| 2.91E-05 | 4.09E-07 | 1.6E-03 | 2.3E-05 | 57.01 |
| 1.20E-05 | 1.69E-07 | 6.7E-04 | 9.4E-06 | 61.55 |
| 7.21E-06 | 1.01E-07 | 4.0E-04 | 5.6E-06 | 68.61 |
| 0.00E+00 | 0.00E+00 | 0.0E+00 | 0.0E+00 | 73.90 |

**Table S5.10: Brij35 + Glutaric acid mixtures (x = 0.0015)**

| ∝ Glutaric acid | ∝ Brij35 | [Glutaric acid] (M) | [Brij35] (M) | σ (mN/m) |
| --- | --- | --- | --- | --- |
| 4.52E-02 | 6.77E-05 | 2.1E+00 | 3.2E-03 | 40.45 |
| 3.51E-02 | 5.09E-05 | 2.0E+00 | 2.9E-03 | 41.90 |
| 1.99E-02 | 2.92E-05 | 1.1E+00 | 1.7E-03 | 42.77 |
| 9.41E-03 | 1.40E-05 | 5.2E-01 | 7.8E-04 | 43.47 |
| 3.31E-03 | 4.94E-06 | 1.8E-01 | 2.8E-04 | 43.86 |
| 1.62E-03 | 2.42E-06 | 9.0E-02 | 1.4E-04 | 47.31 |
| 7.96E-04 | 1.19E-06 | 4.4E-02 | 6.6E-05 | 52.60 |
| 2.91E-04 | 4.36E-07 | 1.6E-02 | 2.4E-05 | 56.87 |
| 1.46E-04 | 2.19E-07 | 8.1E-03 | 1.2E-05 | 62.70 |
| 9.07E-05 | 1.36E-07 | 5.0E-03 | 7.6E-06 | 66.77 |
| 4.52E-05 | 6.78E-08 | 2.5E-03 | 3.8E-06 | 73.73 |
| 2.23E-05 | 3.34E-08 | 1.2E-03 | 1.9E-06 | 73.90 |
| 1.09E-05 | 1.64E-08 | 6.1E-04 | 9.1E-07 | 74.05 |
| 0.00E+00 | 0.00E+00 | 0.0E+00 | 0.0E+00 | 73.74 |

**Table S5.11: Adsorption isotherm for Brij35 + Glutaric acid (x = 0.00014)**

| ∝ Glutaric acid | ∝ Brij35 | [Glutaric acid] (M) | [Brij35] (M) | σ (mN/m) |
| --- | --- | --- | --- | --- |
| 5.39E-02 | 7.54E-06 | 2.4E+00 | 3.4E-04 | 44.03 |
| 4.12E-02 | 5.55E-06 | 2.3E+00 | 3.2E-04 | 45.47 |
| 2.27E-02 | 3.10E-06 | 1.3E+00 | 1.8E-04 | 45.70 |
| 1.07E-02 | 1.48E-06 | 5.9E-01 | 8.3E-05 | 47.46 |
| 3.90E-03 | 5.45E-07 | 2.2E-01 | 3.0E-05 | 53.17 |
| 1.88E-03 | 2.63E-07 | 1.0E-01 | 1.5E-05 | 58.08 |
| 9.41E-04 | 1.32E-07 | 5.2E-02 | 7.3E-06 | 63.78 |
| 3.43E-04 | 4.81E-08 | 1.9E-02 | 2.7E-06 | 72.13 |
| 1.69E-04 | 2.36E-08 | 9.4E-03 | 1.3E-06 | 72.73 |
| 8.44E-05 | 1.18E-08 | 4.7E-03 | 6.6E-07 | 73.13 |
| 4.22E-05 | 5.91E-09 | 2.3E-03 | 3.3E-07 | 73.10 |
| 2.11E-05 | 2.95E-09 | 1.2E-03 | 1.6E-07 | 73.41 |
| 1.05E-05 | 1.48E-09 | 5.9E-04 | 8.2E-08 | 73.44 |
| 0.00E+00 | 0.00E+00 | 0.0E+00 | 0.0E+00 | 73.83 |


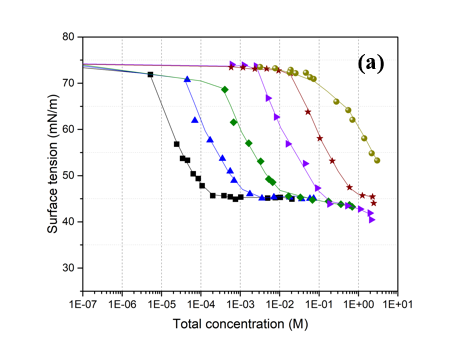

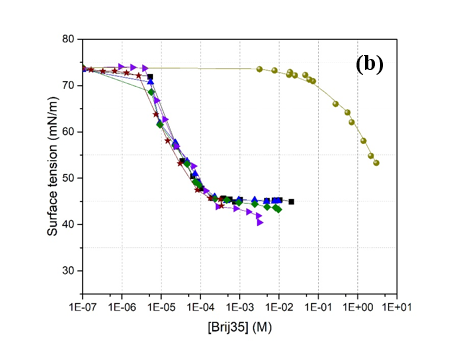


**Figure S11**: **Adsorption isotherms for Brij35 + Glutaric acid mixtures**: **(a) function of the total concentration ([Brij35] + [Glutaric acid]) and (b) function of the concentration of Brij35.** (
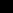
) black curve: Brij35; (
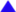
) blue curve: x = 0.13; (
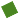
) green curve: x = 0.014; (
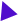
) violet curve: x = 0.0015; (
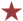
) wine curve: x = 0.00014 and (
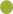
) yellow curve: Glutaric acid.

**Table S5.12: Adsorption isotherm for Brij35 + 0.65 M Glutaric acid mixtures**

| ∝ Glutaric acid | ∝ Brij35 | [Glutaric acid] (M) | [Brij35] (M) | σ (mN/m) |
| --- | --- | --- | --- | --- |
| 1.27E-02 | 1.87E-04 | 6.7E-01 | 9.9E-03 | 43.39 |
| 1.24E-02 | 1.49E-04 | 6.5E-01 | 7.9E-03 | 43.53 |
| 1.26E-02 | 9.56E-05 | 6.7E-01 | 5.1E-03 | 43.12 |
| 1.26E-02 | 1.90E-05 | 6.7E-01 | 1.0E-03 | 43.51 |
| 1.23E-02 | 1.50E-05 | 6.5E-01 | 7.9E-04 | 43.89 |
| 1.27E-02 | 9.61E-06 | 6.7E-01 | 5.1E-04 | 43.25 |
| 1.27E-02 | 6.59E-06 | 6.7E-01 | 3.5E-04 | 43.70 |
| 1.26E-02 | 2.07E-06 | 6.7E-01 | 1.1E-04 | 46.58 |
| 1.24E-02 | 1.53E-06 | 6.6E-01 | 8.1E-05 | 47.67 |
| 1.28E-02 | 1.17E-06 | 6.7E-01 | 6.2E-05 | 49.09 |
| 1.30E-02 | 7.52E-07 | 6.8E-01 | 4.0E-05 | 50.88 |
| 1.26E-02 | 4.18E-07 | 6.7E-01 | 2.2E-05 | 53.37 |
| 1.26E-02 | 3.75E-07 | 6.7E-01 | 2.0E-05 | 53.47 |
| 1.21E-02 | 2.34E-07 | 6.4E-01 | 1.2E-05 | 54.64 |
| 1.15E-02 | 1.15E-07 | 6.1E-01 | 6.1E-06 | 59.44 |
| 1.24E-02 | 0.00E+00 | 6.5E-01 | 0.0E+00 | 62.80 |

**Table S5.13: Adsorption isotherm for Brij35 + 2 M Glutaric acid mixtures**

| ∝ Glutaric acid | ∝ Brij35 | [Glutaric acid] (M) | [Brij35] (M) | σ (mN/m) |
| --- | --- | --- | --- | --- |
| 4.37E-02 | 1.17E-04 | 2.1E+00 | 5.5E-03 | 40.77 |
| 4.43E-02 | 2.33E-05 | 2.1E+00 | 1.1E-03 | 41.20 |
| 4.44E-02 | 1.86E-05 | 2.1E+00 | 8.8E-04 | 41.86 |
| 4.43E-02 | 1.17E-05 | 2.1E+00 | 5.5E-04 | 42.80 |
| 4.37E-02 | 8.24E-06 | 2.1E+00 | 3.9E-04 | 44.09 |
| 4.41E-02 | 3.37E-06 | 2.1E+00 | 1.6E-04 | 44.73 |
| 4.44E-02 | 1.86E-06 | 2.1E+00 | 8.8E-05 | 47.38 |
| 4.43E-02 | 1.31E-06 | 2.1E+00 | 6.1E-05 | 46.97 |
| 4.43E-02 | 9.58E-07 | 2.1E+00 | 4.5E-05 | 48.18 |
| 4.41E-02 | 4.95E-07 | 2.1E+00 | 2.3E-05 | 50.09 |
| 4.42E-02 | 2.94E-07 | 2.1E+00 | 1.4E-05 | 49.45 |
| 4.16E-02 | 2.56E-07 | 2.0E+00 | 1.2E-05 | 51.15 |
| 4.60E-02 | 1.16E-07 | 2.1E+00 | 5.4E-06 | 52.63 |
| 4.43E-02 | 0.00E+00 | 2.1E+00 | 0.0E+00 | 54.82 |


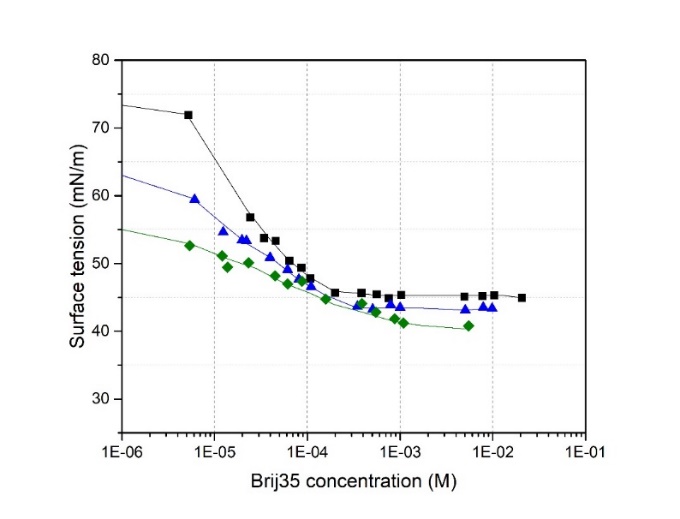


**Figure S12**: **Adsorption isotherms for Brij35 + Glutaric acid mixtures**: (
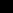
) black curve: Brij35; (
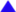
) blue curve: Brij35 + 0.65 M glutaric acid; (
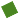
) green curve: Brij35 + 2 M glutaric acid.

### **TritonX114 + Glutaric acid**

**Table S5.14: TritonX114 solutions**

| [TritonX114] (M) | σ (mN/m) | [TritonX114] (M) | σ (mN/m) |
| --- | --- | --- | --- |
| 9.9E-03 | 30.14 | 9.5E-05 | 39.88 |
| 7.0E-03 | 30.16 | 4.7E-05 | 46.17 |
| 3.7E-03 | 29.67 | 2.2E-05 | 58.00 |
| 1.9E-03 | 29.83 | 9.1E-06 | 69.32 |
| 9.1E-04 | 29.63 | 4.0E-06 | 72.71 |
| 4.5E-04 | 29.48 | 0.0E+00 | 73.51 |
| 2.2E-04 | 31.54 |  |  |

**Table S5.15: TritonX114 + 0.6 M Glutaric acid mixtures**

| ∝ Glutaric acid | ∝ TritonX114 | [Glutaric acid] (M) | [TritonX114] (M) | σ (mN/m) |
| --- | --- | --- | --- | --- |
| 1.25E-02 | 8.79E-05 | 6.6E-01 | 4.6E-03 | 30.80 |
| 1.27E-02 | 2.60E-05 | 6.7E-01 | 1.4E-03 | 30.56 |
| 1.29E-02 | 1.40E-05 | 6.8E-01 | 7.4E-04 | 31.16 |
| 1.27E-02 | 1.01E-05 | 6.7E-01 | 5.3E-04 | 33.02 |
| 1.27E-02 | 6.62E-06 | 6.7E-01 | 3.5E-04 | 35.11 |
| 1.24E-02 | 4.06E-06 | 6.6E-01 | 2.1E-04 | 38.36 |
| 1.26E-02 | 1.79E-06 | 6.6E-01 | 9.4E-05 | 45.68 |
| 1.23E-02 | 8.65E-07 | 6.5E-01 | 4.6E-05 | 51.01 |
| 1.24E-02 | 4.37E-07 | 6.6E-01 | 2.3E-05 | 56.53 |
| 1.25E-02 | 2.09E-07 | 6.6E-01 | 1.1E-05 | 58.64 |
| 1.19E-02 | 1.65E-07 | 6.3E-01 | 8.7E-06 | 59.15 |
| 1.14E-02 | 1.32E-07 | 6.1E-01 | 7.0E-06 | 59.55 |
| 1.29E-02 | 0.0E+00 | 6.8E-01 | 0.0E+00 | 62.08 |

**Table S5.16: TritonX114 + 2 M Glutaric acid mixtures**

| ∝ Glutaric acid | ∝ TritonX114 | [Glutaric acid] (M) | [TritonX114] (M) | σ (mN/m) |
| --- | --- | --- | --- | --- |
| 4.05E-02 | 9.17E-04 | 1.9E+00 | 4.4E-02 | 31.76 |
| 4.02E-02 | 2.80E-04 | 1.9E+00 | 1.3E-02 | 31.83 |
| 4.68E-02 | 1.40E-04 | 2.2E+00 | 6.5E-03 | 31.30 |
| 4.53E-02 | 6.23E-05 | 2.1E+00 | 2.9E-03 | 33.00 |
| 4.42E-02 | 3.07E-05 | 2.1E+00 | 1.4E-03 | 37.01 |
| 4.52E-02 | 1.69E-05 | 2.1E+00 | 7.9E-04 | 40.21 |
| 4.52E-02 | 1.13E-05 | 2.1E+00 | 5.3E-04 | 42.83 |
| 4.44E-02 | 8.27E-06 | 2.1E+00 | 3.9E-04 | 44.24 |
| 4.42E-02 | 5.05E-06 | 2.1E+00 | 2.4E-04 | 44.86 |
| 4.48E-02 | 2.35E-06 | 2.1E+00 | 1.1E-04 | 48.45 |
| 4.45E-02 | 1.23E-06 | 2.1E+00 | 5.8E-05 | 48.83 |
| 4.61E-02 | 6.48E-07 | 2.1E+00 | 3.0E-05 | 50.13 |
| 4.45E-02 | 2.49E-07 | 2.1E+00 | 1.2E-05 | 51.05 |
| 4.38E-02 | 2.23E-07 | 2.1E+00 | 1.0E-05 | 50.42 |
| 4.13E-02 | 9.01E-08 | 2.0E+00 | 4.3E-06 | 53.63 |
| 4.68E-02 | 0.0E+00 | 2.2E+00 | 0.0E+00 | 54.82 |


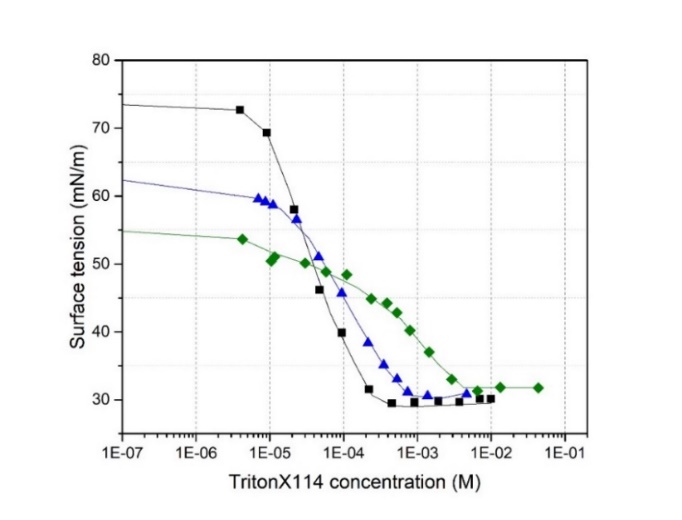


**Figure S13**: **Adsorption isotherms for TritonX114 + Glutaric acid mixtures**: (
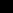
) black curve: TritonX114; (
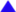
) blue curve: TritonX114 + 0.6 M glutaric acid; (
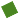
) green curve: TritonX114 + 2 M glutaric acid.

### **TritonX100 + Oxalic acid**

**Table S5.17: Oxalic acid solutions**

| [Oxalic acid] (M) | σ (mN/m) | [Oxalic acid] (M) | σ (mN/m) |
| --- | --- | --- | --- |
| 9.2E-01 | 68.47 | 3.7E-02 | 72.88 |
| 7.6E-01 | 68.59 | 2.6E-02 | 72.92 |
| 5.7E-01 | 69.78 | 1.9E-02 | 73.53 |
| 3.8E-01 | 70.09 | 1.0E-02 | 72.98 |
| 1.9E-01 | 72.00 | 5.8E-03 | 73.54 |
| 1.2E-01 | 71.63 | 1.1E-03 | 73.25 |
| 7.6E-02 | 72.94 | 0.0E+00 | 73.87 |
| 5.7E-02 | 72.71 |  |  |

**Table S5.18: TritonX100 + Oxalic acid mixtures (x = 0.1)**

| ∝ Oxalic acid | ∝ TritonX100 | [Oxalic acid] (M) | [TritonX100] (M) | σ (mN/m) |
| --- | --- | --- | --- | --- |
| 1.43E-02 | 1.56E-03 | 7.4E-01 | 8.1E-02 | 31.43 |
| 1.06E-02 | 1.15E-03 | 5.9E-01 | 6.4E-02 | 31.87 |
| 5.09E-03 | 5.53E-04 | 2.8E-01 | 3.1E-02 | 32.16 |
| 2.53E-03 | 2.75E-04 | 1.4E-01 | 1.5E-02 | 32.35 |
| 1.25E-03 | 1.36E-04 | 6.9E-02 | 7.6E-03 | 32.45 |
| 6.24E-04 | 6.81E-05 | 3.5E-02 | 3.8E-03 | 32.43 |
| 3.14E-04 | 3.43E-05 | 1.7E-02 | 1.9E-03 | 32.08 |
| 1.57E-04 | 1.71E-05 | 8.7E-03 | 9.5E-04 | 32.07 |
| 7.89E-05 | 8.62E-06 | 4.4E-03 | 4.8E-04 | 31.83 |
| 3.99E-05 | 4.36E-06 | 2.2E-03 | 2.4E-04 | 33.50 |
| 2.01E-05 | 2.20E-06 | 1.1E-03 | 1.2E-04 | 41.80 |
| 9.80E-06 | 1.07E-06 | 5.4E-04 | 5.9E-05 | 47.93 |
| 4.92E-06 | 5.37E-07 | 2.7E-04 | 3.0E-05 | 56.80 |
| 2.45E-06 | 2.68E-07 | 1.4E-04 | 1.5E-05 | 65.28 |
| 6.20E-07 | 6.77E-08 | 3.4E-05 | 3.8E-06 | 73.01 |
| 0.00E+00 | 0.00E+00 | 0.0E+00 | 0.0E+00 | 73.58 |

**Table S5.19: TritonX100 + Oxalic acid mixtures (x = 0.01)**

| ∝ Oxalic acid | ∝ TritonX100 | [Oxalic acid] (M) | [TritonX100] (M) | σ (mN/m) |
| --- | --- | --- | --- | --- |
| 1.58E-02 | 1.73E-04 | 8.5E-01 | 9.0E-03 | 31.68 |
| 1.19E-02 | 1.28E-04 | 6.6E-01 | 7.2E-03 | 31.92 |
| 5.70E-03 | 6.21E-05 | 3.2E-01 | 3.5E-03 | 32.08 |
| 2.81E-03 | 3.08E-05 | 1.6E-01 | 1.7E-03 | 31.97 |
| 1.40E-03 | 1.53E-05 | 7.8E-02 | 8.5E-04 | 31.92 |
| 6.74E-04 | 7.38E-06 | 3.7E-02 | 4.1E-04 | 31.70 |
| 3.39E-04 | 3.71E-06 | 1.9E-02 | 2.1E-04 | 35.44 |
| 1.68E-04 | 1.84E-06 | 9.3E-03 | 1.0E-04 | 43.46 |
| 8.24E-05 | 9.04E-07 | 4.6E-03 | 5.0E-05 | 49.51 |
| 4.10E-05 | 4.50E-07 | 2.3E-03 | 2.5E-05 | 57.87 |
| 2.05E-05 | 2.25E-07 | 1.1E-03 | 1.3E-05 | 66.23 |
| 1.01E-05 | 1.11E-07 | 5.6E-04 | 6.2E-06 | 73.57 |
| 4.98E-06 | 5.46E-08 | 2.8E-04 | 3.0E-06 | 73.16 |
| 2.49E-06 | 2.73E-08 | 1.4E-04 | 1.5E-06 | 73.53 |
| 0.00E+00 | 0.00E+00 | 0.0E+00 | 0.0E+00 | 73.58 |

**Table S5.20: TritonX100 + Oxalic acid mixtures (x = 0.0011)**

| ∝ Oxalic acid | ∝ TritonX100 | [Oxalic acid] (M) | [TritonX100] (M) | σ (mN/m) |
| --- | --- | --- | --- | --- |
| 1.55E-02 | 1.67E-05 | 8.4E-01 | 9.0E-04 | 31.23 |
| 1.15E-02 | 1.22E-05 | 6.4E-01 | 6.9E-04 | 31.58 |
| 5.57E-03 | 5.96E-06 | 3.1E-01 | 3.3E-04 | 32.21 |
| 2.78E-03 | 2.98E-06 | 1.5E-01 | 1.7E-04 | 38.11 |
| 1.32E-03 | 1.42E-06 | 7.3E-02 | 7.9E-05 | 44.73 |
| 6.65E-04 | 7.16E-07 | 3.7E-02 | 4.0E-05 | 52.21 |
| 3.32E-04 | 3.57E-07 | 1.8E-02 | 2.0E-05 | 59.85 |
| 1.67E-04 | 1.80E-07 | 9.3E-03 | 1.0E-05 | 70.48 |
| 8.27E-05 | 8.90E-08 | 4.6E-03 | 4.9E-06 | 73.00 |
| 4.14E-05 | 4.46E-08 | 2.3E-03 | 2.5E-06 | 73.06 |
| 2.07E-05 | 2.23E-08 | 1.2E-03 | 1.2E-06 | 73.55 |
| 1.03E-05 | 1.11E-08 | 5.7E-04 | 6.2E-07 | 73.18 |
| 0.00E+00 | 0.00E+00 | 0.0E+00 | 0.0E+00 | 73.58 |

**Table S5.21: TritonX100 + Oxalic acid mixtures (x = 0.00047)**

| ∝ Oxalic acid | ∝ TritonX100 | [Oxalic acid] (M) | [TritonX100] (M) | σ (mN/m) |
| --- | --- | --- | --- | --- |
| 1.57E-02 | 7.41E-06 | 8.5E-01 | 4.0E-04 | 31.38 |
| 7.55E-03 | 3.55E-06 | 4.2E-01 | 2.0E-04 | 36.74 |
| 3.74E-03 | 1.76E-06 | 2.1E-01 | 9.8E-05 | 42.87 |
| 1.85E-03 | 8.73E-07 | 1.0E-01 | 4.9E-05 | 49.25 |
| 9.06E-04 | 4.28E-07 | 5.0E-02 | 2.4E-05 | 57.58 |
| 4.54E-04 | 2.15E-07 | 2.5E-02 | 1.2E-05 | 67.10 |
| 2.30E-04 | 1.09E-07 | 1.3E-02 | 6.0E-06 | 72.54 |
| 1.14E-04 | 5.40E-08 | 6.3E-03 | 3.0E-06 | 73.00 |
| 5.74E-05 | 2.71E-08 | 3.2E-03 | 1.5E-06 | 73.48 |
| 2.88E-05 | 1.36E-08 | 1.6E-03 | 7.6E-07 | 73.43 |
| 1.46E-05 | 6.88E-09 | 8.1E-04 | 3.8E-07 | 73.51 |
| 0.00E+00 | 0.00E+00 | 0.0E+00 | 0.0E+00 | 73.59 |

**Table S5.22: TritonX100 + Oxalic acid mixtures (x = 0.00013)**

| ∝ Oxalic acid | ∝ TritonX100 | [Oxalic acid] (M) | [TritonX100] (M) | σ (mN/m) |
| --- | --- | --- | --- | --- |
| 1.56E-02 | 2.01E-06 | 8.4E-01 | 1.1E-04 | 41.03 |
| 7.63E-03 | 9.77E-07 | 4.2E-01 | 5.5E-05 | 46.13 |
| 5.60E-03 | 7.18E-07 | 3.1E-01 | 4.0E-05 | 50.88 |
| 2.69E-03 | 3.45E-07 | 1.5E-01 | 1.9E-05 | 58.53 |
| 2.00E-03 | 2.57E-07 | 1.1E-01 | 1.4E-05 | 61.80 |
| 9.83E-04 | 1.27E-07 | 5.5E-02 | 7.0E-06 | 72.03 |
| 4.88E-04 | 6.29E-08 | 2.7E-02 | 3.5E-06 | 72.81 |
| 2.30E-04 | 2.96E-08 | 1.3E-02 | 1.6E-06 | 73.14 |
| 1.10E-04 | 1.42E-08 | 6.1E-03 | 7.9E-07 | 73.21 |
| 5.42E-05 | 6.99E-09 | 3.0E-03 | 3.9E-07 | 73.51 |
| 2.64E-05 | 3.41E-09 | 1.5E-03 | 1.9E-07 | 73.22 |
| 0.00E+00 | 0.00E+00 | 0.0E+00 | 0.0E+00 | 73.57 |


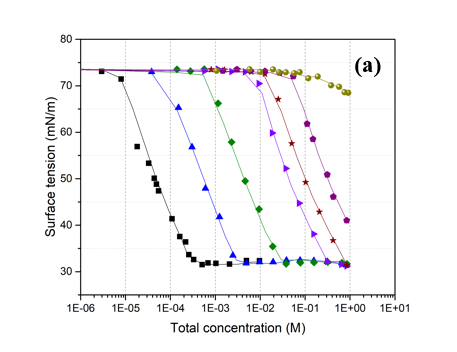


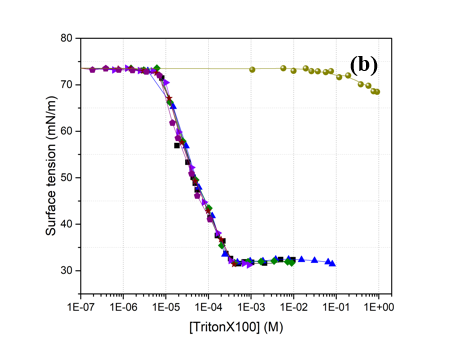


**Figure S14**: **Adsorption isotherms for TritonX100 + oxalic acid mixtures**: **(a) function of the total concentration ([TritonX100] + [Oxalic acid]) and (b) function of the concentration of TritonX100.**  (
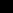
) black curve: TritonX100; (
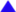
) blue curve: x = 0.1; (
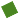
) green curve: x = 0.01; (
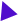
) violet curve: x = 0.0011; (
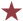
) wine curve: x = 0.00047; (
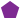
) purple curve: x = 0.00013 and (
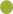
) yellow curve: Oxalic acid.

### **Brij35 + Oxalic acid**

**Table S5.23: Brij35 + Oxalic acid mixtures (x = 0.091)**

| ∝ Oxalic acid | ∝ Brij35 | [Oxalic acid] (M) | [Brij35] (M) | σ (mN/m) |
| --- | --- | --- | --- | --- |
| 4.33E-03 | 4.51E-04 | 2.3E-01 | 2.4E-02 | 43.50 |
| 2.09E-03 | 2.17E-04 | 1.2E-01 | 1.2E-02 | 44.34 |
| 1.04E-03 | 1.08E-04 | 5.8E-02 | 6.0E-03 | 44.44 |
| 5.17E-04 | 5.38E-05 | 2.9E-02 | 3.0E-03 | 44.67 |
| 2.58E-04 | 2.68E-05 | 1.4E-02 | 1.5E-03 | 43.88 |
| 1.28E-04 | 1.33E-05 | 7.1E-03 | 7.4E-04 | 44.29 |
| 6.48E-05 | 6.74E-06 | 3.6E-03 | 3.7E-04 | 44.18 |
| 1.58E-05 | 1.64E-06 | 8.8E-04 | 9.1E-05 | 47.07 |
| 3.92E-06 | 4.07E-07 | 2.2E-04 | 2.3E-05 | 55.93 |
| 1.96E-06 | 2.04E-07 | 1.1E-04 | 1.1E-05 | 59.51 |
| 9.81E-07 | 1.02E-07 | 5.5E-05 | 5.7E-06 | 67.59 |
| 2.48E-07 | 2.58E-08 | 1.4E-05 | 1.4E-06 | 72.53 |
| 6.29E-08 | 6.54E-09 | 3.5E-06 | 3.6E-07 | 72.15 |
| 0.00E+00 | 0.00E+00 | 0.0E+00 | 0.0E+00 | 73.28 |

**Table S5.24: Brij35 + Oxalic acid mixtures (x = 0.0099)**

| ∝ Oxalic acid | ∝ Brij35 | [Oxalic acid] (M) | [Brij35] (M) | σ (mN/m) |
| --- | --- | --- | --- | --- |
| 1.32E-02 | 1.33E-04 | 7.1E-01 | 7.2E-03 | 42.72 |
| 9.72E-03 | 9.69E-05 | 5.4E-01 | 5.4E-03 | 43.08 |
| 7.19E-03 | 7.19E-05 | 4.0E-01 | 4.0E-03 | 43.34 |
| 5.37E-03 | 5.37E-05 | 3.0E-01 | 3.0E-03 | 43.81 |
| 2.66E-03 | 2.67E-05 | 1.5E-01 | 1.5E-03 | 44.10 |
| 1.32E-03 | 1.33E-05 | 7.3E-02 | 7.4E-04 | 44.50 |
| 6.58E-04 | 6.62E-06 | 3.7E-02 | 3.7E-04 | 44.58 |
| 1.63E-04 | 1.64E-06 | 9.0E-03 | 9.1E-05 | 48.06 |
| 4.09E-05 | 4.12E-07 | 2.3E-03 | 2.3E-05 | 55.44 |
| 2.04E-05 | 2.05E-07 | 1.1E-03 | 1.1E-05 | 59.85 |
| 1.03E-05 | 1.04E-07 | 5.7E-04 | 5.8E-06 | 68.54 |
| 2.13E-06 | 2.14E-08 | 1.2E-04 | 1.2E-06 | 73.12 |
| 1.07E-06 | 1.07E-08 | 5.9E-05 | 6.0E-07 | 73.30 |
| 0.00E+00 | 0.00E+00 | 0.0E+00 | 0.0E+00 | 73.27 |

**Table S5.25: Brij35 + Oxalic acid mixtures (x = 0.00111)**

| ∝ Oxalic acid | ∝ Brij35 | [Oxalic acid] (M) | [Brij35] (M) | σ (mN/m) |
| --- | --- | --- | --- | --- |
| 1.67E-02 | 1.84E-05 | 9.0E-01 | 1.0E-03 | 42.06 |
| 1.25E-02 | 1.36E-05 | 6.9E-01 | 7.6E-04 | 43.13 |
| 9.19E-03 | 1.01E-05 | 5.1E-01 | 5.6E-04 | 43.48 |
| 4.58E-03 | 5.05E-06 | 2.5E-01 | 2.8E-04 | 44.01 |
| 2.29E-03 | 2.53E-06 | 1.3E-01 | 1.4E-04 | 45.49 |
| 1.14E-03 | 1.26E-06 | 6.3E-02 | 7.0E-05 | 50.28 |
| 5.74E-04 | 6.34E-07 | 3.2E-02 | 3.5E-05 | 53.01 |
| 2.86E-04 | 3.17E-07 | 1.6E-02 | 1.8E-05 | 58.06 |
| 1.43E-04 | 1.58E-07 | 7.9E-03 | 8.8E-06 | 61.50 |
| 7.13E-05 | 7.89E-08 | 4.0E-03 | 4.4E-06 | 69.61 |
| 3.51E-05 | 3.88E-08 | 2.0E-03 | 2.2E-06 | 72.95 |
| 8.74E-06 | 9.66E-09 | 4.9E-04 | 5.4E-07 | 73.37 |
| 0.00E+00 | 0.00E+00 | 0.0E+00 | 0.0E+00 | 73.14 |

**Table S5.26: Brij35 + Oxalic acid mixtures (x = 0.00018)**

| ∝ Oxalic acid | ∝ Brij35 | [Oxalic acid] (M) | [Brij35] (M) | σ (mN/m) |
| --- | --- | --- | --- | --- |
| 1.68E-02 | 3.04E-06 | 9.1E-01 | 1.6E-04 | 42.00 |
| 1.26E-02 | 2.26E-06 | 7.0E-01 | 1.3E-04 | 44.89 |
| 9.30E-03 | 1.67E-06 | 5.2E-01 | 9.4E-05 | 47.21 |
| 6.95E-03 | 1.25E-06 | 3.9E-01 | 7.0E-05 | 49.41 |
| 3.42E-03 | 6.17E-07 | 1.9E-01 | 3.4E-05 | 53.62 |
| 1.70E-03 | 3.08E-07 | 9.5E-02 | 1.7E-05 | 58.51 |
| 8.45E-04 | 1.53E-07 | 4.7E-02 | 8.5E-06 | 63.93 |
| 4.21E-04 | 7.64E-08 | 2.3E-02 | 4.2E-06 | 70.73 |
| 2.11E-04 | 3.82E-08 | 1.2E-02 | 2.1E-06 | 73.18 |
| 1.05E-04 | 1.91E-08 | 5.8E-03 | 1.1E-06 | 72.89 |
| 5.23E-05 | 9.49E-09 | 2.9E-03 | 5.3E-07 | 72.92 |
| 0.00E+00 | 0.00E+00 | 0.0E+00 | 0.0E+00 | 73.14 |


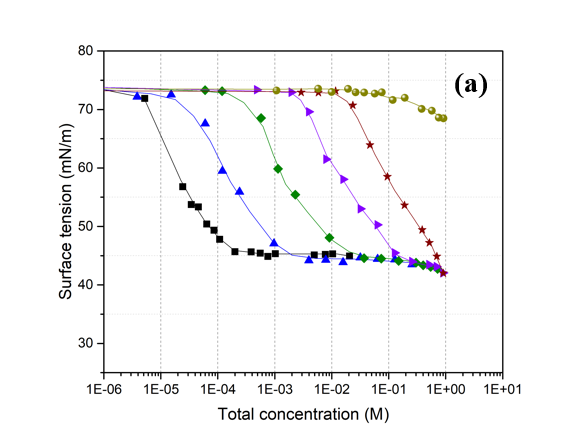

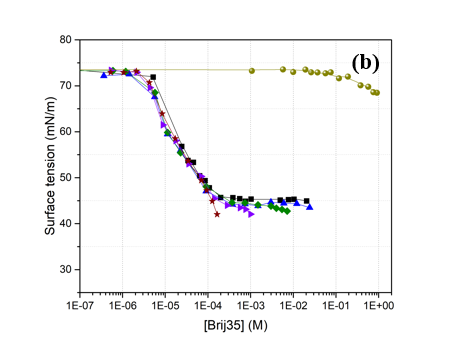


**Figure S15**: **Adsorption isotherms for Brij35 + oxalic acid mixtures****: (a) function of the total concentration ([Brij35] + [Oxalic acid]) and (b) function of the concentration of Brij35.** (
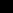
) black curve: Brij35; (
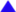
) blue curve: x = 0.091; (
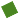
) green curve: x = 0.0099; (
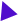
) violet curve: x = 0.00111; (
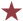
) wine curve: x = 0.00018 and (
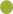
) yellow curve: Oxalic acid.

### **CTAC + Oxalic acid**

**Table S5.27: CTAC solutions (x = 1)**

| [CTAC] (M) | σ (mN/m) | [CTAC] (M) | σ (mN/m) |
| --- | --- | --- | --- |
| 1.0E-01 | 38.82 | 5.4E-04 | 46.95 |
| 5.0E-02 | 39.22 | 2.7E-04 | 58.63 |
| 1.2E-02 | 40.22 | 6.6E-05 | 66.17 |
| 8.9E-03 | 39.72 | 1.5E-05 | 73.08 |
| 4.4E-03 | 39.21 | 3.7E-06 | 73.29 |
| 2.2E-03 | 38.61 | 1.8E-06 | 73.36 |
| 1.1E-03 | 38.71 | 0.0E+00 | 73.365 |

**Table S5.28: CTAC + Oxalic acid mixtures (x = 0.09)**

| ∝ Oxalic acid | ∝ CTAC | [Oxalic acid] (M) | [CTAC] (M) | σ (mN/m) |
| --- | --- | --- | --- | --- |
| 1.68E-02 | 1.68E-03 | 8.8E-01 | 8.9E-02 | 35.39 |
| 8.20E-03 | 8.14E-04 | 4.6E-01 | 4.6E-02 | 36.21 |
| 3.99E-03 | 3.98E-04 | 2.2E-01 | 2.2E-02 | 36.61 |
| 1.99E-03 | 1.99E-04 | 1.1E-01 | 1.1E-02 | 36.92 |
| 9.88E-04 | 9.87E-05 | 5.5E-02 | 5.5E-03 | 36.51 |
| 4.95E-04 | 4.95E-05 | 2.7E-02 | 2.8E-03 | 37.30 |
| 2.47E-04 | 2.47E-05 | 1.4E-02 | 1.4E-03 | 35.19 |
| 1.23E-04 | 1.23E-05 | 6.8E-03 | 6.9E-04 | 35.17 |
| 6.16E-05 | 6.16E-06 | 3.4E-03 | 3.4E-04 | 34.35 |
| 3.08E-05 | 3.08E-06 | 1.7E-03 | 1.7E-04 | 38.94 |
| 1.52E-05 | 1.52E-06 | 8.5E-04 | 8.5E-05 | 47.53 |
| 1.02E-05 | 1.02E-06 | 5.6E-04 | 5.6E-05 | 57.79 |
| 6.77E-06 | 6.77E-07 | 3.8E-04 | 3.8E-05 | 68.75 |
| 3.54E-06 | 3.55E-07 | 2.0E-04 | 2.0E-05 | 73.27 |
| 1.77E-06 | 1.77E-07 | 9.8E-05 | 9.8E-06 | 73.07 |
| 0.00E+00 | 0.00E+00 | 0.0E+00 | 0.0E+00 | 73.41 |

**Table S5.29: CTAC + Oxalic acid mixtures (x = 0.01)**

| ∝ Oxalic acid | ∝ CTAC | [Oxalic acid] (M) | [CTAC] (M) | σ (mN/m) |
| --- | --- | --- | --- | --- |
| 1.83E-02 | 1.83E-04 | 9.9E-01 | 9.9E-03 | 34.45 |
| 8.96E-03 | 8.88E-05 | 5.0E-01 | 5.0E-03 | 35.78 |
| 4.36E-03 | 4.34E-05 | 2.4E-01 | 2.4E-03 | 35.77 |
| 2.16E-03 | 2.15E-05 | 1.2E-01 | 1.2E-03 | 35.80 |
| 1.07E-03 | 1.07E-05 | 5.9E-02 | 5.9E-04 | 35.56 |
| 5.32E-04 | 5.32E-06 | 3.0E-02 | 3.0E-04 | 36.10 |
| 2.63E-04 | 2.63E-06 | 1.5E-02 | 1.5E-04 | 38.13 |
| 1.32E-04 | 1.32E-06 | 7.3E-03 | 7.3E-05 | 47.63 |
| 6.60E-05 | 6.60E-07 | 3.7E-03 | 3.7E-05 | 61.83 |
| 3.28E-05 | 3.27E-07 | 1.8E-03 | 1.8E-05 | 72.25 |
| 1.62E-05 | 1.62E-07 | 9.0E-04 | 9.0E-06 | 72.31 |
| 8.14E-06 | 8.14E-08 | 4.5E-04 | 4.5E-06 | 72.86 |
| 0.00E+00 | 0.00E+00 | 0.0E+00 | 0.0E+00 | 73.40 |

**Table S5.30: CTAC + Oxalic acid mixtures (x = 0.0014)**

| ∝ Oxalic acid | ∝ CTAC | [Oxalic acid] (M) | [CTAC] (M) | σ (mN/m) |
| --- | --- | --- | --- | --- |
| 1.85E-02 | 2.56E-05 | 1.0E+00 | 1.4E-03 | 33.90 |
| 1.38E-02 | 1.88E-05 | 7.7E-01 | 1.1E-03 | 34.55 |
| 1.01E-02 | 1.38E-05 | 5.6E-01 | 7.8E-04 | 35.85 |
| 7.42E-03 | 1.02E-05 | 4.1E-01 | 5.7E-04 | 35.03 |
| 5.47E-03 | 7.54E-06 | 3.0E-01 | 4.2E-04 | 35.39 |
| 4.05E-03 | 5.59E-06 | 2.3E-01 | 3.1E-04 | 35.60 |
| 2.00E-03 | 2.77E-06 | 1.1E-01 | 1.5E-04 | 35.53 |
| 1.01E-03 | 1.40E-06 | 5.6E-02 | 7.8E-05 | 43.15 |
| 5.08E-04 | 7.03E-07 | 2.8E-02 | 3.9E-05 | 60.85 |
| 3.38E-04 | 4.68E-07 | 1.9E-02 | 2.6E-05 | 70.58 |
| 2.55E-04 | 3.53E-07 | 1.4E-02 | 2.0E-05 | 72.07 |
| 1.88E-04 | 2.60E-07 | 1.0E-02 | 1.4E-05 | 73.10 |
| 0.00E+00 | 0.00E+00 | 0.0E+00 | 0.0E+00 | 73.40 |

**Table S5.31: CTAC + Oxalic acid mixtures (x = 0.0001)**

| ∝ Oxalic acid | ∝ CTAC | [Oxalic acid] (M) | [CTAC] (M) | σ (mN/m) |
| --- | --- | --- | --- | --- |
| 1.85E-02 | 1.76E-06 | 1.0E+00 | 9.5E-05 | 35.37 |
| 1.37E-02 | 1.28E-06 | 7.6E-01 | 7.2E-05 | 37.83 |
| 9.95E-03 | 9.34E-07 | 5.5E-01 | 5.2E-05 | 43.59 |
| 7.27E-03 | 6.85E-07 | 4.0E-01 | 3.8E-05 | 50.27 |
| 5.31E-03 | 5.01E-07 | 2.9E-01 | 2.8E-05 | 68.31 |
| 3.92E-03 | 3.70E-07 | 2.2E-01 | 2.1E-05 | 69.61 |
| 2.88E-03 | 2.73E-07 | 1.6E-01 | 1.5E-05 | 71.43 |
| 1.57E-03 | 1.48E-07 | 8.7E-02 | 8.3E-06 | 71.61 |
| 8.69E-04 | 8.24E-08 | 4.8E-02 | 4.6E-06 | 71.87 |
| 6.40E-04 | 6.06E-08 | 3.6E-02 | 3.4E-06 | 72.43 |
| 0.00E+00 | 0.00E+00 | 0.0E+00 | 0.0E+00 | 73.41 |


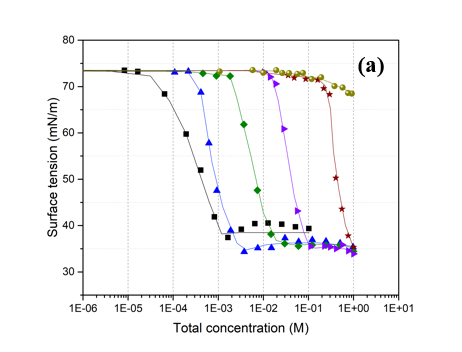

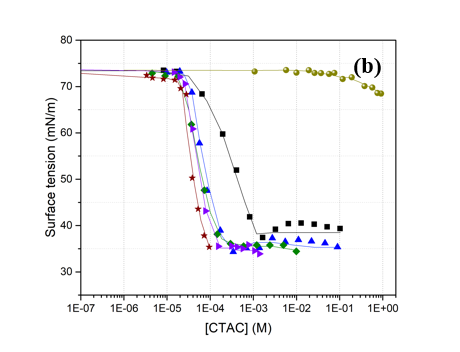


**Figure S16**: **Adsorption isotherms for CTAC + Oxalic acid mixtures**: (a) function of the total concentration ([CTAC] + [Oxalic acid]) and (b) function of the concentration of CTAC. (
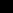
) black curve: CTAC; (
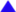
) blue curve: x **=** 0.09; (
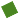
) green curve: x = 0.01; (
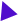
) violet curve: x = 0.0014; (
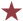
) wine curve: x = 0.0001; (
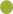
) yellow curve: Oxalic acid.

### **SDS + Oxalic acid**

**Table S5.32: SDS solutions (x = 0)**

| [SDS] (M) | σ (mN/m)) | [SDS] (M) | σ (mN/m) |
| --- | --- | --- | --- |
| 9.6E-02 | 34.06 | 9.9E-04 | 68.22 |
| 7.8E-02 | 34.21 | 7.7E-04 | 69.21 |
| 4.9E-02 | 34.76 | 5.1E-04 | 71.02 |
| 2.1E-02 | 35.18 | 2.7E-04 | 71.85 |
| 1.1E-02 | 34.29 | 8.3E-05 | 72.38 |
| 8.9E-03 | 33.44 | 4.1E-05 | 72.91 |
| 7.0E-03 | 37.58 | 0.0E+00 | 73.38 |
| 2.8E-03 | 55.69 |  |  |

**Table S5.33: SDS + Oxalic acid mixtures (x = 0.09)**

| ∝ Oxalic acid | ∝ SDS | [Oxalic acid] (M) | [SDS] (M) | σ (mN/m) |
| --- | --- | --- | --- | --- |
| 1.22E-02 | 1.22E-03 | 6.5E-01 | 6.5E-02 | 32.40 |
| 5.95E-03 | 5.93E-04 | 3.3E-01 | 3.3E-02 | 33.61 |
| 2.90E-03 | 2.90E-04 | 1.6E-01 | 1.6E-02 | 34.29 |
| 1.43E-03 | 1.43E-04 | 8.0E-02 | 8.0E-03 | 35.01 |
| 7.14E-04 | 7.15E-05 | 4.0E-02 | 4.0E-03 | 32.67 |
| 3.54E-04 | 3.55E-05 | 2.0E-02 | 2.0E-03 | 41.83 |
| 1.77E-04 | 1.78E-05 | 9.8E-03 | 9.9E-04 | 52.90 |
| 4.55E-05 | 4.56E-06 | 2.5E-03 | 2.5E-04 | 66.61 |
| 2.27E-05 | 2.28E-06 | 1.3E-03 | 1.3E-04 | 71.25 |
| 1.70E-05 | 1.70E-06 | 9.4E-04 | 9.4E-05 | 71.32 |
| 1.20E-05 | 1.21E-06 | 6.7E-04 | 6.7E-05 | 71.35 |
| 8.91E-06 | 8.93E-07 | 4.9E-04 | 5.0E-05 | 73.03 |
| 4.39E-06 | 4.40E-07 | 2.4E-04 | 2.4E-05 | 72.00 |
| 0.00E+00 | 0.00E+00 | 0.0E+00 | 0.0E+00 | 73.27 |

**Table S5.34: SDS + Oxalic acid mixtures (x = 0.01)**

| ∝ Oxalic acid | ∝ SDS | [Oxalic acid] (M) | [SDS] (M) | σ (mN/m) |
| --- | --- | --- | --- | --- |
| 1.76E-02 | 1.76E-04 | 9.5E-01 | 9.5E-03 | 30.28 |
| 8.67E-03 | 8.62E-05 | 4.8E-01 | 4.8E-03 | 31.09 |
| 4.22E-03 | 4.21E-05 | 2.3E-01 | 2.3E-03 | 32.63 |
| 2.02E-03 | 2.02E-05 | 1.1E-01 | 1.1E-03 | 40.59 |
| 9.95E-04 | 9.97E-06 | 5.5E-02 | 5.5E-04 | 51.50 |
| 7.45E-04 | 7.46E-06 | 4.1E-02 | 4.1E-04 | 54.75 |
| 5.61E-04 | 5.62E-06 | 3.1E-02 | 3.1E-04 | 58.32 |
| 4.18E-04 | 4.19E-06 | 2.3E-02 | 2.3E-04 | 61.72 |
| 2.08E-04 | 2.08E-06 | 1.2E-02 | 1.2E-04 | 67.94 |
| 1.03E-04 | 1.03E-06 | 5.7E-03 | 5.7E-05 | 70.91 |
| 5.00E-05 | 5.01E-07 | 2.8E-03 | 2.8E-05 | 72.43 |
| 2.47E-05 | 2.48E-07 | 1.4E-03 | 1.4E-05 | 72.95 |
| 0.00E+00 | 0.00E+00 | 0.0E+00 | 0.0E+00 | 73.27 |

**Table S5.35: SDS + Oxalic acid mixtures (x = 0.001)**

| ∝ Oxalic acid | ∝ SDS | [Oxalic acid] (M) | [SDS] (M) | σ (mN/m) |
| --- | --- | --- | --- | --- |
| 1.84E-02 | 1.85E-05 | 9.9E-01 | 1.0E-03 | 28.57 |
| 1.37E-02 | 1.36E-05 | 7.6E-01 | 7.6E-04 | 36.95 |
| 1.01E-02 | 1.00E-05 | 5.6E-01 | 5.6E-04 | 42.55 |
| 7.49E-03 | 7.48E-06 | 4.2E-01 | 4.2E-04 | 46.84 |
| 3.71E-03 | 3.72E-06 | 2.1E-01 | 2.1E-04 | 54.69 |
| 1.80E-03 | 1.81E-06 | 1.0E-01 | 1.0E-04 | 63.91 |
| 1.35E-03 | 1.35E-06 | 7.5E-02 | 7.5E-05 | 66.30 |
| 9.85E-04 | 9.90E-07 | 5.5E-02 | 5.5E-05 | 68.35 |
| 7.20E-04 | 7.24E-07 | 4.0E-02 | 4.0E-05 | 70.10 |
| 5.32E-04 | 5.35E-07 | 3.0E-02 | 3.0E-05 | 70.98 |
| 3.91E-04 | 3.93E-07 | 2.2E-02 | 2.2E-05 | 71.93 |
| 0.00E+00 | 0.00E+00 | 0.0E+00 | 0.0E+00 | 73.27 |

**Table S5.36: SDS + Oxalic acid mixtures (x = 0.0001)**

| ∝ Oxalic acid | ∝ SDS | [Oxalic acid] (M) | [SDS] (M) | σ (mN/m) |
| --- | --- | --- | --- | --- |
| 1.85E-02 | 1.85E-06 | 1.0E+00 | 1.0E-04 | 52.66 |
| 1.35E-02 | 1.33E-06 | 7.5E-01 | 7.5E-05 | 57.57 |
| 7.21E-03 | 7.14E-07 | 4.0E-01 | 4.0E-05 | 66.50 |
| 3.88E-03 | 3.86E-07 | 2.2E-01 | 2.2E-05 | 70.51 |
| 2.86E-03 | 2.84E-07 | 1.6E-01 | 1.6E-05 | 71.77 |
| 2.06E-03 | 2.05E-07 | 1.1E-01 | 1.1E-05 | 71.18 |
| 1.52E-03 | 1.52E-07 | 8.5E-02 | 8.4E-06 | 71.75 |
| 1.12E-03 | 1.11E-07 | 6.2E-02 | 6.2E-06 | 72.95 |
| 8.26E-04 | 8.24E-08 | 4.6E-02 | 4.6E-06 | 72.81 |
| 0.00E+00 | 0.00E+00 | 0.0E+00 | 0.0E+00 | 73.27 |


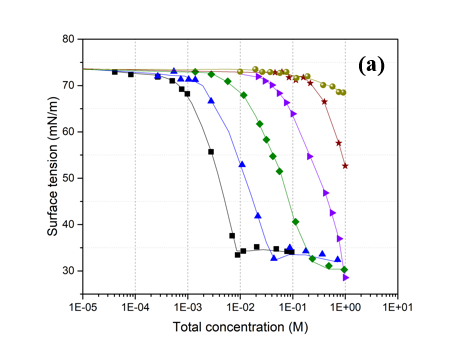

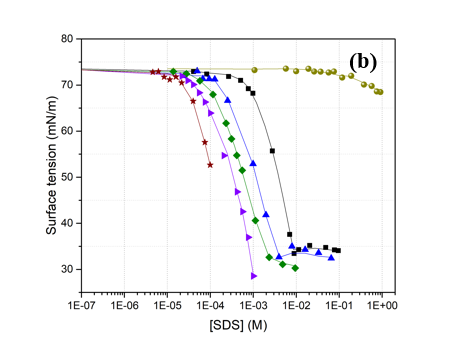


**Figure S17**: **Adsorption isotherms for SDS + Oxalic acid mixtures**: (a) function of the total concentration ([SDS] + [Oxalic acid]) and (b) function of the concentration of SDS (
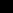
) black curve: SDS; (
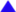
) blue curve: x **=** 0.09; (
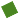
) green curve: x = 0.01; () violet curve: x = 0.001; () wine curve: x = 0.0001; () yellow curve: Oxalic acid.

### **Effects of organic acids on the CMC of surfactants**

**Figure S18:** **Evolution of the CMC for surfactant + organic acid mixtures.** (a) () TritonX100 + Glutaric acid and () TritonX100 + Oxalic acid (with x_Triton_= molar fraction of TritonX100). (b) () Brij35 + Glutaric acid and () Brij35 + Oxalic acid (x_Brij_ = molar fraction of Brij35). (c) () CTAC + Oxalic acid (x_CTAC_ = molar fraction of CTAC) and (d) () SDS + Oxalic acid (x_SDS_ = molar fraction of SDS).

# **S6. Adsorption isotherms for mixtures of two amphiphilic surfactants**

### **SDS + CTAC**

*With α = molar fraction of compounds in water and x the organic molar fraction of SDS.*

**Table S6.1: SDS + CTAC mixtures (x = 0.33)**

| ∝ SDS | ∝ CTAC | [SDS] (M) | [CTAC] (M) | σ (mN/m) |
| --- | --- | --- | --- | --- |
| 8.47E-04 | 1.97E-03 | 4.5E-02 | 1.0E-01 | 31.53 |
| 4.13E-04 | 9.61E-04 | 2.3E-02 | 5.3E-02 | 32.88 |
| 1.99E-04 | 4.64E-04 | 1.1E-02 | 2.6E-02 | 34.03 |
| 9.74E-05 | 2.27E-04 | 5.4E-03 | 1.3E-02 | 35.17 |
| 4.83E-05 | 1.13E-04 | 2.7E-03 | 6.3E-03 | 35.94 |
| 2.38E-05 | 5.53E-05 | 1.3E-03 | 3.1E-03 | 37.35 |
| 1.18E-05 | 2.76E-05 | 6.6E-04 | 1.5E-03 | 41.70 |
| 2.88E-06 | 6.71E-06 | 1.6E-04 | 3.7E-04 | 54.30 |
| 1.43E-06 | 3.32E-06 | 7.9E-05 | 1.8E-04 | 63.31 |
| 7.09E-07 | 1.65E-06 | 3.9E-05 | 9.2E-05 | 66.92 |
| 3.53E-07 | 8.22E-07 | 2.0E-05 | 4.6E-05 | 71.18 |
| 1.71E-07 | 3.99E-07 | 9.5E-06 | 2.2E-05 | 72.53 |
| 8.36E-08 | 1.95E-07 | 4.6E-06 | 1.1E-05 | 72.78 |
| 0.00E+00 | 0.00E+00 | 0.0E+00 | 0.0E+00 | 73.17 |

**Table S6.2: SDS + CTAC mixtures** **(x = 0.5)**

| ∝ SDS | ∝ CTAC | [SDS] (M) | [CTAC] (M) | σ (mN/m) |
| --- | --- | --- | --- | --- |
| 1.81E-03 | 1.98E-03 | 9.5E-02 | 1.0E-01 | 30.87 |
| 4.30E-04 | 4.78E-04 | 2.4E-02 | 2.6E-02 | 34.99 |
| 2.76E-04 | 3.08E-04 | 1.5E-02 | 1.7E-02 | 36.07 |
| 9.47E-05 | 1.07E-04 | 5.3E-03 | 5.9E-03 | 38.10 |
| 4.28E-05 | 4.85E-05 | 2.4E-03 | 2.7E-03 | 43.54 |
| 2.82E-05 | 3.12E-05 | 1.6E-03 | 1.7E-03 | 45.97 |
| 1.39E-05 | 1.54E-05 | 7.7E-04 | 8.6E-04 | 54.03 |
| 6.99E-06 | 7.73E-06 | 3.9E-04 | 4.3E-04 | 64.63 |
| 3.44E-06 | 3.81E-06 | 1.9E-04 | 2.1E-04 | 71.50 |
| 1.71E-06 | 1.89E-06 | 9.5E-05 | 1.1E-04 | 71.93 |
| 8.52E-07 | 9.43E-07 | 4.7E-05 | 5.2E-05 | 73.00 |
| 4.26E-07 | 4.72E-07 | 2.4E-05 | 2.6E-05 | 72.83 |
| 2.14E-07 | 2.37E-07 | 1.2E-05 | 1.3E-05 | 72.75 |
| 1.07E-07 | 1.19E-07 | 6.0E-06 | 6.6E-06 | 73.18 |
| 5.20E-08 | 5.75E-08 | 2.9E-06 | 3.2E-06 | 73.19 |
| 0.00E+00 | 0.00E+00 | 0.0E+00 | 0.0E+00 | 73.76 |

**Table S6.3: SDS + CTAC mixtures** **(x = 0.66)**

| ∝ SDS | ∝ CTAC | [SDS] (M) | [CTAC] (M) | σ (mN/m) |
| --- | --- | --- | --- | --- |
| 1.73E-03 | 9.48E-04 | 9.2E-02 | 5.1E-02 | 33.17 |
| 8.46E-04 | 4.64E-04 | 4.7E-02 | 2.6E-02 | 34.61 |
| 4.17E-04 | 2.29E-04 | 2.3E-02 | 1.3E-02 | 35.31 |
| 2.08E-04 | 1.14E-04 | 1.2E-02 | 6.4E-03 | 39.13 |
| 1.04E-04 | 5.73E-05 | 5.8E-03 | 3.2E-03 | 47.10 |
| 5.15E-05 | 2.83E-05 | 2.9E-03 | 1.6E-03 | 56.70 |
| 1.27E-05 | 6.99E-06 | 7.1E-04 | 3.9E-04 | 68.20 |
| 6.35E-06 | 3.48E-06 | 3.5E-04 | 1.9E-04 | 69.53 |
| 3.18E-06 | 1.74E-06 | 1.8E-04 | 9.7E-05 | 71.40 |
| 1.60E-06 | 8.79E-07 | 8.9E-05 | 4.9E-05 | 71.87 |
| 7.90E-07 | 4.33E-07 | 4.4E-05 | 2.4E-05 | 72.22 |
| 3.93E-07 | 2.15E-07 | 2.2E-05 | 1.2E-05 | 72.54 |
| 1.96E-07 | 1.08E-07 | 1.1E-05 | 6.0E-06 | 72.82 |
| 0.00E+00 | 0.00E+00 | 0.0E+00 | 0.0E+00 | 73.17 |

**Figure S19**: **Adsorption isotherms for SDS + CTAC mixtures**: **(a) function of the total concentration ([CTAC] + [SDS]) and (b) function of the concentration of SDS.**  () black curve: SDS; () blue curve: x = 0.66; () green curve: x = 0.5; () violet curve: x = 0.33 and () wine curve: CTAC.

### **TritonX114 + SDS**

*With α = molar fraction of compounds in water and x the organic molar fraction of TritonX114.*

**Table S6.4: TritonX114 + SDS mixtures (x = 0.1)**

| ∝ SDS | ∝ TritonX114 | [SDS] (M) | [TritonX114] (M) | σ (mN/m) |
| --- | --- | --- | --- | --- |
| 1.06E-03 | 1.22E-04 | 5.8E-02 | 6.60E-03 | 32.34 |
| 5.22E-04 | 6.00E-05 | 2.9E-02 | 3.30E-03 | 33.06 |
| 1.74E-04 | 2.01E-05 | 9.7E-03 | 1.10E-03 | 32.69 |
| 1.39E-04 | 1.60E-05 | 7.7E-03 | 8.90E-04 | 32.84 |
| 1.03E-04 | 1.19E-05 | 5.7E-03 | 6.60E-04 | 32.73 |
| 5.18E-05 | 5.96E-06 | 2.9E-03 | 3.30E-04 | 32.61 |
| 1.75E-05 | 2.01E-06 | 9.7E-04 | 1.10E-04 | 33.99 |
| 1.39E-05 | 1.60E-06 | 7.7E-04 | 8.90E-05 | 37.20 |
| 1.03E-05 | 1.19E-06 | 5.7E-04 | 6.60E-05 | 41.62 |
| 5.28E-06 | 6.08E-07 | 2.9E-04 | 3.40E-05 | 49.60 |
| 1.77E-06 | 2.04E-07 | 9.9E-05 | 1.10E-05 | 68.38 |
| 1.42E-06 | 1.63E-07 | 7.9E-05 | 9.10E-06 | 70.24 |
| 1.03E-06 | 1.18E-07 | 5.7E-05 | 6.60E-06 | 72.31 |
| 5.52E-07 | 6.35E-08 | 3.1E-05 | 3.50E-06 | 72.90 |
| 1.65E-07 | 1.90E-08 | 9.2E-06 | 1.10E-06 | 73.26 |
| 0.00E+00 | 0.00E+00 | 0.0E+00 | 0.00E+00 | 73.69 |

**Table S6.5: TritonX114 + SDS mixtures (x = 0.15)**

| ∝ SDS | ∝ TritonX114 | [SDS] (M) | [TritonX114] (M) | σ (mN/m) |
| --- | --- | --- | --- | --- |
| 1.07E-03 | 2.20E-04 | 5.8E-02 | 1.2E-02 | 31.93 |
| 5.28E-04 | 1.09E-04 | 2.9E-02 | 6.0E-03 | 32.69 |
| 1.75E-04 | 3.62E-05 | 9.7E-03 | 2.0E-03 | 32.99 |
| 1.39E-04 | 2.87E-05 | 7.7E-03 | 1.6E-03 | 33.17 |
| 1.08E-04 | 2.22E-05 | 6.0E-03 | 1.2E-03 | 33.44 |
| 5.25E-05 | 1.08E-05 | 2.9E-03 | 6.0E-04 | 32.74 |
| 1.73E-05 | 3.57E-06 | 9.6E-04 | 2.0E-04 | 32.19 |
| 1.42E-05 | 2.93E-06 | 7.9E-04 | 1.6E-04 | 32.73 |
| 1.08E-05 | 2.22E-06 | 6.0E-04 | 1.2E-04 | 35.72 |
| 4.83E-06 | 9.96E-07 | 2.7E-04 | 5.5E-05 | 45.12 |
| 1.71E-06 | 3.52E-07 | 9.5E-05 | 2.0E-05 | 57.09 |
| 4.95E-07 | 1.02E-07 | 2.8E-05 | 5.7E-06 | 72.57 |
| 1.74E-07 | 3.59E-08 | 9.7E-06 | 2.0E-06 | 73.06 |
| 0.00E+00 | 0.00E+00 | 0.0E+00 | 0.0E+00 | 73.69 |

**Table S6.6: TritonX114 + SDS mixtures (x = 0.33)**

| ∝ SDS | ∝ TritonX114 | [SDS] (M) | [TritonX114] (M) | σ (mN/m) |
| --- | --- | --- | --- | --- |
| 1.08E-03 | 5.27E-04 | 5.8E-02 | 2.8E-02 | 30.92 |
| 6.43E-04 | 3.13E-04 | 3.6E-02 | 1.7E-02 | 31.71 |
| 2.19E-04 | 1.07E-04 | 1.2E-02 | 5.9E-03 | 32.43 |
| 1.52E-04 | 7.42E-05 | 8.5E-03 | 4.1E-03 | 32.68 |
| 1.05E-04 | 5.13E-05 | 5.8E-03 | 2.9E-03 | 32.75 |
| 6.32E-05 | 3.08E-05 | 3.5E-03 | 1.7E-03 | 32.37 |
| 2.17E-05 | 1.06E-05 | 1.2E-03 | 5.9E-04 | 32.03 |
| 1.55E-05 | 7.57E-06 | 8.6E-04 | 4.2E-04 | 31.82 |
| 1.06E-05 | 5.16E-06 | 5.9E-04 | 2.9E-04 | 31.48 |
| 6.37E-06 | 3.11E-06 | 3.5E-04 | 1.7E-04 | 32.46 |
| 2.18E-06 | 1.06E-06 | 1.2E-04 | 5.9E-05 | 44.04 |
| 1.59E-06 | 7.76E-07 | 8.8E-05 | 4.3E-05 | 47.76 |
| 1.07E-06 | 5.20E-07 | 5.9E-05 | 2.9E-05 | 52.99 |
| 2.19E-07 | 1.07E-07 | 1.2E-05 | 5.9E-06 | 72.82 |
| 1.10E-07 | 5.35E-08 | 6.1E-06 | 3.0E-06 | 73.44 |
| 0.00E+00 | 0.00E+00 | 0.0E+00 | 0.0E+00 | 73.99 |

**Table S6.7: TritonX114 + SDS mixtures (x = 0.5)**

| ∝ SDS | ∝ TritonX114 | [SDS] (M) | [TritonX114] (M) | σ (mN/m) |
| --- | --- | --- | --- | --- |
| 8.98E-04 | 8.74E-04 | 4.8E-02 | 4.7E-02 | 30.52 |
| 5.31E-04 | 5.17E-04 | 3.0E-02 | 2.9E-02 | 30.87 |
| 1.75E-04 | 1.71E-04 | 9.7E-03 | 9.5E-03 | 31.32 |
| 1.04E-04 | 1.01E-04 | 5.8E-03 | 5.6E-03 | 31.73 |
| 8.67E-05 | 8.44E-05 | 4.8E-03 | 4.7E-03 | 31.98 |
| 5.19E-05 | 5.05E-05 | 2.9E-03 | 2.8E-03 | 31.78 |
| 1.72E-05 | 1.67E-05 | 9.5E-04 | 9.3E-04 | 31.65 |
| 1.03E-05 | 1.00E-05 | 5.7E-04 | 5.6E-04 | 31.36 |
| 8.56E-06 | 8.34E-06 | 4.8E-04 | 4.6E-04 | 31.28 |
| 5.12E-06 | 4.99E-06 | 2.8E-04 | 2.8E-04 | 31.30 |
| 1.69E-06 | 1.65E-06 | 9.4E-05 | 9.2E-05 | 40.89 |
| 1.03E-06 | 1.00E-06 | 5.7E-05 | 5.6E-05 | 44.90 |
| 8.44E-07 | 8.21E-07 | 4.7E-05 | 4.6E-05 | 49.30 |
| 1.68E-07 | 1.63E-07 | 9.3E-06 | 9.1E-06 | 71.13 |
| 8.53E-08 | 8.30E-08 | 4.7E-06 | 4.6E-06 | 72.07 |
| 0.00E+00 | 0.00E+00 | 0.0E+00 | 0.0E+00 | 73.34 |

**Table S6.8: TritonX114 + SDS mixtures (x = 0.66)**

| ∝ SDS | ∝ TritonX114 | [SDS] (M) | [TritonX114] (M) | σ (mN/m) |
| --- | --- | --- | --- | --- |
| 5.43E-04 | 1.06E-03 | 2.9E-02 | 5.7E-02 | 30.26 |
| 3.22E-04 | 6.26E-04 | 1.8E-02 | 3.5E-02 | 30.67 |
| 1.06E-04 | 2.06E-04 | 5.9E-03 | 1.1E-02 | 31.02 |
| 7.30E-05 | 1.42E-04 | 4.1E-03 | 7.9E-03 | 31.24 |
| 5.23E-05 | 1.02E-04 | 2.9E-03 | 5.6E-03 | 31.43 |
| 3.18E-05 | 6.19E-05 | 1.8E-03 | 3.4E-03 | 31.32 |
| 1.06E-05 | 2.06E-05 | 5.9E-04 | 1.1E-03 | 31.04 |
| 7.26E-06 | 1.41E-05 | 4.0E-04 | 7.8E-04 | 31.19 |
| 5.26E-06 | 1.02E-05 | 2.9E-04 | 5.7E-04 | 31.06 |
| 3.22E-06 | 6.26E-06 | 1.8E-04 | 3.5E-04 | 30.77 |
| 1.05E-06 | 2.04E-06 | 5.8E-05 | 1.1E-04 | 38.15 |
| 7.31E-07 | 1.42E-06 | 4.1E-05 | 7.9E-05 | 41.74 |
| 5.24E-07 | 1.02E-06 | 2.9E-05 | 5.7E-05 | 45.49 |
| 1.07E-07 | 2.09E-07 | 6.0E-06 | 1.2E-05 | 68.59 |
| 5.34E-08 | 1.04E-07 | 3.0E-06 | 5.8E-06 | 73.45 |
| 0.00E+00 | 0.00E+00 | 0.0E+00 | 0.0E+00 | 73.51 |

**Figure S20**: **Adsorption isotherm for SDS + TritonX114 mixtures**: **(a) function of the total concentration ([TritonX114] + [SDS]) and (b) function of the concentration of SDS.**  () black curve: TritonX114; () blue curve: x = 0.66; () green curve: x = 0.5; () violet curve: x = 0.33; () wine curve: x = 0.15; () yellow curve: x = 0.1 and () red curve: SDS.

# **S7. Adsorption isotherms for two organic acids mixtures**

### **Glutaric acid + Oxalic acid**

*With α = molar fraction of compounds in water and x the organic molar fraction of glutaric acid.*

**Table S7.1: Glutaric acid + Oxalic acid mixtures (x = 0.25)**

| ∝ Glutaric acid | ∝ Oxalic acid | [Glutaric acid] (M) | [Oxalic acid] (M) | σ (mN/m) |
| --- | --- | --- | --- | --- |
| 5.15E-03 | 1.44E-02 | 2.7E-01 | 8.0E-01 | 64.34 |
| 3.69E-03 | 1.07E-02 | 2.0E-01 | 6.0E-01 | 66.65 |
| 2.67E-03 | 7.03E-03 | 1.4E-01 | 4.0E-01 | 68.31 |
| 1.81E-03 | 5.10E-03 | 1.0E-01 | 3.0E-01 | 69.29 |
| 1.26E-03 | 3.42E-03 | 6.9E-02 | 2.0E-01 | 70.39 |
| 9.36E-04 | 2.66E-03 | 5.0E-02 | 1.5E-01 | 70.82 |
| 7.42E-04 | 2.00E-03 | 4.0E-02 | 1.1E-01 | 71.34 |
| 5.00E-04 | 1.36E-03 | 2.7E-02 | 7.5E-02 | 72.23 |
| 3.58E-04 | 9.83E-04 | 2.0E-02 | 6.0E-02 | 72.43 |
| 2.21E-04 | 6.62E-04 | 1.2E-02 | 3.6E-02 | 72.63 |
| 1.17E-04 | 3.32E-04 | 6.5E-03 | 1.8E-02 | 72.93 |
| 7.32E-05 | 1.92E-04 | 4.0E-03 | 1.1E-02 | 72.88 |
| 4.47E-05 | 1.49E-04 | 2.5E-03 | 8.0E-03 | 73.08 |
| 0.00E+00 | 0.00E+00 | 0.0E+00 | 0.0E+00 | 73.29 |

**Table S7.2: Glutaric acid + Oxalic acid mixtures (x = 0.4)**

| ∝ Glutaric acid | ∝ Oxalic acid | [Glutaric acid] (M) | [Oxalic acid] (M) | σ (mN/m) |
| --- | --- | --- | --- | --- |
| 9.89E-03 | 1.37E-02 | 5.2E-01 | 7.2E-01 | 62.36 |
| 7.72E-03 | 1.08E-02 | 4.1E-01 | 5.7E-01 | 63.70 |
| 4.78E-03 | 7.24E-03 | 2.6E-01 | 3.9E-01 | 66.07 |
| 2.55E-03 | 3.44E-03 | 1.4E-01 | 1.9E-01 | 68.97 |
| 1.20E-03 | 1.74E-03 | 6.6E-02 | 9.6E-02 | 70.74 |
| 1.03E-03 | 1.33E-03 | 5.7E-02 | 7.3E-02 | 71.48 |
| 5.06E-04 | 7.09E-04 | 2.8E-02 | 3.9E-02 | 72.37 |
| 2.93E-04 | 3.42E-04 | 1.6E-02 | 1.9E-02 | 71.90 |
| 1.33E-04 | 1.87E-04 | 7.4E-03 | 1.0E-02 | 71.97 |
| 1.25E-04 | 1.76E-04 | 7.0E-03 | 9.8E-03 | 73.15 |
| 6.59E-05 | 1.17E-04 | 3.7E-03 | 6.5E-03 | 73.31 |
| 5.83E-05 | 8.35E-05 | 3.2E-03 | 4.6E-03 | 73.44 |
| 0.00E+00 | 0.00E+00 | 0.0E+00 | 0.0E+00 | 73.29 |

**Table S7.3: Glutaric acid + Oxalic acid mixtures** **(x = 0.57)**

| ∝ Glutaric acid | ∝ Oxalic acid | [Glutaric acid] (M) | [Oxalic acid] (M) | σ (mN/m) |
| --- | --- | --- | --- | --- |
| 1.56E-02 | 1.15E-02 | 8.0E-01 | 6.0E-01 | 60.35 |
| 1.29E-02 | 9.13E-03 | 7.0E-01 | 4.7E-01 | 62.19 |
| 1.04E-02 | 7.34E-03 | 5.5E-01 | 3.8E-01 | 63.31 |
| 7.33E-03 | 5.45E-03 | 3.9E-01 | 2.9E-01 | 64.87 |
| 4.98E-03 | 3.54E-03 | 2.7E-01 | 1.9E-01 | 66.58 |
| 2.65E-03 | 1.81E-03 | 1.5E-01 | 1.0E-01 | 68.24 |
| 1.92E-03 | 1.43E-03 | 1.1E-01 | 8.0E-02 | 69.59 |
| 1.40E-03 | 1.03E-03 | 7.7E-02 | 5.7E-02 | 70.84 |
| 9.40E-04 | 6.79E-04 | 5.2E-02 | 3.8E-02 | 71.21 |
| 7.52E-04 | 5.00E-04 | 4.2E-02 | 2.8E-02 | 72.03 |
| 4.69E-04 | 3.34E-04 | 2.6E-02 | 1.9E-02 | 72.56 |
| 2.50E-04 | 1.79E-04 | 1.4E-02 | 9.9E-03 | 72.73 |
| 1.90E-04 | 1.36E-04 | 1.1E-02 | 7.5E-03 | 72.94 |
| 1.34E-04 | 8.21E-05 | 7.5E-03 | 4.6E-03 | 72.54 |
| 0.00E+00 | 0.00E+00 | 0.0E+00 | 0.0E+00 | 73.29 |

**Figure S21**: **Adsorption isotherms for glutaric acid + oxalic acid mixtures**: () Black curve: Oxalic acid; () blue curve: x = 0.25; () green curve: x = 0.4 () violet curve: x = 0.57 and () wine curve: Glutaric acid; Total acid concentration = [Glutaric acid] + [Oxalic acid].

### **Evolution of the surface tension for Glutaric and Oxalic acid mixtures**

**Figure S22:** **Evolution of the surface tension** **for glutaric + oxalic acid mixtures.** Measured at [total acid] = 0.5 M. x_Glutaric acid_ = glutaric acid molar fraction.

# **S8. Determination of the β^σ^ parameter for the mixtures Brij 35 + glutaric acid**

**Figure S23: determination of the β^σ^ parameter for the mixtures of brij35 + glutaric acid.**

In blue, X = 0.13; in red X = 0.0015.
